# Hyperspectral imaging for tumor resection guidance in surgery: a systematic review of preclinical and clinical studies

**DOI:** 10.1117/1.JBO.30.S2.S23909

**Published:** 2025-08-06

**Authors:** Antonio Composto, Laura Privitera, Martina Riva, Benedetto Ardini, Cristian Manzoni, Marco Riva, Kristian Aquilina, Gianluca Valentini, Stefano Giuliani

**Affiliations:** a Humanitas University, Department of Biomedical Sciences, Pieve Emanuele, Italy; b UCL Great Ormond Street Institute of Child Health, Cancer Section, Developmental Biology and Cancer Programme, London, United Kingdom; c University College London, Hawkes Institute, London, United Kingdom; d Politecnico di Milano, Department of Physics, Milan, Italy; e Istituto di Fotonica e Nanotecnologie-Consiglio Nazionale delle Ricerche (IFN-CNR), Milan, Italy; f IRCSS Humanitas Research Hospital, Department of Neurosurgery, Rozzano, Italy; g Great Ormond Street Hospital for Children NHS Trust, Department of Neurosurgery, London, United Kingdom; h Great Ormond Street Hospital for Children NHS Trust, Department of Specialist Neonatal and Pediatric Surgery, London, United Kingdom

**Keywords:** hyperspectral imaging, surgical oncology, artificial intelligence, image-guided surgery, fluorescence, optical biopsy

## Abstract

**Significance:**

Hyperspectral imaging (HSI) is a promising real-time, non-invasive, non-ionizing optical imaging technique. In surgical oncology, HSI can capture both structural and functional tissue information, allowing the characterization of tumor lesions both intraoperatively and on a histopathological level.

**Aim:**

We review the latest technological and clinical advancements of HSI as a guidance tool for tumor resection.

**Approach:**

Following the Preferred Reporting Items for Systematic Reviews and Meta-Analyses guidelines, we systematically searched MEDLINE, Embase, and Web of Science using logical keyword combinations related to “hyperspectral imaging” and “surgical oncology.” Eighty-five articles published between January 1, 2014, and April 30, 2024, were selected based on predefined inclusion and exclusion criteria. Technical and clinical data were extracted and analyzed.

**Results:**

The reviewed studies include preclinical and clinical investigations involving various tumor models and 2163 patients, including 24 pediatric cases. HSI has demonstrated broad applicability across various anatomical regions in both *ex vivo* and *in vivo* settings, with its most valuable application being tumor tissue delineation.

**Conclusions:**

HSI remains in its early technological stages, requiring high-quality evidence and multidisciplinary collaboration to support clinical adoption. A deeper understanding and improved characterization of biological tissue hyperspectral properties are essential to better inform and orient future hardware and software designs.

## 1 Introduction

Hyperspectral imaging (HSI) is an advanced optical imaging technique that captures spectroscopic data across multiple wavelengths for every point within the field of view (FOV) of an imaging device. This technology provides spatially resolved chemical and physical information, enabling the detection of subtle variations in tissue composition that are not visible to the human eye or conventional red-green-blue (RGB) cameras.^1^^,^^2^ Thanks to unprecedented progress in hardware and software, HSI is emerging as a cutting-edge, non-invasive medical imaging technique with numerous clinical applications.^1^^,^^3^^–^^6^ For instance, in surgical guidance, HSI can provide critical information about the malignancy risk of the tissue under investigation^7^^,^^8^ or assist with anatomical guidance by distinguishing vital structures, such as the nerves and vessels.^9^^,^^10^ The tissue’s spectral characteristics offer key insights into perfusion, oxygenation, and metabolism, making HSI particularly valuable for assessing flap viability^11^^,^^12^ and gastrointestinal anastomosis,^13^^–^^15^ potentially improving functional outcomes and reducing surgical complications. HSI can also be applied on a microscopic scale, providing an innovative method for capturing detailed physicochemical properties from tissue slides by measuring a wide range of spectral bands.^16^^,^^17^ In addition, HSI can potentially accelerate the histological diagnostic process by designing specialized pipelines that bypass some labor-intensive steps, such as staining, enabling faster clinical decision-making.^18^ Fu et al.^19^ have proposed using HSI for real-time assessment of hemodynamic and brain tissue changes in stroke. Since then, similar studies have extended this imaging approach to other neurological conditions, such as Alzheimer’s disease, where *in vivo* retinal HSI was investigated to predict brain amyloid-beta load.^20^^–^^22^ Other promising medical applications include early diagnosis of skin lesions,^23^ monitoring wound healing,^24^^,^^25^ endoscopic exploration,^26^^,^^27^ cardiovascular plaque characterization,^28^^–^^30^ and supporting drug development.^5^^,^^6^^,^^31^^,^^32^

This systematic review aims to collect and present preclinical and clinical studies that have used HSI to guide the visualization, characterization, and removal of tumors. We will explore the insights HSI can provide to surgeons and present an overview of the current state of research, development, and clinical application of HSI in surgical oncology, focusing on its effectiveness in surgical guidance. In addition, we will address existing limitations in HSI hardware, software, and scientific evidence, highlighting areas for improvement to support the optimization and implementation of this technology in clinical practice.

To ensure clarity, we begin with an overview of the relevant technical aspects, providing the necessary context for the subsequent discussion.

### 1.1 Hypercube

**Fig. 1:**
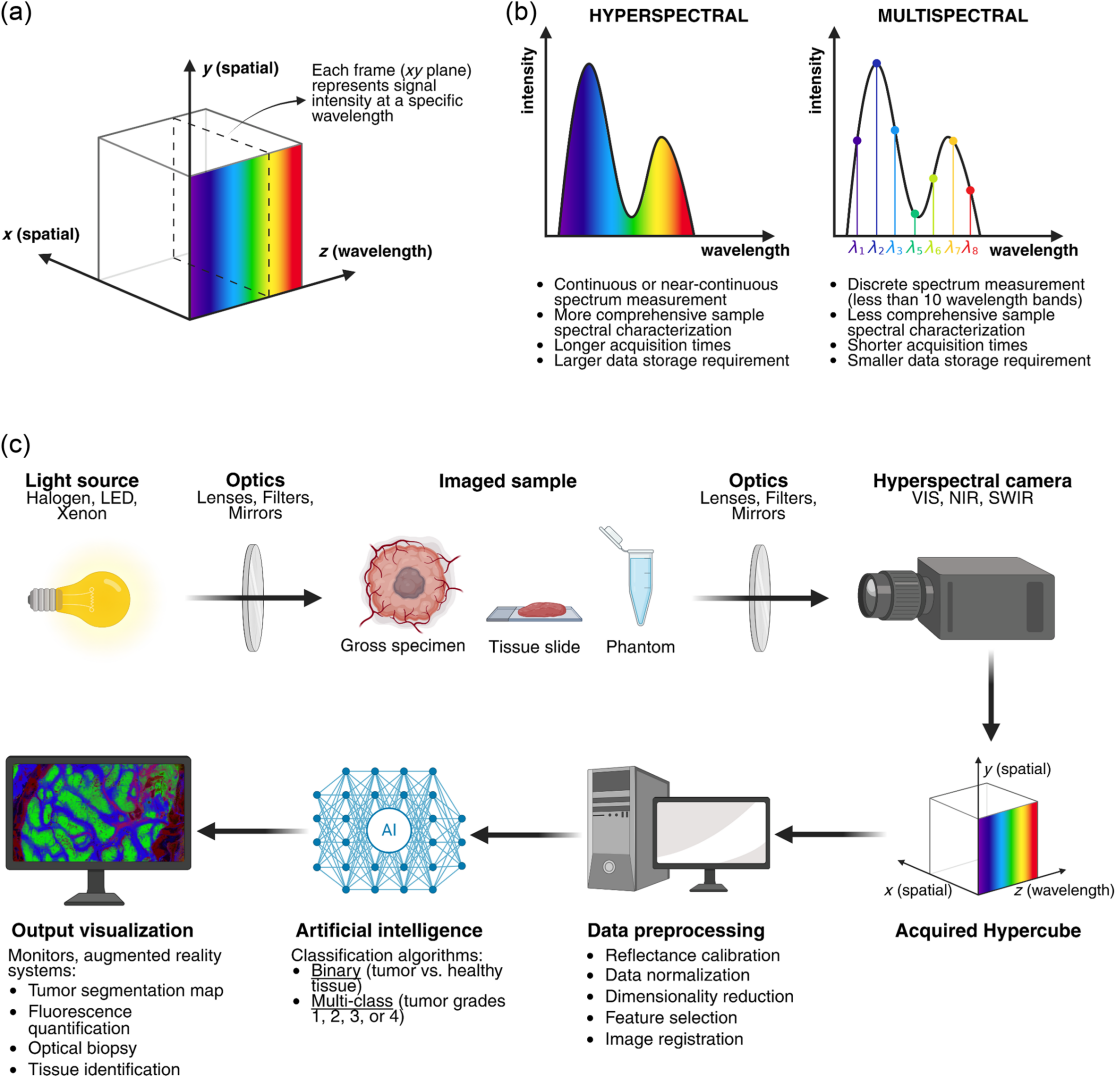
(a) Graphical representation of the hypercube, where x and y denote the spatial coordinates of the image frame, and z represents the measured wavelengths. Each image frame (dashed square) represents the signal intensity at a specific wavelength. (b) Two pictorial spectra are depicted to show the different wavelength acquisition depths (or sampling rates) in hyperspectral versus multispectral imaging. (c) Flow diagram of the hyperspectral imaging acquisition process of a biomedical sample.

The fundamental data acquired by a hyperspectral camera are referred to as the hypercube [Fig. 1(a)], a three-dimensional vector where the x and y axes represent spatial coordinates, and the z axis corresponds to the wavelength dimension. Generally speaking, HSI differs from multispectral imaging as the former implies data acquisition in a continuous or near-continuous range of wavelengths, eventually discretized in a large number of spectral bands, whereas multispectral imaging usually captures less than ten spectral bands [Fig. 1(b)]. Nonetheless, the rationale of hyperspectral and multispectral remains similar, and the two modalities can somehow complement each other, as we will explore further in Sec. 3.2.

### 1.2 Hyperspectral Imaging Acquisition Pipeline

During the acquisition of a hyperspectral image [Fig. 1(c)], the sample is illuminated by a specific light source, depending on the measurement mode (reflectance, fluorescence, and Raman). For reflectance acquisitions, a broadband (white) light source, such as a halogen, xenon, or light-emitting diode (LED) lamp, is used to ensure comprehensive spectral coverage. For fluorescence imaging, a narrow-band light source, coupled with specific excitation and emission filters, is generally preferred to target the excitation wavelength of specific fluorophores, minimizing background fluorescence from other endogenous or exogenous fluorophores.^33^^,^^34^ Examples of narrow-band light sources include LEDs, lasers, or broadband light sources combined with narrow bandpass filters. In contrast, Raman hyperspectral imaging uses a monochromatic laser to excite molecular vibrations within a tissue sample and detects the resulting inelastic scattering of incident photons, which causes wavelength shifts. However, this signal is inherently weak and prone to noise.^35^^–^^38^ Although Raman scattering is an analytical technique due to its chemical specificity, it can hardly be applied in real time, unless advanced techniques such as stimulated Raman are employed.

As the light interacts with a tissue, it is either reflected, absorbed, or transmitted, depending on the tissue’s optical properties, which are modeled by two wavelength-dependent coefficients, i.e., the absorption coefficient $\mu_{a}(\lambda )$ and the reduced scattering coefficient $\mu_{s}(\lambda )$. Considering a simplified corpuscular model for light, the first coefficient accounts for the absorption of photons by endogenous or exogenous chromophores within the tissue. The second is used to model the random walk of photons because of microscopic inhomogeneities that lead to local variations in the refractive index of the tissue. The measured signal is typically diffuse reflectance, which consists of incident photons that are backscattered by the tissue after penetrating to a depth determined by its optical properties. However, for thin tissue samples, such as histological sections, diffuse transmittance is also measured. Regardless of the parameter being detected, the detector captures a signal, known as radiance, across different wavelengths. The radiance L(x,y,θ,φ, and λ) describes the light emitted by a sample, capturing its spatial distribution (x and y) and angular spread (θ and φ) at any wavelength (λ).

To improve data quality and simplify the downstream analysis, a preprocessing chain is applied to the raw hypercube, as detailed in the sources.^35^^,^^36^ These pipelines may include steps such as reflectance calibration, data normalization, filtering, dimensionality reduction, data augmentation, feature extraction, feature selection, and image registration (e.g., alignment with ground-truth data). Following preprocessing, the signal goes through a data-processing pipeline, which often involves machine learning algorithms to extract useful information or generate data predictions. For example, an algorithm may classify a pixel’s signal as resembling either tumor or healthy tissue or predict whether a specific portion of a hypercube represents a nerve, vessel, or muscle. Alternatively, the signal may be decomposed into elementary spectra through unmixing algorithms, which can identify and localize specific molecular species of interest.^37^^,^^38^

These algorithms can vary significantly based on the input they receive and the output they are designed to deliver. For instance, spectral classification focuses on processing the spectral data from a single pixel to identify tissue type, whereas spectral-spatial classification considers both the pixel’s spectral data and its spatial relationships with neighboring pixels.^8^^,^^39^

### 1.3 Light-Tissue Interactions

The hypercube spectral information (z-axis) and spatial data (x-y axes) generate a detailed topographic map of the tissue’s molecular composition, providing insights into its morphology and metabolic processes.^40^^,^^41^ The optical properties of the sample are closely related to the presence and distribution of endogenous absorbers, scatterers, and fluorophores such as nicotinamide adenine dinucleotide phosphate hydrogen (NADPH), porphyrins, flavins, and collagen, among others.^34^^,^^42^^,^^43^ These components vary according to the tissue’s health or disease state, evolving in ways that reflect underlying pathological changes.^40^^,^^44^ A more detailed description of endogenous tissue fluorophores can be found in the review article by Croce and Bottiroli.^45^

### 1.4 Surgical Guidance for Tumor Resection

A major challenge in tumor resection is the risk of leaving behind residual tumor cells in the surgical bed, which can lead to disease recurrence and the need for additional interventions.^46^^–^^49^ Although recent efforts have expanded intraoperative tools for assessing tumor margins, each approach presents unique challenges related to surgical integration and clinical utility.

The current gold standard for intraoperative tumor margin assessment is frozen section analysis (FSA),^50^^–^^52^ which typically requires 30 to 45 min, resulting in significant workflow delays.^53^^–^^55^ In addition, FSA is susceptible to sampling errors and generally provides lower diagnostic quality than traditional pathology.^56^^,^^57^ Imaging-based alternatives such as intraoperative magnetic resonance imaging (MRI) and computed tomography (CT) offer excellent spatial resolution but lack real-time feedback.^58^ Intraoperative ultrasound, on the other hand, provides real-time information on tissue location, size, and shape.^59^ It also enables Doppler imaging to evaluate vascular structures and elastosonography to assess tissue stiffness.^60^^,^^61^ However, it remains highly operator-dependent, requires direct tissue contact, and often yields images that are difficult to orient within the surgical field.^62^ It may also produce artifacts, struggle to differentiate certain tumor subtypes,^63^ and show reduced sensitivity in detecting small residual tumors, particularly in brain surgery.^64^ Fluorescence-guided surgery (FGS) is a promising alternative that uses injectable fluorophores to illuminate and differentiate tumor tissue from surrounding healthy structures. Fluorescent contrast agents fall into two broad categories: untargeted agents (e.g., indocyanine green and methylene blue), which lack tumor specificity, and targeted agents conjugated to antibodies, peptides, or nanoparticles that bind tumor-specific markers.^65^ An exception is 5-aminolevulinic acid (5-ALA), which, though unconjugated, is selectively metabolized to protoporphyrin IX (PpIX) in gliomas, enabling tumor-specific fluorescence. Although several studies have shown that FGS improves the completeness of tumor resection,^65^^–^^67^ limitations remain. Not all tumors demonstrate adequate uptake of fluorescent agents,^68^ some agents pose toxicity risks,^68^^–^^70^ and most fluorescence signals are assessed qualitatively rather than quantitatively, introducing variability in interpretation^71^ and reducing diagnostic accuracy.^65^

HSI is emerging as a promising tool in this space, offering a relatively fast, non-invasive, non-ionizing, and potentially contrast-free approach, depending on its mode of operation. The same HSI system can acquire both reflectance and fluorescence data with minimal hardware and software modification,^3^ making it highly adaptable, particularly as new molecular probes are introduced.^72^ Moreover, HSI allows for a more detailed characterization of fluorescence signals, including the ability to distinguish autofluorescence and other spectral noise sources. As Mieog et al.^65^ noted, quantitative fluorescence imaging requires assessing local tissue optical properties,^73^ measuring the distance of every pixel in the image, and providing a perfectly flat illumination field (or calibrating for illumination heterogeneities). Although HSI does not meet all these requirements alone, it excels at characterizing tissue optical properties, providing essential data to support accurate fluorescence quantification.

HSI can be virtually applied to any anatomical region in both adult and pediatric patients.^38^^,^^74^^,^^75^ Over the years, various HSI designs have been developed to meet the needs of different medical and surgical applications. The most common are camera-based systems, which provide a wide-field view of the observed area and can be integrated with surgical or laboratory microscopes for microscopic hyperspectral imaging, enabling detailed tissue analysis.^75^^,^^76^ In addition, probe-based HSI systems have been developed, particularly for minimally invasive surgery or endoscopic exploration.^1^^,^^9^

The increasing predictive power of artificial intelligence algorithms could significantly enhance HSI’s capabilities by uncovering critical tumor information and identifying novel biomarkers.^4^^,^^77^ For instance, advanced artificial intelligence algorithms can help detect tumor margins, assess vascularization, metabolism, and tissue oxygenation; determine histologic type and grade; and quantify the concentration of specific clinically relevant biomolecules, including fluorophores.

## 2 Methods

### 2.1 Search Strategy

**Fig. 2:**
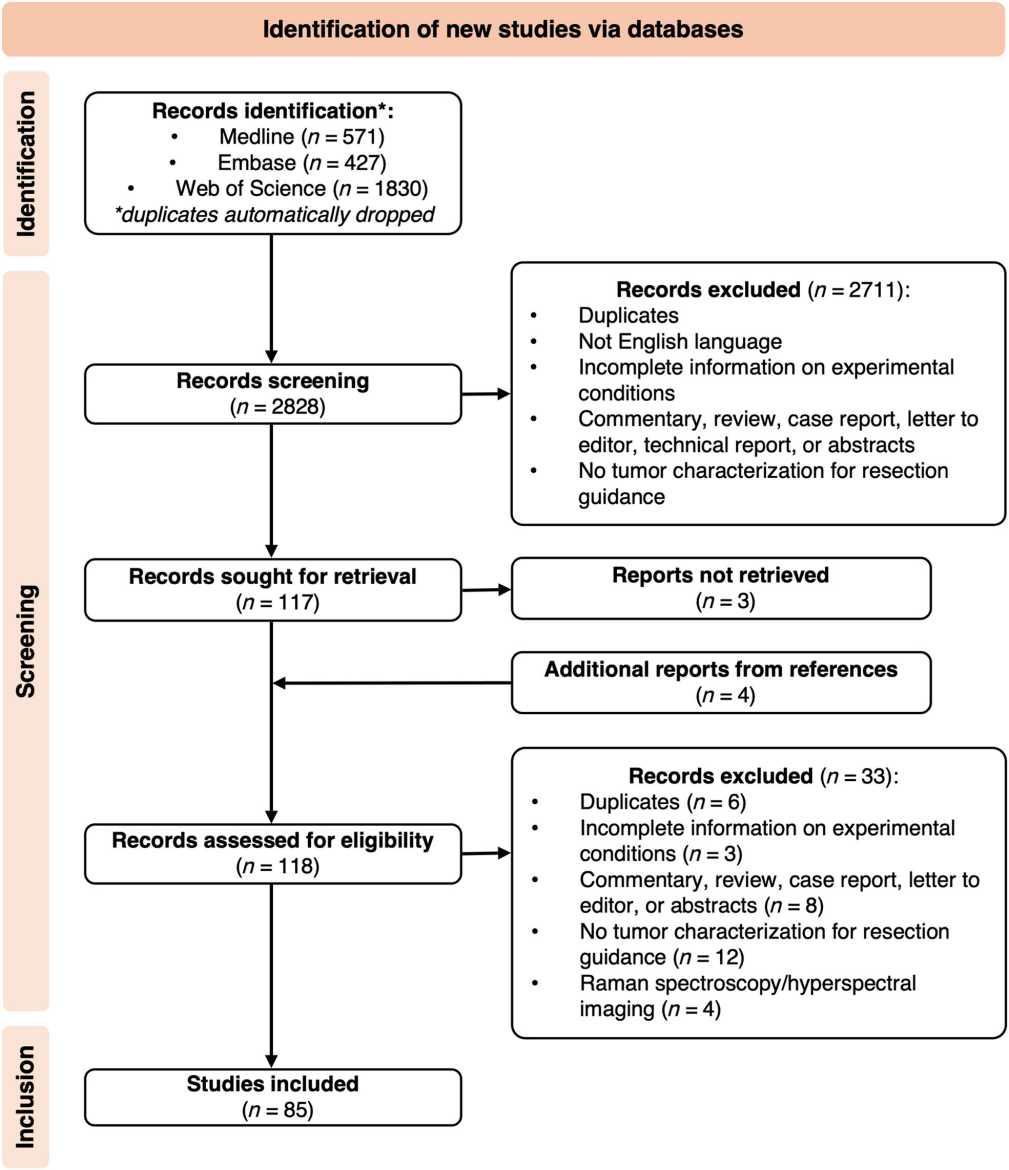
PRISMA flow diagram for the study selection.

This review was conducted in accordance with the Preferred Reporting Items for Systematic Reviews and Meta-Analyses (PRISMA) guidelines^78^ (Fig. 2). A systematic search was carried out using MEDLINE, Embase, and Web of Science databases, covering the period from January 1, 2014, to April 30, 2024, with an automatic de-duplication filter applied. We decided to focus on the literature from the past decade, building on the foundational work done by Lu and Fei^79^ and Clancy et al.^1^ A search string was developed and adapted to each database (see Table S1 in the Supplementary Material) by logically combining keywords around the field of HSI-guided surgical resection of low-depth cancerous lesions. The initial search was conducted on October 9, 2023, and subsequently updated on May 10, 2024, following the same search strategy across all three databases. In total, 2828 records were identified.

### 2.2 Study Selection

The titles and abstracts were independently screened by two authors (A.C. and L.P.) against a set of predefined inclusion and exclusion criteria (see Table S2 in the Supplementary Material). Any disagreements were resolved through full-text revision and consultation with five senior reviewers (S.G., K.A., G.V., C.M., and M.R.). We initially removed duplicate articles not caught by the automatic de-duplicator and excluded articles that were not in English, lacked sufficient information on experimental conditions, were unrelated to the tumor characterization for resection guidance, and were in the form of commentaries, reviews, case reports, technical reports, letters to the editor, or abstract-only publications. At this stage, 117 reports were sought for retrieval. Three reports could not be retrieved, and four additional reports were identified from reference lists. In total, 118 reports were assessed for eligibility through full-text reading, with 33 subsequently excluded based on the exclusion criteria. We excluded studies involving Raman imaging due to their small number (n=4) and the intrinsically different setup and acquired data, which would require a separate review. A total of 85 studies were included in this review (see Table S3 in the Supplementary Material).

### 2.3 Data Extraction

Data were extracted across two categories of variables. The first category focused on the technical specifications of the hyperspectral imaging system, data processing, and analysis. These variables include specifications such as manufacturer, model, sensor, acquisition method, wavelength range, number of spectral bands, FOV, dispersive element, spectral resolution, spatial resolution, temporal resolution, data cube acquisition time, light source, and data analysis method. The second category focused on clinical data and experimental settings. This included information on whether image acquisition was intra- or extra-operative, the number of patients involved, population type, sample type, whether samples were *in vivo* or *ex vivo*, tissue preparation methods, fluorescence or reflectance mode, and the clinical application. For each study, we also collected the performance metric associated with the data analysis methods used for specific predictive tasks, such as binary tumor classification.

## 3 Results

### 3.1 Overview of Selected Studies

Among the selected studies, HSI was employed in various applications, including tumor segmentation, fluorescence analysis, tumor characterization (e.g., predicting tumor grade or type), tissue identification, and augmented reality. A summary of these applications is provided in Table 1.

**Table 1:** Distribution of selected studies and the corresponding number of patients, categorized by the applications of hyperspectral imaging in surgical oncology.

Clinical application	Number of studies reporting the application				Number of patients
	Preclinical	Clinical	Mixed^a^	Total	
Tumor segmentation	8	64	2	**74**	**1569**
Fluorescence analysis^b^	3	7	3	**13**	**550**
Tumor characterization (grade/type prediction)	0	4	0	**4**	**139**
Tissue identification	0	4	0	**4**	**88**
Augmented reality	1	1	0	**2**	**0**
Total	11^c^	69^c^	5^c^	**85** ^c^	**2163** ^c^

a Mixed refers to those studies that included both preclinical and clinical components.

b Includes both autofluorescence (endogenous) and exogenous fluorescence (e.g., contrast agents such as 5-ALA and proflavine).

c Frequency value is corrected for possible overlapping studies (i.e., if one study involves both a tumor segmentation and augmented reality application, these are counted as a single study in total—the same holds for the number of patients).

Note: Bold values represent the total number of studies and patients for each respective category.

Our literature search identified 16 preclinical studies conducted on phantoms, animals, or both, emphasizing the early-stage development of HSI in surgical oncology (Table 2). These studies provide invaluable insights to guide future research and support the clinical implementation of HSI. In addition, 74 clinical studies were identified, all focusing on the use of HSI in surgical oncology. These studies involved a total of 2163 patients, of whom only 24 were pediatric patients from 3 neurosurgical studies on pediatric brain tumors.^38^^,^^74^^,^^75^ However, patient numbers may be inaccurate due to incomplete reporting and potential overlaps among patients across studies that could not be unequivocally identified. In terms of experimental settings, 39 clinical studies were conducted in an extra-operative environment, 29 were performed intraoperatively, 1 involved both intra- and extra-operative settings, and 5 did not specify the setting. Most protocols tested HSI technology on *ex vivo* tissue samples (n=53), with fewer studies focusing on *in vivo* samples (n=23).

**Table 2:** Summary table of the selected preclinical and mixed (preclinical and clinical) studies.

Reference	Study	Sample	Anatomic region	Setting	Preparation	Signal	Sensor	Wavelength range	Number of spectral bands	Light source	Data analysis method	Performance
Tumor segmentation												
Huang et al.^80^ ^a^	Preclinical	Phantom	Brain	Phantom	Colorless gelatin representing healthy brain tissue; yellow-dyed gelatin simulating tumor; porcine blood resembling blood vessels	Reflectance	Na	485 to 900 nm	150	Na	Shallow neural network	Na
Kho et al.^81^	Mixed	Phantom; human	Breast	Phantom; *ex vivo*	A black container with eight black polyoxymethylene rods of varying heights was filled with a fat emulsion consisting of a mixture of 1 part intralipid 20% and 19 parts deionized water	Reflectance	VIS: CMOS; NIR: InGaAs	VIS: 450 to 951 nm; NIR: 954 to 1650 nm	VIS: 318; NIR: 210	Halogen	Proposed method “spectral slope method”	Na
					*Ex vivo* sample: fresh							
Lu et al.^82^	Preclinical	Animal (mouse)	Head and neck	*In vivo*; *ex vivo*	Fresh head and neck squamous cell carcinoma tumor xenograft model (M4E cells labeled with green fluorescence protein)	Reflectance	CCD	450 to 950 nm	251	Xenon	Na	Na
Lu et al.^35^	Preclinical	Animal (mouse)	Head and neck	*In vivo*	Same as Ref. 82	Reflectance	CCD	450 to 900 nm	226	Xenon	Support vector machine classifier	Sensitivity: 94.4%; specificity: 98.3%
Lu et al.^36^	Preclinical	Animal (mouse)	Head and neck	*In vivo*	Same as Ref. 82	Reflectance	CCD	450 to 900 nm	226	Xenon	K-nearest neighbors	Accuracy: 67.2%; sensitivity: 77.5%; specificity: 54.0%
Ma et al.^83^	Preclinical	Animal (mouse)	Head and neck	*In vivo*	Same as Ref. 82	Reflectance	CCD	450 to 950 nm	251	Xenon	Autoencoder; convolutional neural network	Sensitivity: 92.32%; specificity: 91.31%
Ma et al.^84^	Preclinical	Animal (mouse)	Head and neck	*In vivo*	Same as Ref. 82	Reflectance	CCD	450 to 950 nm	251	Xenon	Convolutional neural network	Accuracy: 91.36%; sensitivity: 86.05%; specificity: 93.36%
Lu et al.^8^	Preclinical	Animal (mouse)	Head and neck	*In vivo*	Same as Ref. 82	Reflectance	CCD	450 to 900 nm	224	Broadband	**Support vector machine classifier**; K-nearest neighbors	Sensitivity: 93.7%; specificity: 91.3%
Stewart et al.^85^	Mixed	Animal (mouse); human	Breast; lung; kidney	*In vivo*; *ex vivo*	Mouse model: subcutaneously inoculated with patient-derived IDC or lung adenocarcinoma tumors	Reflectance	CCD	520 to 1050 nm	141	Halogen	**Partial least squares discriminant analysis**; ratiometric score	*In vivo*: AUC: 96%;*Ex vivo*: accuracy: 89%; sensitivity: 100%; specificity: 83%
					*Ex vivo* sample: fresh							
Mun et al.^86^	Preclinical	Animal (mouse)	Pancreas	Phantom; *in vivo*	Fresh pork tissue phantom; fresh orthotopic mice pancreatic tumor models from KPC and Pan02 cell lines	Reflectance	CCD	420 to 730 nm	Na	LED	Support vector machine **classifier**; shallow neural network; **light gradient boosting** machine	KPC tumor: precision [LGBM]: 89.6%; recall [LGBM]: 61.7%; F-score [LGBM]: 73.1%Pan02 tumor: precision [SVM]: 83.0%; recall [SVM]: 50.9%; F-score [SVM]: 63.1%
Fluorescence analysis												
Bravo et al.^3^	Mixed	Phantom; human	Brain	Phantom; *in vivo*	Liquid phantoms: varying PpIX concentrations, lipid and blood volume fractions.*Ex vivo* sample: fresh	Fluorescence (5-ALA); reflectance	CMOS	F: 600 to 720 nmR: 440 to 720 nm	Na	Xenon; blue light fluorescence filter (Zeiss BLUE 400)	Spectral fitting; spectral unmixing	Na
De Landro et al.^87^	Preclinical	Phantom	Na	*In vitro*	Fluorescently labeled antibodies dissolved in phosphate-buffered saline (PBS)	Fluorescence (Alexa Fluor 647 Dye)	CMOS	500 to 995 nm	100	Halogen	Na	Na
Lehtonen et al.^33^	Preclinical	Phantom	Brain	Phantom	A heated saline and gelatin mixture was cooled and combined with varying PpIX concentrations dissolved in dimethyl sulfoxide (DMSO) then mixed with Intralipid 20%	Fluorescence (5-ALA)	CMOS	510 to 903 nm	4	Xenon; blue light fluorescence filter (Leica FL400)	Classifier (visible versus non-visible fluorescence)	Recognition rate: 96%; sensitivity: 100%; specificity: 86%; positive predictive value: 94%; negative predictive value: 100%
Meng et al.^88^	Preclinical	Phantom; animal (mouse)	Breast	Phantom; *ex vivo*	Liquid phantoms: containing intralipid 1% in PBS and varying concentrations of quantum dots (QD-605 and QD-655)	Fluorescence (QD)	CMOS	500 to 700 nm	40	LED	Na	Na
					*Ex vivo* sample: fresh							
Walke et al.^89^	Mixed	Phantom; animal (pig); human	Brain	Phantom; *ex vivo*	Pig brain homogenates: first buffered with 0.5-M trishydroxymethylaminomethane or directly spiked with varying PpIX concentrations dissolved in DMSO	Fluorescence (5-ALA)	CMOS	420 to 730 nm	Na	LED; blue light fluorescence filter (Zeiss BLUE 400)	Na	Na
					Liquid phantoms: McCormick dye and intralipid with varying PpIX concentrations							
					*Ex vivo* sample: fresh							
Suero Molina et al.^90^	Mixed	Phantom; animal (pig); human	Brain	Phantom; *ex vivo*	Same as Ref. 91	Fluorescence (5-ALA)	CMOS	420 to 730 nm	Na	LED; blue light fluorescence filter (Zeiss BLUE 400)	Spectral unmixing	Na

No preclinical or mixed studies were identified for “tumor characterization” and “tissue identification” applications.

The bold data analysis methods indicate, in studies with multiple methods, the one that performed best (performance metrics shown in the adjacent columns).

a Although this study belongs to the “tumor segmentation” class, it also focuses on augmented reality development.

**Fig. 3:**
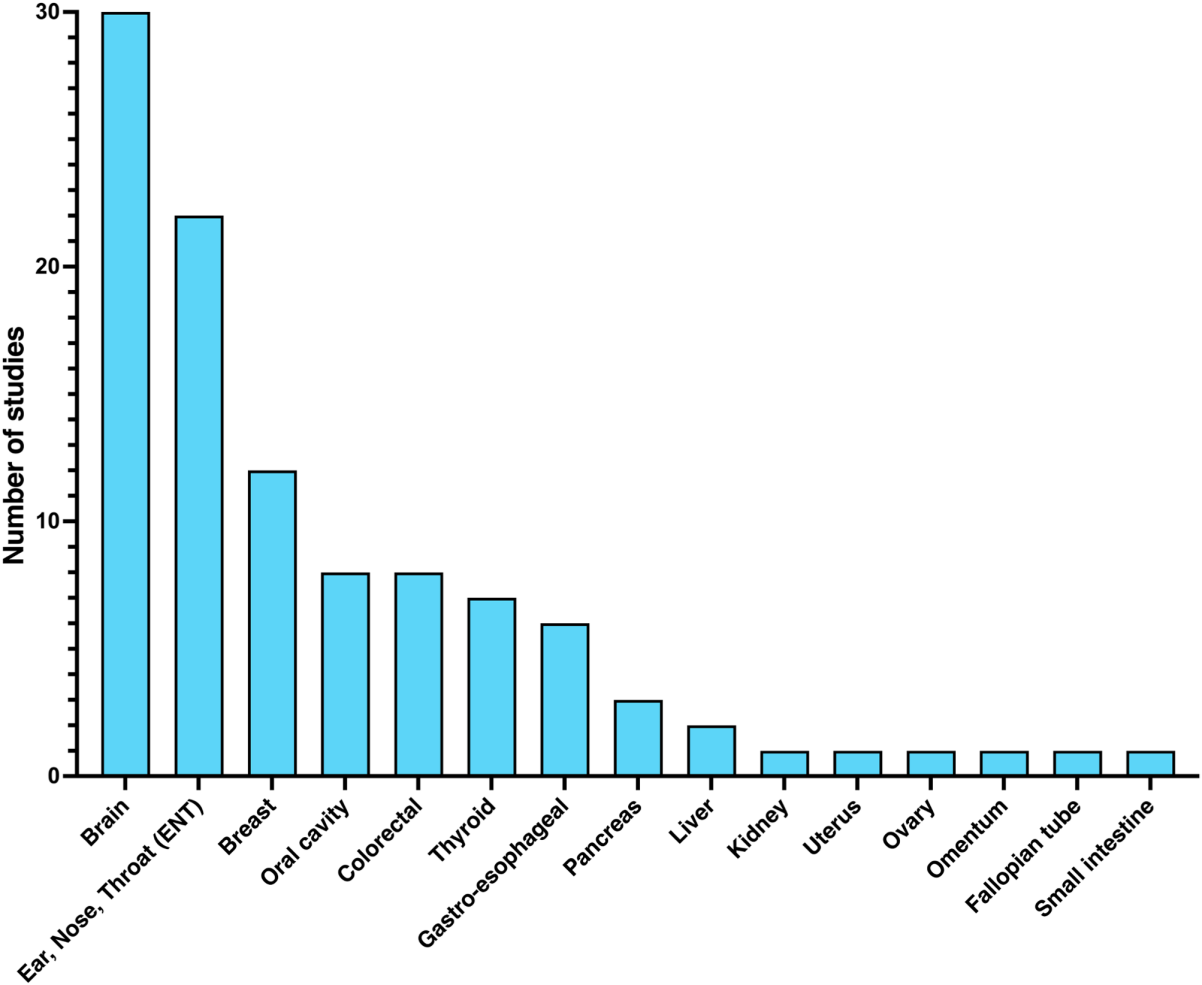
Bar plot showing the number of clinical studies applying HSI on the listed tissue types (*in vivo* and *ex vivo*).

The clinical studies reviewed research applied HSI to a diverse range of tissues (Fig. 3), demonstrating its versatility across different histologic profiles and anatomical regions. Tissue samples included both healthy tissue parenchyma and tumor sites in both *in vivo* and *ex vivo* applications. Neurosurgery accounted for the largest portion of these studies (n=30), focusing on adult and pediatric brain tumors such as glioblastoma multiforme, low-grade glioma, grade III astrocytoma, meningioma, pituitary adenoma, medulloblastoma, anaplastic ependymoma, and pilocytic astrocytoma. Another 22 studies focused on the head and neck region, primarily investigating squamous cell carcinoma affecting sites such as the pharynx (nasopharynx, oropharynx, and hypopharynx), larynx, maxillary sinus, and oral and nasal cavities. Two studies examined tumors of the parotid and other salivary glands, whereas seven articles focused on thyroid lesions, including papillary thyroid carcinoma, medullary thyroid carcinoma, and follicular adenoma. Twelve studies investigated breast tissue, including conditions such as invasive ductal carcinoma (IDC) and ductal carcinoma *in situ* (DCIS). Eight studies focused on colorectal cancer, whereas seven explored gastroesophageal tumors. Pancreatic cancer was investigated in three studies, whereas hepatocellular carcinoma was studied in two. In addition, only one study was identified for each malignancy: kidney, small intestine, omentum, uterus, ovary, and fallopian tube.

### 3.2 Summary of the Technical Specifications

**Fig. 4:**
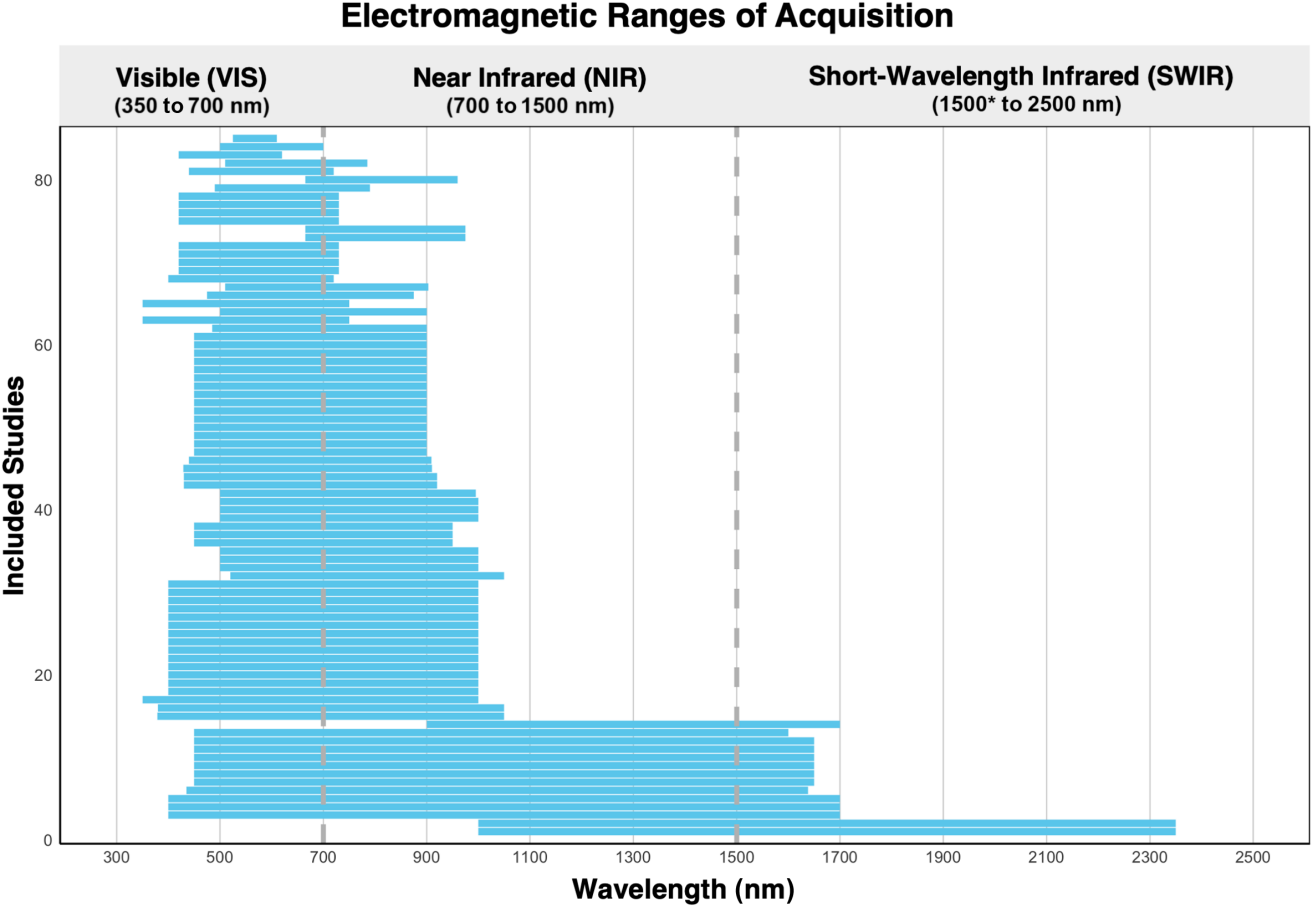
Graph illustrating the wavelength ranges analyzed in each of the 85 studies included in this review. The VIS is represented between 350 and 700 nm, the NIR region spans from 700 to 1500 nm, and the SWIR extends from 1500 to ∼2500  nm. It should be noted that these ranges are not fixed, as varying conventions exist for defining these spectral boundaries. *The lower wavelength bound for the SWIR region is indicated here as 1500 nm by convention, although some definitions begin at 900 nm. This ambiguity arises from the lack of a universally accepted standard, often resulting in overlap between the NIR and SWIR regions.

The technical specifications of the reviewed HSI systems were compiled in a spreadsheet and compared across studies. A key focus was on the range of wavelengths analyzed, as different spectral regions can provide distinct biological insights and may be influenced by various noise sources. For each study, Fig. 4 presents the analyzed wavelength range, which may have been narrowed following pre-processing or band selection steps. For example, in some cases, hyperspectral camera sensors exhibit reduced sensitivity at the extreme ends of the measurable spectrum, leading to the exclusion of those regions.^7^^,^^9^^,^^70^^,^^92^ In other cases, optimization algorithms were employed to select the most predictive wavelengths for specific classification tasks, thereby reducing computational complexity and processing time. For instance, Martinez et al.^93^ investigated the impact of different sampling strategies to reduce the number of spectral bands used in brain tumor classification. They found that certain wavelengths primarily contained noise and exhibited data redundancy. By applying a genetic algorithm-based optimization methodology, they were able to eliminate such bands, improving tumor identification accuracy by ∼5%. This improvement was achieved using only 48 spectral bands, compared with the original 128 bands. Among the 85 studies reviewed, 20 explicitly reported reducing the number of spectral bands for similar purposes.

Most studies focus on the visible spectrum (VIS) and the lower portion of the near-infrared spectrum (NIR), typically up to 900 nm. Only a small proportion (16%) explore wavelengths in the upper NIR and short-wave infrared region (SWIR) regions, typically up to 1700 nm, with two studies extending to 2350 nm. For instance, Mitsui et al.^94^ utilized NIR-HSI beyond 1000 nm to assess gastrectomy margins in gastric cancer. Because white light has limited penetration depth, detecting residual disease endoscopically in areas not exposed to the mucosal surface is challenging. Using longer wavelengths, which penetrate deeper into the mucosa due to reduced absorption and scattering, the authors collected surgical specimens of healthy and tumor tissues, including non-exposed cancer samples. Their support vector machine (SVM) classifier achieved an average accuracy of 77.2%, successfully identifying unexposed cancer areas when the tumor was 2 mm or greater. In general, studies that acquire data across broad wavelength ranges often use multiple sensor materials, each tailored for a specific portion of the electromagnetic spectrum. Silicon-based sensors, such as charge-coupled devices (CCDs) and complementary metal-oxide-semiconductor (CMOS) detectors, are typically used for measuring radiation in the visible and lower NIR regions (∼400 to 900 nm) due to their affordability and availability. In contrast, indium gallium arsenide (InGaAs) sensors, which are considerably more expensive, enable detection in the SWIR region (∼900 to 1700 nm). For example, Baltussen et al.^9^ combined two hyperspectral cameras - one with a CMOS sensor for the VIS to the lower NIR range and another with an InGaAs sensor for the SWIR range - covering a total spectral range of 400 to 1700 nm. Among the studies reviewed, CCD sensors were the most commonly used (n=42), followed by CMOS sensors (n=32) and InGaAs sensors (n=12). Regarding illumination sources, halogen lights were the most frequently employed (n=40), followed by xenon lights (n=15) and LEDs (n=9). These findings reflect the diversity in technical setups and highlight the importance of tailoring HSI systems to specific applications.

### 3.3 Tumor Segmentation (Reflectance)

In image-guided surgery, “tumor segmentation” refers to creating a spatial map that delineates the tumor’s boundaries from the surrounding tissues. These maps, generated by advanced computer vision algorithms, help surgeons achieve more precise tumor resection by highlighting tumor regions that may not be visible to the naked eye or conventional instruments. HSI enables the delineation of tumor contours by identifying pixels corresponding to tumor tissue through reflectance measurements or the characteristic fluorescence signal, which may be enhanced using fluorescent contrast agents. This section focuses on studies investigating tumor segmentation based on reflectance measurements. Fluorescence-based methods will be discussed in Sec. 3.4.

#### 3.3.1 Preclinical studies on tumor segmentation (reflectance)

Preclinical studies represent a fundamental step in the clinical translation of novel intraoperative imaging modalities, enabling the assessment of their safety, feasibility, and efficacy. As detailed in Table 2, typical experimental setups in this context include liquid, gel, or solid phantoms; mouse models bearing tumor xenografts of various histologies; and/or animal *ex vivo* specimens.

**Fig. 5:**
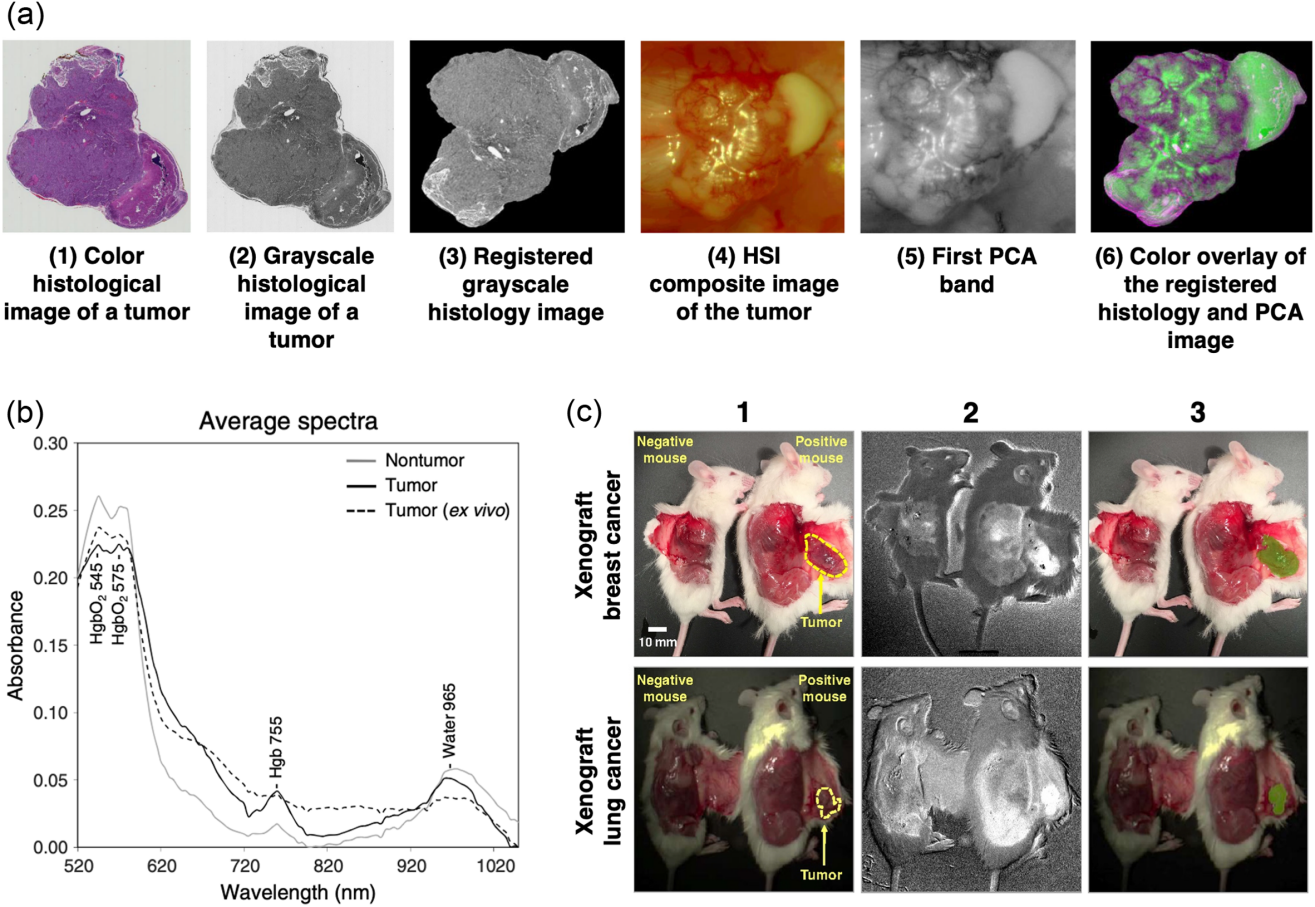
(a) Registration of hyperspectral and histological images. Reproduced with permission, courtesy of Lu et al.^82^ (b) PLS-DA model for tumor *in vivo*. Average absorption spectra representing *in vivo* and *ex vivo* tumor and *in vivo* nontumor tissues. Reproduced with permission, courtesy of Stewart et al.^85^ (c) Molecular chemical imaging (MCI) score images of breast and lung cancer in mice *in vivo*. (1) Annotated RGB images of mice with (positive) and without (negative) xenografts. The tumors, located on the exposed skin flap (subcutis) near the flank, are annotated in yellow. (2) Ratiometric score images for the lung cancer model (735/975) and the breast cancer model (1035/625). (3) Tumor detections (in green) from ratiometric score images overlaid onto RGB images. Reproduced with permission, courtesy of Stewart et al.^85^

Evaluating the efficacy of HSI in segmenting tumor tissue crucially begins with its comparison to a ground-truth map, typically derived from a gold-standard technique such as histopathology. Lu et al.^82^ demonstrated the feasibility of registering *in vivo* surgical hyperspectral “macro” images with *ex vivo* histological “micro” images, which served as the ground truth for tumor segmentation [Fig. 5(a)]. The raw hyperspectral data were fed into a dimensionality reduction algorithm using principal component analysis and combined with the histological data through affine registration and B-spline free-form deformation. The proposed method achieved a high overlap accuracy (Dice similarity coefficient >98%) and low target registration error (below 0.21 mm).

A substantial body of work has focused on employing HSI for the *in vivo* detection of tumors in mouse models. Stewart et al.^85^ assessed 12 mice inoculated with patient-derived breast and lung tumor xenografts, generating six hypercubes (spectral range: 400 to 1100 nm, 5-nm intervals) from both tumor-bearing and tumor-free mice. The spectra from the tumor and healthy tissue were pre-processed, and tumor segmentation was obtained using either partial least squares discriminant analysis (PLS-DA) or a ratiometric approach. PLS-DA is a robust multivariate statistical tool that analyzes a broader region of the acquired electromagnetic spectrum, offering greater accuracy but more processing time. In contrast, the simpler and faster ratiometric approach relies on the ratio of two spectral bands to maximize contrast between tumor and healthy tissues. PLS-DA demonstrated high effectiveness in distinguishing *in vivo* tumor spectra from healthy tissue (sensitivity: 100%, specificity: 83.3%, accuracy: 88.9%, and area under the receiver operating characteristic curve (AUROC): 0.917). Although the ratiometric approach is much faster, it resulted in a significantly poorer signal-to-noise ratio. Furthermore, the utility of coupling HSI with a ratiometric method remains questionable, as this approach inherently excludes all wavelengths not specifically selected for the ratio from subsequent analysis. This limitation suggests that the ratiometric method might be a more suitable alternative for multispectral imaging, where data acquisition is inherently restricted to a discrete set of pre-selected, highly informative wavelengths, and their ratios could effectively enhance tissue contrast. The analyzed spectral signatures revealed the key differences between tumor and non-tumor tissues [Figs. 5(b) and 5(c)]. Non-tumor tissues showed prominent water absorption peaks at 965 nm and oxygenated hemoglobin peaks at 545 and 575 nm, reflecting their high-water content and vascularity. In contrast, tumor spectra showed a higher contribution of deoxygenated hemoglobin, peaking at 755 nm, reflecting the hypoxic tumor microenvironment. The study also noted weaker absorption peaks for water and hemoglobin in *ex vivo* samples compared with *in vivo* ones, likely due to reduced blood flow, lower oxygen levels, and decreased water content. Nonetheless, highlighting the similarities between *in vivo* and *ex vivo* spectra could also be valuable. These potential common spectral features might identify components within *ex vivo* HSI data that could benefit the training or calibration of *in vivo* hyperspectral classification algorithms, particularly given the difficulty in creating large *in vivo* HSI databases.

Notably, the algorithms discussed so far have relied solely on spectral information for prediction, thus ignoring image spatial nuances that could aid in differentiating tumor from healthy tissue. In conventional spectral approaches, the hypercube is often vectorized into a two-dimensional matrix, which results in the loss of spatial information. To overcome this limitation, Lu et al.^8^ transformed the hypercube into a spectral - spatial tensor representation, preserving the spatial relationships. Tensor decomposition was used to extract important features and reduce dimensionality, followed by an SVM classifier to create a tissue prediction map, reaching a sensitivity of 93.7% and a specificity of 91.3%. Lu et al.^35^ also proposed a wavelength optimization technique called maximal relevance and minimal redundancy to select the most informative wavelengths for differentiating tumors from healthy tissues. This wavelength selection step is applied before the classification algorithm to help mitigate the Hughes phenomenon,^95^ where an excessive number of input features reduces overall classification accuracy. Although reducing the number of selected wavelengths led to higher error rates, it significantly decreased processing time and storage requirements, highlighting the potential for optimizing the trade-off between accuracy and efficiency.

Currently, spectral-spatial classification is commonly implemented using various algorithmic approaches, including traditional machine learning methods combined with spatial feature extraction (as exemplified by Lu et al.^8^), deep learning techniques, and hybrid frameworks. Among these, convolutional neural networks (CNNs) are perhaps the most prevalent due to the inherent suitability of their convolutional filters for extracting meaningful spectral - spatial features from image data. The intrinsically high-dimensional nature of hyperspectral data is driving a transition in feature extraction from handcrafted methods, such as first- and second-order derivatives of spectral curves, Fourier coefficients, and mean/total reflectance, toward CNN-derived features. These learned features are reported to be faster to compute, more precise, and discriminative.^84^ In this context, Ma et al.^84^ employed CNNs to extract features from hyperspectral reflectance images of 12 mice bearing GFP-positive head and neck squamous cell carcinoma xenografts, using GFP fluorescence as the ground truth. Subsequent work further advanced the classification algorithm through the implementation of an unsupervised adaptive auto-encoder network. This network progressively refined its accuracy by adjusting weights based on initial detection results, prioritizing the most relevant features for cancer detection.^83^ This optimized approach yielded average performance metrics of 92.32% sensitivity, 91.31% specificity, and 91.33% accuracy.

Finally, Mun et al.^86^ presented another interesting preclinical study detailing the development of an endoscopic HSI system. They validated this system on fresh pork tissue phantoms and orthotopic pancreatic tumor models in mice (KPC and Pan02 cell lines), acquiring hyperspectral images across a wavelength range of 420 to 730 nm, with 10-nm intervals. Among the various tumor classification algorithms evaluated, the light gradient boosting machine yielded the best performance on the KPC cell line (precision: 89.6%, recall: 61.7%, and F1-score: 73.1%), whereas the support vector machine classifier achieved the highest performance on the Pan02 cell line (precision: 83.0%, recall: 50.9%, and F1-score: 63.1%). A significant strength of the described setup lies in its potential to investigate deep-seated organs that may be inaccessible to external surgical cameras. The application of HSI in surgical endoscopy remains a comparatively underdeveloped field compared with its use with external imaging systems. However, we contend that dedicated research and development in endoscopic HSI are crucial, given the paramount importance and routine utilization of endoscopes in surgery.

#### 3.3.2 Clinical studies on tumor segmentation (reflectance)

Clinical studies investigating the application of HSI for tumor segmentation can be broadly classified into *ex vivo* and *in vivo* studies. *Ex vivo* studies typically involve imaging-excised tumor specimens, whereas *in vivo* studies focus on intraoperative imaging during surgery.

##### *Ex vivo* (reflectance): an example from breast surgery

There are two main reasons for imaging *ex vivo* tissue samples. First, the intended use of HSI may be limited to scanning the resected specimen to evaluate resection quality. Second, even when the ultimate goal of HSI is real-time intraoperative imaging, *ex vivo* samples closely mimic the optical properties of *in vivo* tissue, despite exhibiting some differences, primarily due to reduced blood flow, lower oxygen levels, and decreased water content, as previously discussed in Sec. 3.3.1.

**Fig. 6:**
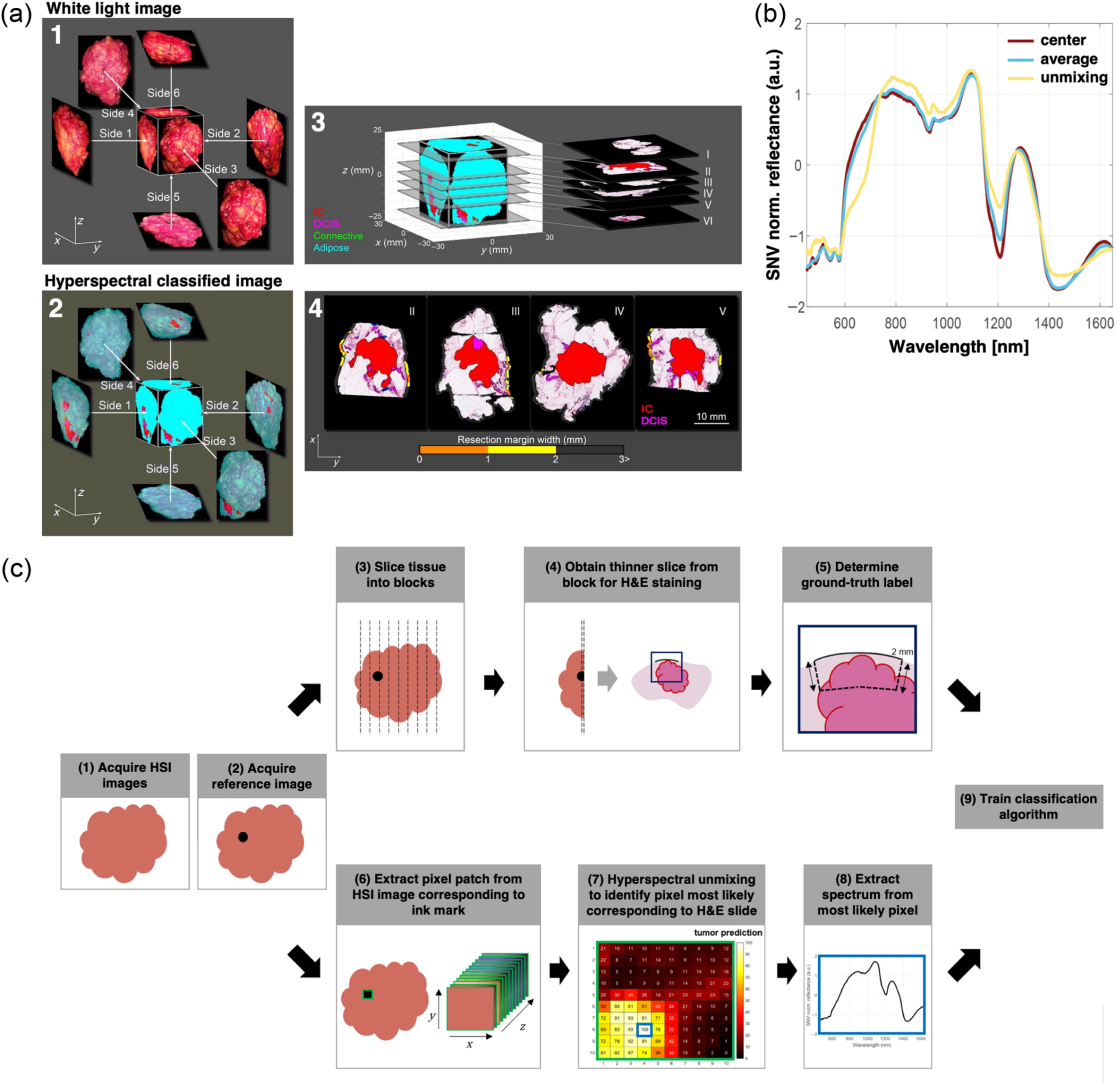
(a) Representative example of HS analysis on resection surface. Both white light (WL) (1) and HS images were taken from six sides so that the entire resection surface was imaged. (2) Classification result of the HS images using the SVM algorithm, developed with the tissue slices. The specimen was sliced according to standard pathology protocol, and six H&E sections were processed for further analysis. (3) The orientation of these sections with respect to the 3D representation of the HS classified specimen. H&E sections 1 and 6 were taken parallel to the resection surface and hence do not provide information on the margin width. Therefore, these sections were excluded from the analysis. (4) In H&E sections 2 to 5, an experienced pathologist annotated the tumor so that the resection margin width could be assessed. Reproduced with permission, courtesy of Kho et al.^96^ (b) Standard normal variate (SNV)-normalized diffuse reflectance spectra of three different approaches for selecting pixels to assign ground-truth labels. There is a distinct difference in the SNV-normalized spectrum obtained with hyperspectral unmixing (pixel with the highest tumor value in the prediction map) compared with the center pixel and the average of all pixels in the patch. Reproduced with permission, courtesy of Jong et al.^7^ (c) Pipeline to train a tissue classification algorithm where ground-truth labels are assigned to the training set based on hyperspectral unmixing. (1) First, the hyperspectral images of the specimen are acquired. Subsequently, black ink marks are placed on the specimen, and a reference image (2) is captured to correlate the hyperspectral images to the histology slides. The upper pipeline shows the histopathological workflow, where (3) the lumpectomy specimen is gross-sectioned into tissue blocks of a few millimeters thick; (4) a slice of ±4  μm thick is sliced from this block and (5) stained with H&E, digitized and inspected by a pathologist who delineates the malignant tissue region in red up to a depth of 2 mm below the inked surface. The lower pipeline shows the workflow to determine which pixel covered by the ink mark should be assigned to this ground-truth label. (6) A patch of 10 by 10 pixels is extracted for each ink mark; (7) based on hyperspectral unmixing, a tumor prediction map is created for this patch. (8) This map is then used to select the spectrum from the pixel that most likely corresponds to the ground-truth label, which is used to (9) train the classification algorithm. Reproduced with permission, courtesy of Jong et al.^7^

For instance, Kho et al.^96^ employed HSI in breast-conserving surgery by acquiring diffuse reflectance images (900 to 1700 nm) of fresh breast specimens from 18 patients [Fig. 6(a)]. The spectra from various tissue types, including invasive carcinoma (IC), DCIS, adipose tissue, connective tissue, and healthy glandular ducts, were used to train a linear SVM classifier. Samples were divided into two datasets: the first comprised tissue slices obtained after gross sectioning of the resected specimens, annotated and registered with histopathological data; the second dataset contained lumpectomy specimens, imaged across six planes to mimic the resection surfaces of the lesion, as shown in Fig. 6(a). The SVM classifier was trained using the slice dataset and then tested on both datasets, yielding classification accuracies of 93% for IC, 84% for DCIS, 70% for connective tissue, and 99% for adipose tissue. The most significant differences in normalized reflectance spectra occurred around the absorption bands of dominant chromophores in the SWIR, such as water and collagen, which were key for differentiating connective tissue from malignant tissue. However, this approach only utilized spectral data without considering spatial relationships, leading to inaccuracies at tissue transition zones, which are key for margin assessment. In the lumpectomy specimen dataset, each resection side was imaged within 20 s and analyzed in 40 s, suggesting that HSI could outperform frozen section analysis and touch preparation cytology in terms of turnaround time. Moreover, the classification accuracy of IC and DCIS was higher than ultrasound and specimen radiography, approaching the accuracy of frozen section analysis and touch preparation cytology. In a follow-up study, Kho et al.^97^ improved performance using two hyperspectral cameras covering a broadband spectrum (from 450 to 1650 nm) instead of relying on individual visible or near-infrared cameras. They also implemented a spectral–spatial classification algorithm based on a modified U-Net architecture, achieving superior tumor classification performances (sensitivity: 98% and specificity: 99%), even in tissue transition zones. Jong et al.^98^ addressed an important limitation anticipated by Kho et al., demonstrating that a classification algorithm developed for tissue slices performs poorly when applied to lumpectomy specimen surfaces. Although lumpectomy specimens closely resemble tumor tissue during resection, developing a classification algorithm directly from the lumpectomy dataset is challenging because histopathological margin assessment only covers a small fraction of the specimen, leading to insufficiently labeled training data. To overcome this, the researchers used domain adaptation, a transfer learning technique. They developed a spectral - spatial CNN trained on the tissue slice dataset (source domain) and then fine tuned it on the lumpectomy resection surfaces dataset (target domain) to enhance prediction accuracy. They also introduced a partial differential equation loss function to handle label uncertainty, and instead of assigning a single predicted label per pixel, the pixel classification output was delivered in tissue percentages. The proposed method achieved sensitivity, specificity, and accuracy of 91%, 100%, and 96%, respectively.

A fundamental concept in the supervised classification of hyperspectral images is the quality of the ground-truth labels used during training. A typical pipeline for tissue label assignment is shown in Fig. 6(c). In this study by Jong et al.,^7^ hyperspectral images were first acquired, and reference markers (ink) were placed on the specimens to facilitate the association among a patch of pixels in the HSI with the corresponding area on the digitized histopathology slide. This process, known as ground-truth label assignment, can be performed in different ways. For example, if the ink-marked region corresponds to tumor tissue, a “tumor” label can be assigned to either the central pixel’s spectrum or the averaged spectra of all pixels in the patch. These methods, however, are prone to inaccuracies. During pathological processing, it is nearly impossible to perfectly align the center of the tissue slide with the central pixel in the HSI patch due to mechanical distortions. Moreover, a patch offer contains multiple tissue types, making the average spectrum unrepresentative of any single type. An innovative label assignment solution was proposed by Jong et al.^7^ using hyperspectral unmixing, a technique used to decompose each pixel’s signal into its elementary spectral signatures. A tumor probability map is computed from the unmixing process, where each pixel spectrum has a given probability of being positive for the tumor class. The tumor histopathological label is then assigned to the pixel with the highest tumor probability within each patch, reducing the risk of mislabeling. When comparing the normalized diffuse reflectance spectra generated by the three presented label assignment strategies [Fig. 6(b)], hyperspectral unmixing approach had the overall best results with a sensitivity of 94%, specificity of 85%, and accuracy of 87%. In addition, the time of acquisition and analysis of the entire resection surface of the breast lumpectomy specimen was below 10 min (less than 1.5 min per resection side).

##### *In vivo* (reflectance): an example from brain surgery

All *in vivo* clinical studies included in this review focused exclusively on neurosurgery, involving both adult and pediatric patients. This focus may be linked to the significant collaborative European research initiative, HypErspectraL Imaging Cancer Detection (HELiCoiD, 2016 to 2021), which advanced the use of HSI for brain tumor segmentation during surgery. The HELiCoiD project developed a standalone hyperspectral imager and explored various statistical, machine learning, and deep learning strategies to classify tissues.

Puustinen et al.,^10^ Leon et al.,^39^ and Kotwal et al.^138^ recently reviewed several *in vivo* HSI studies in neurosurgery. Key points highlighted by the authors include the following: (i) the studies were non-randomized and mainly comparatives, focusing on different HSI data analysis methods, cameras, or imaging modalities; (ii) inconsistency in study designs and limited datasets restricted performance comparisons to a qualitative level; (iii) different hyperspectral setups were developed for neurosurgery, ranging from standalone systems to cameras integrated into the operating microscope, with the latter offering better integration into the surgical workflow field;^75^^,^^76^ and (iv) the HELiCoiD project released the first open *in vivo* hyperspectral brain database, which includes over 300,000 labeled spectral signatures from 36 images of 22 patients.^99^^–^^101^

To illustrate the impact of such resources, an extended version of the HELiCoiD database allowed the development and validation of a neurosurgical intraoperative HSI system by Leon et al.^39^ This dataset included 62 hyperspectral images from 34 patients affected by a wide range of brain tumors, including primary tumors, from low- to high-grade (WHO grades I to IV), as well as secondary lesions from breast, lung, and kidney cancers. The study first involved the exposure of brain tissue for hyperspectral image acquisition of the cortical surface. Given that image acquisition could extend up to 60 s, additional images were captured during lesion resection exclusively when feasible within the surgical workflow - i.e., if imaging did not introduce significant delays or compromise patient safety. This remains a significant technological limitation, impeding the development of large intraoperative HSI databases due to potential increases in surgical time. Preprocessing of the hyperspectral data corrected for uneven brain surfaces, illumination inconsistencies, spectral noise, and sensor dark current. In addition, data decimation was applied to reduce computational costs. A preliminary evaluation using a paired two-sided Wilcoxon rank sum test (with a 5% significance level) demonstrated significant spectral distinctions between tumor and normal tissue, blood vessels, primary and secondary tumors, and high- and low-grade tumors, as well as among tumor stages. Notably, tumor tissue showed a prominent absorbance contribution from deoxygenated hemoglobin, absent in normal tissue, reflecting the hypoxic tumor environment.

**Fig. 7:**
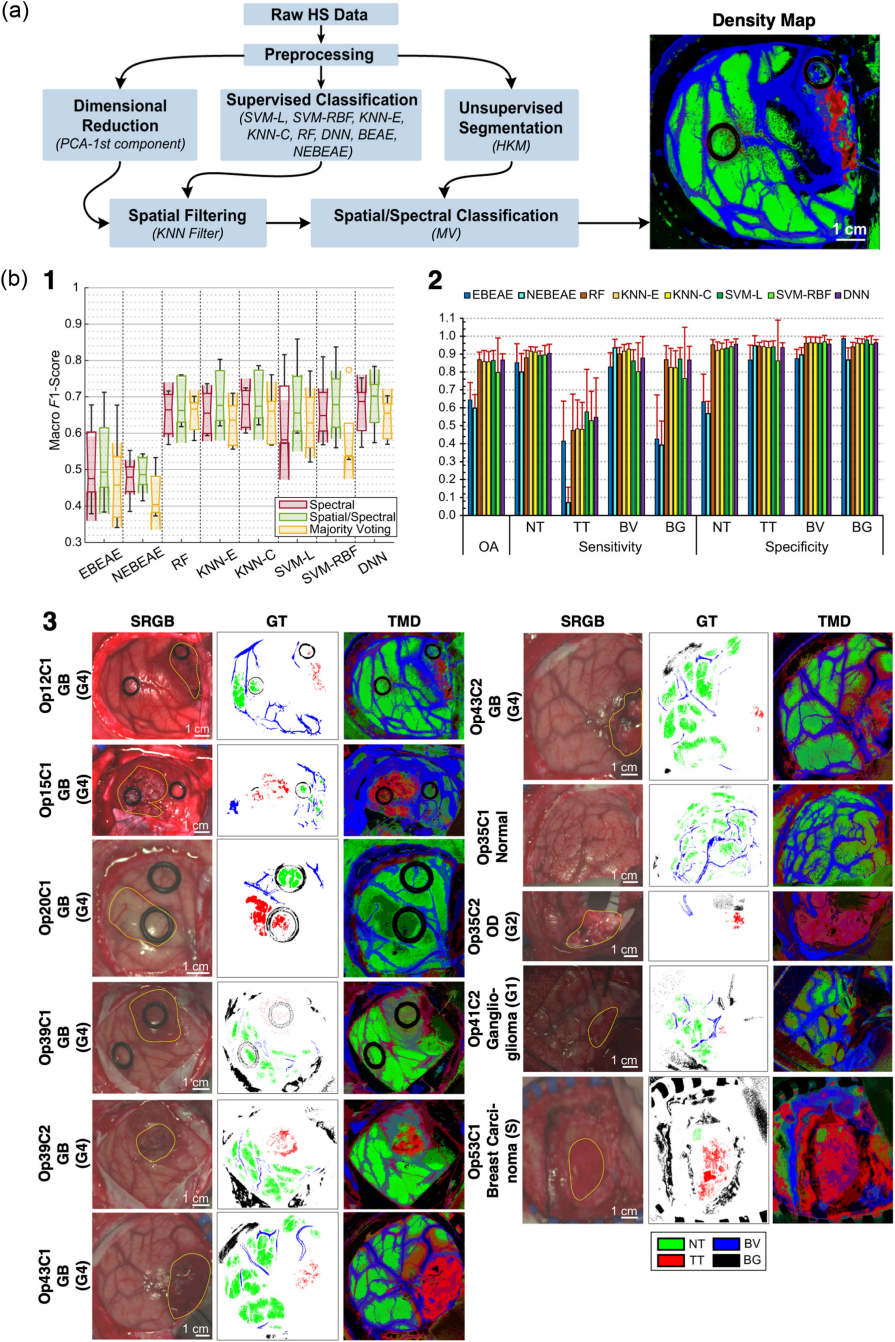
(a) Patient/image flow scheme of this work and data partition. n, number of HS images; m, number of patients. (b) Proposed processing framework to generate the density maps for intraoperative pathology-assisted surgery. Reproduced with permission, courtesy of Leon et al.^39^ (b) (1) Boxplots of the macro F1-score of the test set using the eight different classifiers at the three different stages. In the plot, the center line, the box limits, and the whiskers represent the median, the upper and lower quartiles, and the 1.5× interquartile range, respectively. Two medians are significantly different at the 5% significance level if their intervals (shaded color areas) do not overlap. (2) Average overall accuracy (OA), sensitivity, and specificity results of the test set from the fivefolds using the spatial/spectral approach (error bars represent the standard deviation). (3) Examples of synthetic red, green, blue images (SRGB) images, ground-truth (GT) maps, and three maximum density (TMD) maps from different tumor types (based on the deep neural network (DNN) as a supervised algorithm using the optimal hyperparameters). Reproduced with permission, courtesy of Leon et al.^39^

The authors proposed a sophisticated algorithmic pipeline [Fig. 7(a)] to integrate spatial information into the classification process because preliminary attempts based solely on spectral information provided many false positives. Pre-processed data were first subjected to dimensionality reduction and supervised classification, to then be further processed through a spatial filter (K-nearest neighbors), introducing spatial information into the predictions. Finally, majority voting is used to combine the results from the spectral-spatial supervised classification and the unsupervised segmentation. The authors reported promising tissue class prediction maps [Fig. 7(b)], with the deep neural network achieving the highest median macro F1-score of 70.2±6.3%. Classification inaccuracies were observed in images affected by poor focus or blood contamination in the FOV. Notably, blood contamination is a documented cause of false-positive results in algorithmic tumor margin assessment,^39^ possibly owing to hemoglobin absorption. Given that blood in the FOV is almost inevitable during intraoperative HSI scans, developing strategies for better handling its presence is crucial. This could involve a more comprehensive understanding of blood’s spectral behavior or the agnostic training of more robust algorithms with expanded datasets.

Leon et al.^39^ emphasized improving algorithm interpretability through the utilization of the local interpretable model-agnostic explanations (LIME) algorithm. This aspect was frequently overlooked in the majority of reviewed studies, despite being a primary priority in medicine for enhancing the transparency of predictions and thereby increasing machine learning acceptance in the clinical field. Furthermore, integrating an interpretability step into the algorithmic pipeline can also inform designers about the most informative wavelengths contributing to classification decisions. Indeed, LIME enabled Leon et al. to identify the absorbance peaks of hemoglobin and deoxygenated hemoglobin as crucial wavelengths for tissue classification. The recurring spectral evidence of hypoxia in tumor tissue^102^^–^^104^ warrants special attention, a feature often overlooked or only partially considered by many classification algorithms. Given the promising results from spectral–spatial classification, investigating the integration of a full oxygenation map into predictive algorithms, rather than relying solely on pixel-wise hemoglobin spectral intensity, represents a valuable research direction. This approach is motivated by the potential for tumor-specific hypoxia patterns to provide highly discriminative information for classification. Naturally, these hypotheses require rigorous verification.

**Fig. 8:**
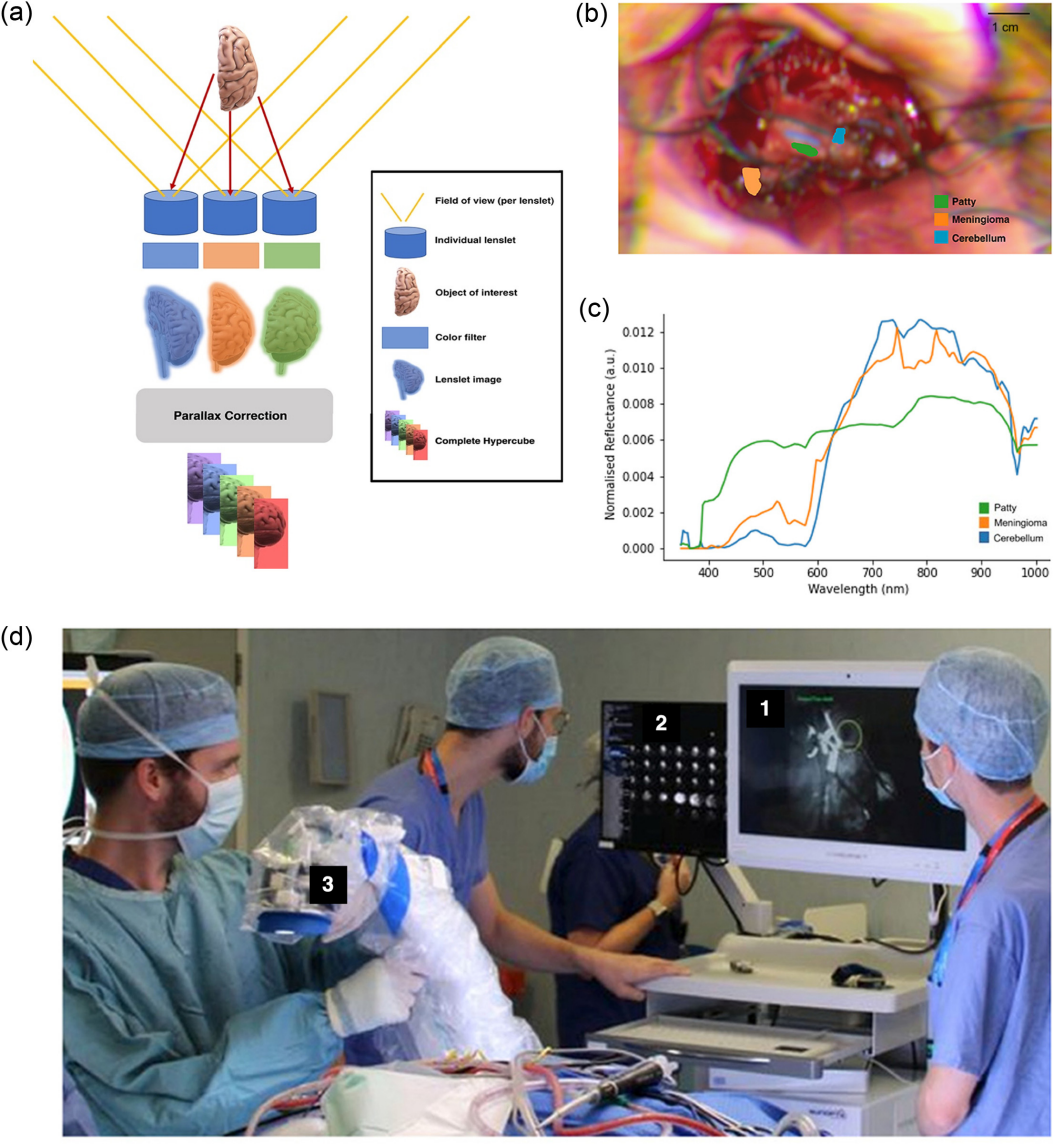
(a) Micro-array of lenslets permits light from a single object to pass through the filters at different angles, creating different spatial and spectral perspectives of the same object. Reproduced with permission, courtesy of MacCormac et al.^76^ (b) Annotated regions of interest on sRGB image reconstructed from HSI data. Computed distance to target: 27 cm. Reproduced with permission, courtesy of MacCormac et al.^76^ (c) L1-normalized spectral curves of annotated structures as shown in panel (b). Reproduced with permission, courtesy of MacCormac et al.^76^ (d) Intra-operative use of lightfield HSI system, draped with Leica 221-88H surgical microscope drape to maintain sterility. (1) Low-resolution “viewfinder” mode. (2) All lenslet images. (3) Draped lightfield HSI system. Reproduced with permission, courtesy of MacCormac et al.^76^

In a separate study, MacCormac et al.^76^ developed a handheld system that employs a novel HSI acquisition method called lightfield hyperspectral imaging, which enables real-time acquisitions while maintaining high spectral resolution. The system design [Figs. 8(a) and 8(d)] incorporates a microarray of lenslets combined with either specific spectral band-pass filters or a single, large, continuously variable spectral filter. These lenslets capture images from multiple angles, which are computationally combined to generate a complete hypercube. The camera used (Cubert Ultris × 50) extracted 155 spectral bands (350 to 1000 nm) using the 66 lenslets images. The system was developed following the surgical device framework outlined by the idea, development, exploration, assessment, and long-term follow-up (IDEAL) collaboration,^105^^,^^106^ encompassing both a preclinical phase (IDEAL-0) for device development and a clinical phase (IDEAL-1) for validation during a posterior fossa meningioma resection. System performance was initially assessed using a Macbeth ColorChecker with known sRGB/CIEXYZ values and reference gold-standard spectral measurements, yielding satisfactory results. The system was then validated on a cadaveric porcine brain, successfully discriminating vessels and cortical brain spectra. Finally, a single-patient validation was conducted during a posterior fossa meningioma resection. Hyperspectral images were acquired under optimal conditions, ensuring the tumor was not obscured by blood or cerebrospinal fluid. These images were then annotated at the pixel level into (i) patty, (ii) meningioma, or (iii) cerebellum using ImFusion Labels software [Fig. 8(b)]. The analysis revealed clear spectral differences, particularly in the 400- to 600-nm wavelength range, where hemoglobin is the dominant chromophore [Fig. 8(c)].

Kifle et al.^75^ applied HSI in a cohort of pediatric patients, integrating a snapshot hyperspectral camera into the neurosurgical microscope to collect a total of 139 RGB, 279 visible, and 85 infrared *in vivo* hyperspectral datasets of pathological brain matter (epileptic or malignant neoplasm). Although the tumor segmentation by a random forest achieved high specificity (99.6%), the average Intersection over Union, which quantifies the overlap between predicted and ground-truth tumor areas, was substantially lower (10%). This finding highlights the need for larger datasets and further algorithm refinement. For the future, the authors plan to integrate surface images with deeper acquisitions from a hyperspectral endoscope, which will address “blind spots” inaccessible to external cameras.

As the final point on the persistent challenge of limited *in vivo* training data, it is important to recognize that although preclinical and *ex vivo* datasets offer some compensation, additional promising strategies warrant exploration. For instance, generative artificial intelligence models present an emerging method for augmenting limited medical imaging datasets by synthesizing highly realistic and diverse images that mimic clinical scenarios.^107^^,^^108^ Although research in remote sensing has demonstrated the ability of such algorithms to generate accurate hyperspectral images,^91^^,^^109^^–^^111^ their direct applicability to the medical domain and capacity to generate reliably annotated HSI datasets remains to be fully determined. Separately, Clancy et al.^1^ reviewed similar generative machine learning methods aimed at estimating tissue spectral properties from regular RGB images. Given the acute data scarcity in pediatric cohorts, addressing the generalizability of *in vivo* adult hyperspectral data for training pediatric classification algorithms is crucial. Although age-specific histologies may intrinsically exhibit distinct spectral profiles, exploring commonalities in the surgical field for which adult training data could prove beneficial remains important.

### 3.4 Fluorescence Analysis

HSI can quantify and characterize the fluorescence spectrum of endogenous fluorophores [e.g., reduced nicotinamide adenine dinucleotide (NADH), lipofuscin, and flavin] or exogenously administered fluorescent contrast agents such as ICG, 5-ALA, and targeted fluorophores.

#### 3.4.1 Preclinical studies on fluorescence analysis

**Fig. 9:**
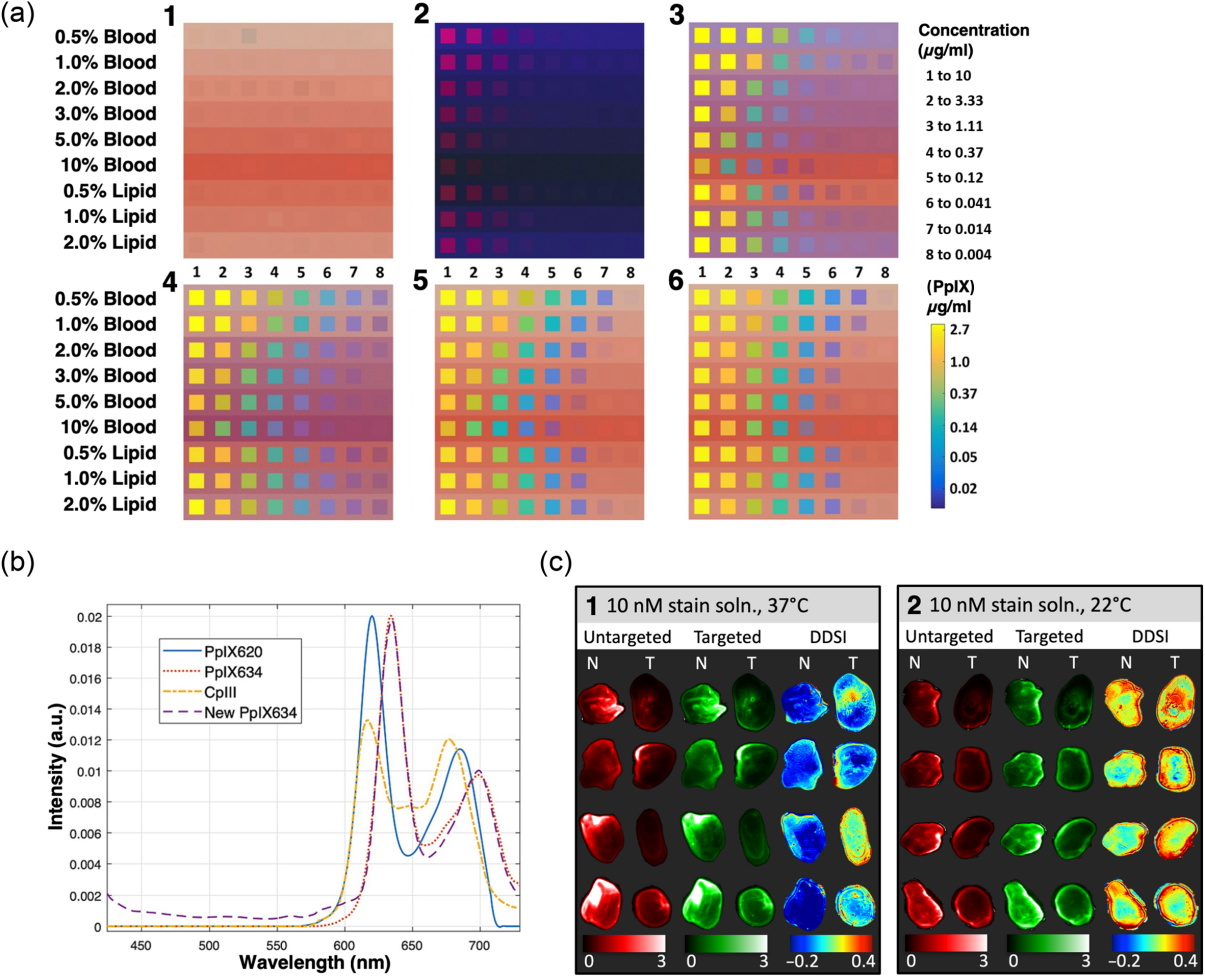
(a) Panels of composite images constructed from experimentally measured phantom data with different processing of PpIX emissions. Within each panel, composite images represent eight PpIX concentrations (left to right, see upper legend far right) mixed with increasing blood volume fraction, constant 1.5% lipid volume fraction (LVF), increasing LVF, and constant 2% blood volume fraction (top to bottom). The background in each row represents a liquid phantom containing no PpIX. Small variations in the color of each phantom are due primarily to minute differences in blood concentration. (1) Reflectance image; pixels were sampled from the white-light images captured by the built-in Zeiss camera. (2) Visible fluorescence image; pixels were sampled from Zeiss fluorescence images. (3) Integrated fluorescence image; integrated fluorescence intensities were converted to concentration units (see lower legend far right) based on assumed phantom optical properties and overlaid onto the reflectance image in (1) using a transparency based on integrated intensity. (4) Spectrally fit image; concentration estimates (SF PpIX) based on the same assumed phantom optical properties were overlaid onto the reflectance image in (1) using a transparency based on concentration. (5) Confidence ratio filtered image; concentration estimates (SF PpIX) were overlaid onto the reflectance image in (1) using a transparency based on the confidence ratio (CR). (6) Optical property corrected image; concentration estimates (C PpIX) based on optical properties specific to each individual phantom were overlaid onto the reflectance image in (1) using a transparency based on the CR. Reproduced with permission, courtesy of Bravo et al.^3^ (b) Porphyrin basis spectra for unmixing tests. CpIII and the new PpIX634 are based on pig brain phantoms. The old PpIX spectra (labeled PpIX620 and PpIX634) are from Black et al.^34^ The new and old PpIX634 match very well, so the new PpIX634 and the CpIII should be directly applicable to our existing human data. Reproduced with permission, courtesy of Suero Molina et al.^90^ (c) Representative specimen images after topical staining/washing for all four staining conditions investigated. Columns labeled “untargeted” and “targeted” represent images of the tissue after pixel-by-pixel spectral fitting of the hyperspectral data and normalization to the calibration volume and thus show the fluorescence of the targeted and untargeted quantum dot-labeled antibody complexes (QDAC) channels. The DDSI column shows the processed DDSI images. “N” and “T” refer to normal and tumor tissue, respectively. Each panel presents four specimen samples from each staining condition: (1) Incubation in a 10-nM stain solution at 37°C and (2) incubation in a 10-nM stain solution at 22°C. Reproduced with permission, courtesy of Meng et al.^88^

Among preclinical fluorescence studies, neurosurgery-related research was the most represented (n=4) (Table 2), primarily focusing on the use of 5-ALA, which leads to the selective accumulation of PpIX in glioma tissue.^66^ For instance, Bravo et al.^3^ employed wide-field HSI to analyze liquid phantoms with varying concentrations of PpIX, lipid, and blood [Fig. 9(a)]. They applied spectral fitting algorithms to generate PpIX fluorescence maps, differentiating fluorescence from background signals. This approach improved PpIX sensitivity, lowering the camera’s detection limit from 0.37  μg/mL (naked eye) to a range spanning from 0.014 to 0.041  μg/mL. The enhanced sensitivity helps reduce inter-operator variability and increases the detection of tumors with low fluorescence uptake, particularly in low-density tumor regions. For instance, a minimum tumor cell density of 20% to 30% in malignant glioma is typically required to detect visible fluorescence.^112^^,^^113^

Lehtonen et al.^33^ found that PpIX concentrations in low-grade gliomas and glioblastoma infiltration zones were below the naked-eye detection threshold (0.6 to 1.8  μmol/L in their liquid phantom tests) but were detectable using HSI at concentrations as low as 0.03 to 0.15  μmol/L.

Walke et al.^89^ tested different phantom designs to optimize HSI system calibration for glioma imaging. Initially, they used liquid phantoms containing known PpIX concentrations and variable optical properties (e.g., intralipid for scattering and yellow dye for absorption). However, these phantoms had limitations: (i) a lower pH compared with gliomas, which affected PpIX fluorescence intensity; (ii) provided only one PpIX photostate (PpIX634); and (iii) contained only PpIX as the fluorophore, unlike biological tissues, which contain multiple fluorophores and autofluorescence sources. To address these issues, Walke et al.^89^ proposed using pig brain homogenates for system calibration and validation, as they better replicate the optical properties of biological tissue, including endogenous fluorophores and multiple photostats. However, this method still does not accurately reproduce the physiological pH range of tumor samples (pH 6 to 8), affecting the PpIX fluorescence measurements. For example, they found that PpIX levels were six times higher at pH 8.8 compared with pH 5.1 in reference tissue homogenates. This innovative phantom design, combining animal homogenates with standard models, enhances HSI algorithms by accounting for varying optical properties, biochemical microenvironments, and multiple autofluorescence sources, crucial for real-time surgical fluorescence characterization.

Literature evidence shows that PpIX fluorescence emission consists of two main peaks at 634 nm (PpIX634) and 620 nm (PpIX620).^114^ Building on this, Suero Molina et al.^90^ employed HSI to investigate the molecular origins of the 620-nm peak, hypothesizing it might represent a different fluorophore rather than a second PpIX photostate. This assumption is significant as other porphyrin precursors in the heme synthesis pathway, such as coproporphyrin III (CpIII) and uroporphyrin (Up) or alternative autofluorescence sources, may artificially enhance the 620-nm peak. Using a phantom design similar to Walke et al.,^89^ the authors combined cerebrum tissue homogenates with PpIX or CpIII stock solutions. HSI acquisitions revealed that CpIII shares spectral characteristics with PpIX620 [Fig. 9(b)], suggesting that the 620-nm peak may originate from other molecular species. These studies demonstrate that HSI, coupled with accurate spectral unmixing algorithms, can better characterize the spectral properties of tumor tissue and contrast agents. However, caution is necessary when quantifying fluorescence to minimize false negatives and positives. This approach offers valuable insights into tumor biology and holds potential for improving tumor segmentation algorithms for surgical resection.

In a proof-of-concept study, De Landro et al.^87^ used HSI to detect a fluorescently labeled antibody targeting high mobility group protein B1, a marker overexpressed in various neoplastic cells associated with tumorigenesis and inflammation-related immunosuppression. The authors found a significant correlation between relative absorbance at 640 nm and antibody concentration, demonstrating the potential of HSI for detecting fluorescent antibodies in image-guided surgery and preoperative diagnosis during endoscopic examinations. Meng et al.^88^ further expanded on this application by employing HSI for dual-probe difference specimen imaging (DDSI) on freshly excised specimens of normal tissue and human tumor xenografts overexpressing the HER2 marker [Fig. 9(c)]. DDSI utilized two quantum dot-labeled antibody complexes: one targeting the HER2 tumor biomarker and the other serving as an untargeted isotype control. A shared excitation source illuminated both probes, generating spectrally distinct fluorescence emissions captured by the hyperspectral camera. This approach enabled tissue segmentation based on label binding specificity, facilitating differential imaging of tumor and normal tissue. However, the diagnostic performance of DDSI was temperature-dependent: at body temperature (37°C), diagnostic performance was robust with an area under the curve (AUC) greater than 0.81. At room temperature (22°C), the AUC dropped to 0.61, highlighting the importance of maintaining physiological conditions during imaging. Notably, increasing the concentration of the staining solution did not enhance diagnostic performance, suggesting that factors other than concentration influence performance. These findings emphasize the need for optimized tissue processing time and workflow efficiency to facilitate the translation of DDSI into rapid intraoperative diagnostics of excised specimens.

#### 3.4.2 Clinical studies on fluorescence analysis

The use of HSI for fluorescence analysis has been extensively validated in a cohort of ∼550 patients, demonstrating its clinical potential. Remarkably, all clinical fluorescence studies in this review were related to neurosurgery, likely due to the critical need to resolve the fluorescence signal of 5-ALA from overlapping autofluorescence and other spectral artifacts inherent to the fluorophore emission spectrum.^115^

**Fig. 10:**
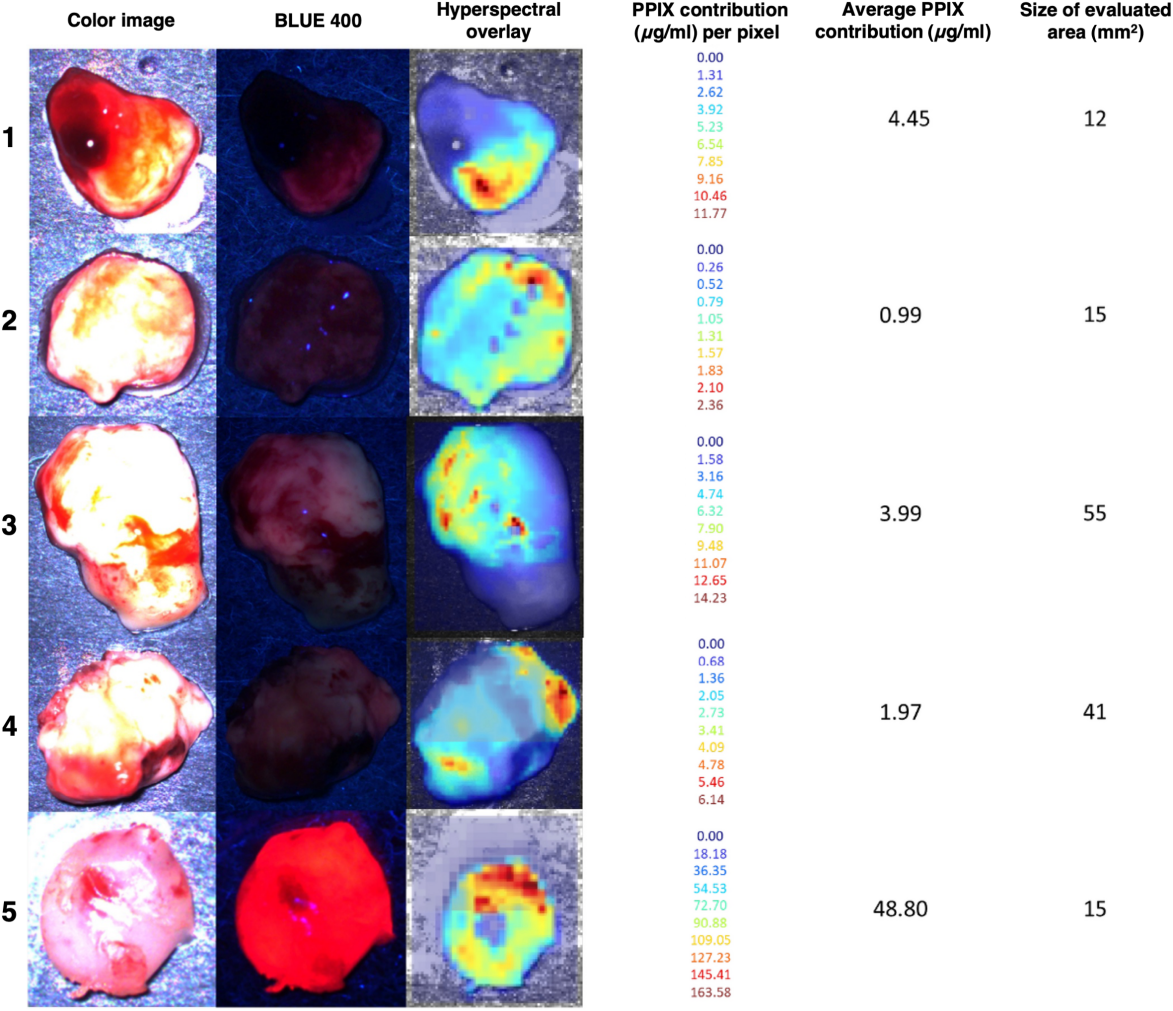
Hyperspectral images of biopsies. Average PpIX contribution (μg/ml) and the evaluated tissue area size are given. (1) Infiltration zone (IZ) biopsy with no visible fluorescence in the microscope; hyperspectral measurement visualizes PpIX fluorescence and residual blood. (2) Solid tumor (ST) biopsy of small size with pixels of high reflectance and underestimated PpIX contribution. Microscope fluorescence rating was “none,” whereas in hyperspectral imaging, weak fluorescence was visible (2-BLUE 400). (3) and (4) Reactively altered brain tissue (RABT) samples of middle size, fluorescence quality “weak” in the microscope. Both showed heterogeneous PpIX distribution. (5) Tiny ST biopsy showing strong fluorescence in the surgical microscope and an artificially high PpIX contribution in the hyperspectral measurement of up to 164  μg/ml. Reproduced with permission, courtesy of Walke et al.^89^

Walke et al.^89^ evaluated 240 high-grade glioma biopsies to compare PpIX fluorescence visualized through either the KINEVO 900 surgical microscope (SM) (equipped with the BLUE 400 filter) or a wide-field hyperspectral camera (Fig. 10). For each biopsy, fluorescence emission was captured between 421 and 730 nm in 3-nm increments, with an excitation wavelength of 405 nm. The acquisition of each sample took ∼3  min. PpIX fluorescence contributions were calculated at every pixel, and these values were then averaged to generate a single PpIX concentration for the entire biopsy. Only pixels with contributions greater than 0.1  μg/mL were included in the average calculation, as this threshold reliably distinguished background signals from actual PpIX fluorescence. Such a threshold was particularly useful for analyzing heterogeneous biopsies, which may have fluorescent hotspots surrounded by regions of low signal that could be due to low tumor cell density (observed in 17 of 96 biopsies), low proliferation rates (noted in 70 of 96 biopsies), or healthy tissue. The study found that HSI outperformed the surgical microscope for biopsy diagnosis when compared with neuropathological assessments as the gold standard (AUC [HSI]=0.845±0.024 versus AUC [SM]=0.710±0.035). Furthermore, the diagnostic cutoff for PpIX concentration was lower with HSI (0.75  μg/mL) than with the surgical microscope (0.99  μg/mL) (Fig. 10). The study highlighted that the fluorescence assessment with the surgical microscope is highly subjective, whereas HSI provides objective and quantifiable analysis.

As introduced in the preclinical section of fluorescence studies, Suero Molina et al.^90^ hypothesized that 5-ALA-induced PpIX fluorescence visualization could be influenced by other autofluorescence sources, potentially amplifying the secondary peak at 620 nm and leading to overestimation of fluorescence. After validating this hypothesis in preclinical experiments, the authors analyzed over 200,000 spectra from ∼600 tumor biopsies collected from 130 patients. Using spectral unmixing techniques, the authors identified the elementary sources of the spectral signatures, including PpIX634, PpIX620, lipofuscin, flavin, and NADH. They then investigated the correlation of these components with various tumor characteristics, including isocitrate dehydrogenase (IDH) mutation status (mutated versus wildtype), fluorescence visibility during surgery (graded as none, weak, or strong), 5-ALA dosage (single or double), and tumor margin type (solid tumor, infiltration zone, or reactive altered brain tissue). The results indicated that PpIX634 was significantly more informative in all classifications compared with PpIX620. For instance, PpIX634 strongly correlated with isocitrate dehydrogenase mutation status and fluorescence visibility, whereas PpIX620 remained relatively constant across the different classes. These findings suggest that device optimization should prioritize certain spectral signals, such as PpIX634, to improve diagnostic accuracy. In addition, the study revealed that different tumor histologies exhibited varying levels of autofluorescence, further demonstrating that HSI provides a more quantitative assessment than non-spectroscopic methods.

Intraoperative real-time imaging faces distinct challenges compared with controlled laboratory biopsy imaging. Critical requirements for its successful implementation include rapid fluorescence data processing and robust calibration methodologies to mitigate the effects of dynamic biochemical microenvironments, autofluorescence, and endogenous absorbers such as blood.^89^^,^^116^ To address these challenges, the authors proposed combining tissue homogenates with standard phantoms for system calibration to better account for the target tissue’s endogenous spectral properties.^89^ We believe this solution could potentially be useful in other surgical specialties employing fluorescent dyes, especially those emitting in the visible range (such as fluorescein) that are most affected by endogenous chromophores, whereas this issue is less relevant for NIR probes owing to the less significant autofluorescence in this spectral region. These hypotheses, however, necessitate verification via high-quality research studies, similar to those presented herein for neurosurgery. Finally, Suero Molina’s research group is actively advancing fluorescence data processing pipelines and intraoperative tumor classification through the employment of innovative machine learning strategies. These algorithms, interpreting fluorophore abundance, offer the surgeon the potential for rapid and enhanced fluorescence visualization.^77^^,^^117^^,^^118^

Hyperspectral fluorescence analysis was also explored in pediatric brain tumor resection by Schwake et al.^74^ Eleven children (aged 1 to 16 years) with various brain tumors underwent surgery after receiving oral 5-ALA administration (4 h before the procedure). Consistent with earlier case reports and studies, higher PpIX concentrations were detected in the four malignant astrocytomas (WHO grade III). In contrast, no fluorescence was observed in the two grade II astrocytomas or three medulloblastomas. Fluorescence was also detected in both cases of pilocytic astrocytomas. No significant side effects were reported, aside from a mild increase in liver transaminases, indicating that 5-ALA was generally well-tolerated. Nonetheless, the study underscores the need for prospective controlled trials to establish the feasibility of 5-ALA use in pediatric brain surgery.

### 3.5 Tumor Characterization (Grade/Type Prediction)

HSI holds promise for predicting tumor grade or type intra- or post-operatively. This is typically performed within a multi-class classification framework, where each class represents a distinct tumor grade or histology. Studies of this kind have been conducted on both fresh surgical specimens and H&E-stained tissue slides, though a lack of *in vivo* studies was observed. It must be noted that the body of research in this area is much more limited compared with tumor segmentation studies.

For what concerns fresh surgical specimens, Edwards et al.^119^ utilized HSI combined with multiparametric radiomic features to predict tumor aggressiveness in 72 fresh *ex vivo* surgical specimens from 44 patients with papillary thyroid cancer. Hyperspectral images were processed using the PyRadiomics package, from which 67 radiomic features (e.g., shape-based, gray-level dependence matrix) were selected. By testing various combinations of feature selection algorithms and classification methods, the study achieved a maximum accuracy of 83.3% for predicting tumor aggressiveness. Among the features, gray-level dependence matrix variance was the most influential in distinguishing aggressive from non-aggressive tumor tissue. Follow-up work on the same dataset by Leitch et al.^120^ incorporated an HSI pixel dilation to highlight the tumor-normal tissue interface, which is particularly indicative of tumor aggressiveness. In this analysis, the shape feature “least axis length” emerged as the most predictive for classification.

In the setting of post-operative diagnosis, Liu et al.^121^ used a microscope with a built-in pushbroom hyperspectral camera to scan H&E-stained pathological sections of gastric cancer from 30 patients. The study aims to automatically classify cancer grades (low, intermediate, and high) and healthy tissue using a shallow residual network, yielding an average classification accuracy of 91.44%, surpassing RGB image analysis by 13.87%.

Hyperspectral fluorescence imaging has also been reported to aid in distinguishing tumor type and grade in recent studies by Black and Suero Molina et al.,^77^^,^^115^^,^^118^ as summarized in a dedicated book chapter.^117^ One such study by the same group^77^ involved analyzing hyperspectral fluorescence data from biopsy specimens, acquired after 5-ALA administration, to classify (i) tumor types (e.g., glioblastoma and meningioma), (ii) WHO grades, (iii) margin types, and (iv) IDH-mutant versus IDH-wildtype glioma. Random forest and multilayer perceptron classifiers, applied to unmixed spectra, achieved average test accuracies of 84 to 87%, 96.1%, 86%, and 91%. This research demonstrates how varying fluorophore abundances correlate with different tumor grades and histologic profiles. Although this example pertained to brain samples, a similar behavior would be expected in other organ systems.

The studies presented in this section employ diverse methodologies and sample types, which preclude direct performance comparisons. Nevertheless, they collectively highlight feasible avenues for advancing HSI’s predictive ability in estimating tumor grade and type, diagnostic information fundamental to surgical decision-making and prognosis.

### 3.6 Tissue Identification

HSI has the potential to differentiate the intrinsic spectral characteristics of various tissue types, providing real-time guidance to surgeons in identifying critical structures during surgical procedures. Although the primary focus of this review is tumor analysis, HSI has also been extensively studied for its ability to identify non-pathological tissues, aiding the identification of essential anatomical structures, such as the nerves and blood vessels, which are crucial for surgical navigation and preservation.

For example, Puustinen et al.^10^ conducted a multi-tissue classification study, in which hyperspectral data from a single patient were processed using various machine learning algorithms for pixel-wise classification into six tissue types: (i) blood, (ii) compact bone, (iii) dura mater internal leaf, (iv) gray matter with pia, (v) superficial vein, and (vi) glioma. The two best-performing classifiers were the light gradient boosting machine, which achieved the highest accuracy of 98.3% with a classification time of 4.68 s, and the convolutional neural network, which achieved a classification time of 2.79 s and demonstrated high accuracy, although the exact value was not reported.

### 3.7 Augmented Reality

Huang et al.^80^ developed an HSI-augmented reality (AR) integrated system capable of projecting tissue segmentation - distinguishing tumors, blood vessels, and healthy brain tissue - directly onto the surgeon’s FOV. Validated using a brain tumor phantom, the system integrates an HSI device that captures spectral data across 150 wavelength bands, with a shallow neural network for pixel-wise tissue classification. The resulting classification is then displayed on an augmented reality visor (Microsoft HoloLens) worn by the surgeon. The optical phantom used for validation consisted of colorless gelatin to represent healthy brain tissue, yellow-dyed gelatin to simulate tumors, and porcine blood to mimic blood vessels. Although the system produced accurate segmentation maps, an important technological limitation highlighted in the study was the lengthy processing time, which was too slow for real-time surgical use. Other limitations in the AR headset included insufficient memory, stability, and image capture quality.^80^

**Fig. 11:**
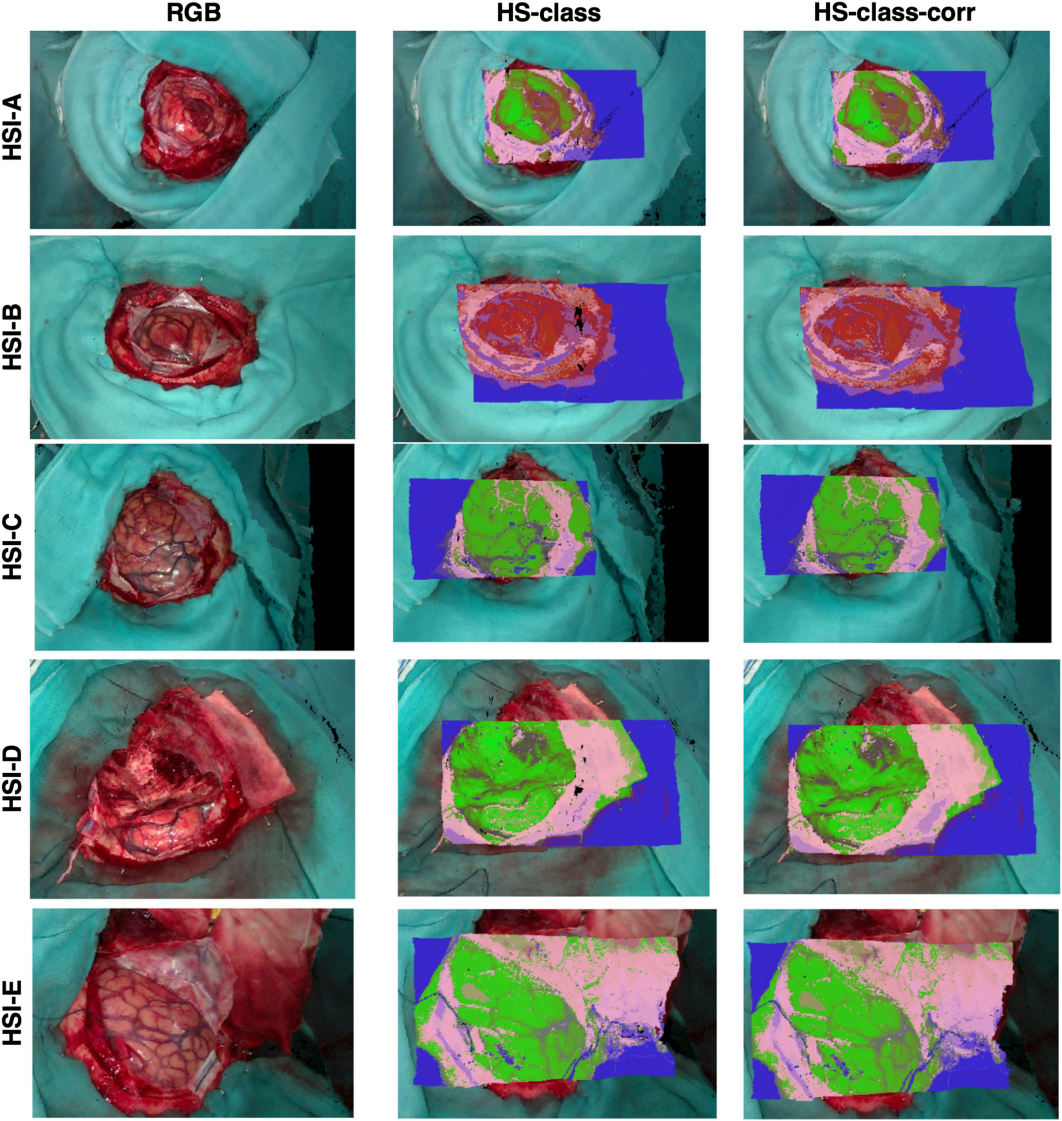
Point cloud results in real operations. Each row refers to a different tumor operation, whereas each column refers to a type of visualization. From left to right: RGB point cloud, RGB point cloud with HS classification information, and RGB point cloud with HS classification information and depth correction. (For the interpretation of the references to color in this figure legend, the reader is referred to the web version of this article.) Reproduced with permission, courtesy of Sancho et al.^122^

Sancho et al.^122^ introduced SLIMBRAIN, an intraoperative AR system leveraging HSI to present a freely navigable three-dimensional (3D) point cloud video of the tumor prediction map. The system setup included a light source, a snapshot hyperspectral camera, a light detection and ranging (LiDAR) system with a Time-of-Flight camera, an RGB camera, and a graphics processing unit - accelerated processing station to reduce computation delays and enable real-time visualization. As shown in Fig. 11, the final output allows the neurosurgeon to interactively navigate the 3D model in real-time, whereas the data are continuously recorded. The system achieved a maximum frame rate of ∼21  fps (33 ms for LiDAR capture and 14 ms for processing), though the hyperspectral camera limited the frame rate to 14 fps due to exposure time requirements. Tumor prediction was performed using an SVM classifier, achieving a global AUC and tumor-specific AUC of ∼95% when compared with the ground truth annotated by neurosurgeons.

## 4 Discussion and Conclusion

This review examined preclinical and clinical studies exploring HSI for image-guided surgery in tumor resection. HSI can assist tumor resection in two key ways: (i) real-time intraoperative imaging of the surgical site (*in vivo*)^39^ and (ii) imaging of resected specimens for rapid optical biopsy (*ex vivo*).^45^^,^^121^ This study highlights the broad applicability of HSI across diverse tissue types and anatomical regions in both adult and pediatric populations, with neurosurgery emerging as the most extensively studied field to date. HSI remains in its early stages of development, as evidenced by the predominance of proof-of-concept and feasibility studies, which often provide qualitative rather than quantitative performance assessments.^39^ Consequently, the superiority of HSI over intraoperative ultrasound and other emerging real-time intraoperative imaging modalities - such as confocal laser endomicroscopy, optical coherence tomography, and photoacoustic imaging - has yet to be definitively established.

HSI captures a broader range of wavelengths than multispectral imaging, allowing for a more comprehensive spectral characterization of the sample.^79^^,^^123^ However, the large dataset it generates presents both advantages and challenges. Although HSI facilitates sophisticated predictions,^39^^,^^90^^,^^94^^,^^98^^,^^122^ its increased acquisition time and computational load hinder its real-time application.^89^^,^^124^ To address such limitations, many studies have reduced the number of analyzed spectral bands, demonstrating that this reduction does not compromise the classification performance of HSI systems.^35^^,^^93^ The selective acquisition of relevant bands could streamline hardware design, reduce processing time, and improve usability in operating room environments.^93^^,^^125^ In addition, post-acquisition algorithms can be employed to optimize band selection. An example of such an approach is the genetic algorithm-based optimization proposed by Martinez et al.^93^

The most common HSI applications in surgical oncology involve tissue reflectance and fluorescence. Although HSI can provide detailed spectral data without the use of exogenous contrast agents, it can also enhance sensitivity for fluorescence probes, allowing a lower detection threshold, enabling more sensitive detection compared with traditional surgical visualizations (e.g., surgical microscopes), and potentially reducing morbidity associated with contrast administration.^3^^,^^33^^,^^34^^,^^77^ The high spectral resolution of HSI was also found to be advantageous in precisely characterizing the spectrum of administered fluorescent agents, such as 5-ALA, and in effectively distinguishing them from autofluorescence signals arising from heterogeneous biological tissues.^90^ For example, HSI has been shown to lower the detection threshold for PpIX fluorescence, providing a quantitative advantage over subjective visual assessments.^34^^,^^77^^,^^90^

A key strength of HSI in tumor resection is its ability to segment tissues based on distinct spectral signatures. Although the exact reasons remain unclear, tumor spectra are generally distinct from those of healthy tissues.^7^^,^^75^^,^^101^^,^^126^ This phenomenon is due to the intrinsic chemical characteristics of tumor tissue, which are reflected in the measured spectra. One important example is the strong deoxyhemoglobin absorption associated with the tumor’s hypoxic environment.^39^^,^^85^ A recent study by Giannoni et al.^125^ introduced two “spectral biomarkers” for distinguishing high-grade from low-grade gliomas. The first biomarker reflects changes in the oxidation state of cytochrome c oxidase, indicative of the metabolic rewiring in tumor tissue.^127^^,^^128^ The second biomarker relates to the lipid spectral profile of tumor tissues, which reflects structural and metabolic modifications in glioblastoma, leading to substantial lipid storage.^129^^,^^130^ New spectral biomarkers are continually emerging in the literature, highlighting the need to explore the causal relationships behind these associations to enhance the predictive process. A valid approach involves analyzing the contributions of multiple spectral biomarkers (chromophores and fluorophores) isolated from measured spectra using sophisticated spectral unmixing algorithms. This task has been facilitated by the availability of application-specific spectral libraries, such as the one recently published by Black et al.,^115^ which includes endmember spectra for PpIX (620 and 634 nm photostates), NADH, FAD, flavins, lipofuscin, melanin, elastin, and collagen. Such a comprehensive study of tumor spectral sources may enable the establishment of more robust spectral associations while accounting for the significant tumor heterogeneity. Recent advances have demonstrated the potential of machine learning to automate the spectral unmixing process.^118^ However, further work is needed to make the endmember spectra estimation process more interpretable, which is essential for ethical acceptance.

Pre-processing of HSI data remains critical to improve signal quality before the generation of classification maps through machine learning algorithms. The information conveyed by these classification maps varies depending on the prediction task. For instance, binary classification maps distinguish between tumor and healthy tissue, whereas multi-class maps differentiate among multiple tumor histologic types or grades.^119^^,^^121^^,^^131^ However, the current pipelines for generating these maps have limited domain knowledge of light–tissue interactions and tumor biology, relying heavily on the algorithm itself as a “black box.”^39^ Although this approach simplifies the classification conceptually, it can result in inconsistent and unpredictable performance.^39^ For instance, errors that are easily recognized by a scientist reviewing the maps, such as misclassifying blood as tumor tissue, are frequently overlooked by algorithms. A more detailed spectral characterization of tissues, including blood, in controlled experimental settings could provide valuable insights to improve and educate these algorithms. Spectral - spatial tumor classification outperformed purely spectral classification in most studies,^8^^,^^39^^,^^132^^,^^133^ often achieving sensitivity and specificity rates exceeding 90%. However, these results should be interpreted with caution, as the validation datasets in the reviewed studies were often too small to support reliable conclusions. Expanding these datasets and incorporating more robust domain knowledge into algorithm design could enhance the accuracy and reliability of HSI-based tumor segmentation.

One of the most critical aspects of developing and reliably implementing HSI guidance in surgical settings is establishing a robust methodology for ground-truth training and validation of tissue predictions generated from hyperspectral data.^7^^,^^96^ In this context, ground-truth data refer to tissue samples precisely labeled by expert pathologists using gold-standard techniques, such as histopathology, or by the surgeon’s visual assessment, particularly for *in vivo* data. These data serve as a reference standard for training and validating machine learning models. There is no universally “best” ground-truth sample; instead, the protocol should align with the type of tissue being imaged (e.g., *in vivo* or *ex vivo*) and the intended classification output. Significant spectral differences exist between *in vivo* and *ex vivo* tissue samples, likely due to reduced blood flow, lower oxygen levels, and decreased water content in *ex vivo* samples. Although fresh *ex vivo* ground-truth validation samples may be sufficient for validating the HSI of resected surgical specimens, they may not adequately represent *in vivo* surgical images. Alternatively, *in vitro* or *in vivo* tumor models can also serve as ground-truth sources. A promising yet underexplored example is the tumor-on-chip platform, which closely mimics the complexity of the tumor microenvironment^134^^,^^135^ while providing a highly controlled experimental setting suitable for detailed spectral characterization experiments. Finally, an intriguing area of exploration for HSI ground-truth labeling involves fusing MRI data - a well-established imaging modality for tumor staging - with hyperspectral images, as demonstrated by the pioneering work of Villa et al.^136^^,^^137^ It remains to be determined whether MRI data fusion can improve label accuracy, especially in challenging cases such as infiltrative tumors,^77^^,^^138^ and help automate the labeling process.^138^

Several initiatives have significantly contributed to addressing the need for accessible ground-truth data. Fabelo et al.^39^^,^^101^^,^^139^^,^^140^ launched the first public database comprising three datasets of *in vivo* hyperspectral brain and tumor images, accessible through a dedicated online portal. Ortega et al. published a dataset containing 469 annotated hyperspectral images derived from histological slides of glioblastoma obtained from 13 patients.^141^ Similarly, Giannoni et al.^125^^,^^142^ released a publicly available dataset of 14 fresh surgical biopsies of glioma tissue imaged using a custom-built HSI system. As part of the SLIMBRAIN initiative^122^ (discussed in Sec. 3.7), two additional datasets were published,^136^^,^^137^^,^^143^^,^^144^ one of which also included corresponding MRI scans registered to the hyperspectral images.^136^^,^^137^ The SLIMBRAIN research group also developed a high-quality, dedicated online portal featuring extensive learning resources and detailed instructions to facilitate navigation of the dataset.^122^^,^^123^

Publicly available data from other surgical oncology specialties and fluorescence studies are notably scarce, as the reviewed studies restricted data access. Puustinen et al.^145^ highlighted a critical gap in available reports, pointing out the frequent absence of essential clinical information, such as precise anatomical annotations, locational details, and corresponding MRI scans. They therefore proposed a systematic framework for a neuro-microsurgical hyperspectral database,^145^ which could be extended to other medical specialties. This framework should include comprehensive elements such as patient information, raw hyperspectral data, RGB reconstructions, imaging parameters, manual annotations, pre-operative MRI scans, regions of interest, calibration standards, and labeled classes.^138^^,^^145^ Building on these recommendations, whenever possible, databases should also include samples from both healthy and tumor tissues, along with detailed descriptions of sample preparation methods and reagents used. The creation of additional freely accessible HSI databases would allow for a better representation of tumor tissues’ diverse microstructural and metabolic characteristics. Such resources would facilitate more effective data fusion, support the optimization and benchmarking of machine learning algorithms, and ultimately advance the field of HSI in surgical oncology.

A key limitation of optical imaging methods, including HSI, is their restricted penetration depth into biological tissues.^44^^,^^146^^,^^147^ Computational approaches, such as Monte Carlo simulations, can model and quantify HSI penetration depth across different spectral bands by simulating photon transport within a 3D model with arbitrary optical properties. Generally, penetration depth is limited to a few millimeters (≤10  mm) and varies depending on the imaging wavelengths and the tissue’s optical properties. A recent study by Giannoni et al.^125^ estimated penetration depths using the Monte Carlo method, finding values of 0.5 to 0.75 mm for visible light and up to nearly 5 mm for near-infrared light. One promising approach to enhance the imaging depth limitation is to combine HSI with complementary real-time intraoperative imaging modalities capable of deeper acquisitions, such as photoacoustic imaging^148^^,^^149^ or ultrasound.^149^^–^^151^

There are only a few commercially available fully integrated HSI surgical systems. One example is the TIVITA^®^ 2.0 from Diaspective Vision GmbH, which has been used in some of the reviewed studies. This device provides information on the relative oxygen saturation of blood in the microcirculatory system, with penetration depths ranging from ∼1 to 6 mm into superficial and deeper tissue layers. It also generates indices related to water and fat distribution in tissues. Another commercial system, the Quest Spectrum^®^ (Quest Medical Imaging B.V.), enables intraoperative hyperspectral fluorescence imaging. However, neither system is currently optimized for the complex tumor segmentation tasks required in surgical oncology. In addition to hardware limitations, there is a notable lack of dedicated and validated software for hyperspectral data analysis, which further hinders the advancement of human clinical trials. Addressing these gaps will require collaborative efforts among companies (through joint ventures), hospitals, academia, policymakers, and funders. At the current stage of HSI technology development, estimating the total cost of implementation is difficult, as most systems are still experimental. In the future, a deeper understanding of the spectral characteristics of tissues, particularly tumors, could guide the development of more cost-effective systems and software solutions.^125^ HSI holds significant promise for improving the time and cost-efficiency of surgical workflows by reducing uncertainty around tumor boundaries in real-time. This could lead to safer surgeries with fewer complications, reduced recurrence rates, and shorter hospital stays. If such clinical benefits are validated, the initial investment in HSI could be offset by long-term savings through enhanced surgical precision and overall healthcare efficiency, supporting its feasibility for broader clinical adoption.

In conclusion, HSI shows great potential as a guidance technology in surgical oncology, offering valuable intraoperative insights for tissue segmentation, fluorescence analysis, optical biopsy, and more. However, HSI is still in its early stages of development, requiring significant advancements in both software and hardware for its full integration into surgical practice. Robust study designs, including randomized controlled trials, are essential to objectively evaluate the effectiveness of HSI-guided tumor resection compared with other intraoperative imaging modalities. Although HSI is unlikely to replace traditional histopathological processing, which remains the gold standard for diagnosis, it can serve as a powerful supplementary tool to support real-time surgical decision-making. As clinical guidelines increasingly emphasize histological type for determining resection extent, multi-class classification tasks are likely to become essential, moving beyond the current binary tumor segmentation approaches. The integration of optimized hardware, advanced machine learning algorithms, and precise biological characterization of tissue spectra will be crucial for addressing current limitations and facilitating the broader clinical adoption of HSI in the near future.

## Supplementary Material

10.1117/1.JBO.30.S2. S23909.s01

## Data Availability

All data in support of the findings of this paper are available within the article or as Supplementary Material.
