# Supplementary material for: Hyperspectral imaging for tumor resection guidance in surgery: a systematic review of preclinical and clinical studies
Source: J Biomed Opt. 2025 Aug 6;30(Suppl 2):S23909. doi: 10.1117/1.JBO.30.S2.S23909 (PMC12327654; doi:10.1117/1.JBO.30.S2.S23909)
Supplement: Supplementary file 1 [file JBO_030_S23909_SD001.pdf]

Supplementary Data

Supplementary Table 1. Search String Used for the Three Respective Databases (Medline, Embase, Web of Science)

The first search was conducted on 09/10/2023 (dd/mm/yyyy) and updated on 10/05/2024.

Medline (PubMed): 571 articles

((("hyperspectral imaging"[MeSH Terms] OR (("hyperspectral"[Text Word] OR "hyper-spectral"[Text Word]) AND ("imag*"[Text Word] OR "microscop*"[Text Word] OR "camera*"[Text Word])))) AND (("surgical procedures, operative"[MeSH Terms] OR "surgery"[MeSH Subheading] OR "endoscopy"[MeSH Terms] OR "laparoscopy"[MeSH Terms] OR "surg*"[Text Word] OR "intraoperat*"[Text Word] OR "intra-operat*"[Text Word] OR "operat*"[Text Word] OR "endoscop*"[Text Word] OR "laparoscop*"[Text Word]))) AND (("neoplasms"[MeSH Terms] OR "oncolog*"[Text Word] OR "tumor*"[Text Word] OR "tumour*"[Text Word] OR "neoplasm*"[Text Word] OR "cancer*"[Text Word] OR "tumor microenvironment"[MeSH Terms] OR "TME"[Text Word] OR (("tissues"[MeSH Terms] OR "tissue*"[Text Word]) AND ("characteri*"[Text Word] OR "diagnosis"[MeSH Terms] OR "diagnos*"[Text Word] OR "differentia*"[Text Word] OR "oxygeni*"[Text Word])) OR "cell differentiation"[MeSH Terms] OR "humans"[MeSH Terms] OR "human*"[Text Word] OR "cell*"[Text Word] OR "hypoxia"[MeSH Terms] OR "hypoxia"[Text Word] OR "cells"[MeSH Terms] OR "lymphocytes"[MeSH Terms] OR "lymphocy*"[Text Word] OR "immun*"[Text Word] OR "molecul*"[Text Word] OR "function*"[Text Word] OR "blood vessels"[MeSH Terms] OR "blood"[MeSH Terms] OR "blood"[MeSH Subheading] OR "blood"[Text Word] OR "hematology"[MeSH Terms] OR "hematoma"[MeSH Terms] OR "hemorrhage"[MeSH Terms] OR "hemato*"[Text Word] OR "haemato*"[Text Word] OR "hemorrhag*"[Text Word] OR "haemorrhag*"[Text Word] OR "vascular*"[Text Word] OR "vessel*"[Text Word] OR "angiogenesis"[Text Word] OR "metabolism"[MeSH Terms] OR "metabolism"[MeSH Subheading] OR "metaboli*"[Text Word] OR "chemi*"[Text Word] OR "drug*"[Text Word] OR "therapeutics"[MeSH Terms] OR "therapy"[MeSH Subheading] OR "therapeutic*"[Text Word] OR "artificial intelligence"[MeSH Terms] OR "artificial intelligence"[Text Word] OR "machine learning"[MeSH Terms] OR "machine learning"[Text Word] OR "deep learning"[MeSH Terms] OR "deep learning"[Text Word] OR "algorithms"[MeSH Terms] OR "algorithm*"[Text Word] OR "models, statistical"[MeSH Terms] OR "statistic*"[Text Word] OR "spectrum analysis"[MeSH Terms] OR "spectrum analysis"[Text Word] OR "spectral analysis"[Text Word]))

Time limits applied: January 1st, 2014 – April 30th, 2024

Embase: 427 articles

1 hyperspectral imaging/ 1480
2 ((hyperspectral or hyper-spectral) and (imag* or microscop* or camera*)).ti,ab,kw. 5288
3 1 or 2 5479
4 exp surgery/ 6534792
5 surgery.fs. 2570144
6 exp endoscopy/ 832987
7 exp laparoscopy/ 207554
8 surg*.ti,ab,kw. 3473296
9 intraoperat*.ti,ab,kw. 257909
10 intra-operat*.ti,ab,kw. 38077
11 operat*.ti,ab,kw. 2051066
12 endoscop*.ti,ab,kw.429032
13 laparoscop*.ti,ab,kw. 261337
14 4 or 5 or 6 or 7 or 8 or 9 or 10 or 11 or 12 or 13 8960323
15 exp neoplasm/ 6198690
16 tumor microenvironment/ 119990
17 oncolog*.ti,ab,kw. 406653
18 cancer*.ti,ab,kw. 3411491
19 tumo?r*.ti,ab,kw. 3028622
20 neoplasm*.ti,ab,kw.272536
21 cancer*.ti,ab,kw. 3411491
22 TME.ti,ab,kw. 24663
23 (exp tissues/ or tissue*.ti,ab,kw.) and (exp diagnosis/ or (characteri* or diagnos* or differentia* or oxygeni*).ti,ab,kw.) 3628167
24 exp human/ 27867465
25 human.ti,ab,kw. 3789095
26 exp cell differentiation/ 736184
27 exp cells/ 8455432
28 cell*.ti,ab,kw. 8143090

29 exp hypoxia/ 171502
30 hypoxia.ti,ab,kw. 205729
31 exp lymphocyte/ 1112674
32 lymphocy*.ti,ab,kw. 610480
33 immun*.ti,ab,kw. 3997905
34 molecul*.ti,ab,kw. 3054516
35 function*.ti,ab,kw. 6000891
36 exp blood vessel/ 1437154
37 exp blood/ 3085250
38 blood.ti,ab,kw. 3327114
39 exp hematology/ 104454
40 exp hematoma/ 140372
41 exp bleeding/ 1276932
42 h?emato*.ti,ab,kw. 788136
43 h?emorrhag*.ti,ab,kw. 474449
44 vascular*.ti,ab,kw. 1087566
45 vessel*.ti,ab,kw. 557700
46 exp angiogenesis/ 162276
47 angiogenesis.ti,ab,kw. 174287
48 exp metabolism/ 7118104
49 metaboli*.ti,ab,kw. 2004377
50 chemi*.ti,ab,kw. 1470623
51 drug*.ti,ab,kw. 2973233
52 exp therapy/ 11305957
53 therapy.fs. 1875555
54 therapeutic*.ti,ab,kw. 2130428
55 exp artificial intelligence/ 102356
56 artificial intelligence.ti,ab,kw. 58033
57 exp machine learning/ 474965
58 machine learning.ti,ab,kw. 130288
59 deep learning/ 56492
60 deep learning.ti,ab,kw. 72599
61 exp algorithm/ 636615
62 algorithm*.ti,ab,kw. 491666
63 exp statistical model/ 733969
64 statistic*.ti,ab,kw. 2213639
65 spectrum analysis.ti,ab,kw. 4861
66 spectral analysis.ti,ab,kw. 25636
67 15 or 16 or 17 or 18 or 19 or 20 or 21 or 22 or 23 or 24 or 25 or 26 or 27 or 28 or 29 or 30 or 31 or 32 or 33 or 34 or 35 or 36 or 37 or 38 or 39 or 40 or 41 or 42 or 43 or 44 or 45 or 46 or 47 or 48 or 49 or 50 or 51 or 52 or 53 or 54 or 55 or 56 or 57 or 58 or 59 or 60 or 61 or 62 or 63 or 64 or 65 or 66 40081592
68 3 and 14 and 67 876
69 limit 68 to dc=20140101-20240430

Time limits applied: January 1st, 2014 – April 30th, 2024

Web of Science: 1,830 articles

(hyperspectral OR "hyper-spectral") AND (imag* OR microscop* OR camera*) AND (surg* OR intraoperat* OR "intra-operat*" OR operat* OR endoscop* OR laparoscop*) AND (oncolog* OR tumor* OR tumour* OR neoplasm* OR cancer* OR TME OR (tissue* AND (characteri* OR diagnos* OR differentia* OR oxygeni*)) OR human* OR cell* OR hypoxia or lymphocy* OR immun* OR molecul* OR function* OR blood OR hemato* OR haemato* OR hemorrhag* OR haemorrhag* OR vascular* OR vessel* OR angiogenesis OR metaboli* OR chemi* OR drug* OR therapeutic* OR "artificial intelligence" OR "machine learning" OR "deep learning" OR algorithm* OR statistic* OR "spectrum analysis" OR "spectral analysis")(Topic)

Time limits applied: January 1st, 2014 – April 30th, 2024

Supplementary Table 2. Inclusion and Exclusion Criteria Used to Select Studies for the Review

Inclusion Criteria	Exclusion Criteria
- Any peer-reviewed, published interventional or observational study reporting the use of hyperspectral imaging (HSI) to characterize tumor tissue and guide its surgical resection, or pre-clinical studies aimed at translating HSI for intra-operative use in tumor resection guidance<br>- Full-text available	- Not written in English<br>- Commentary, review, case report, technical report, letter to the editor, or abstract-only<br>- Duplicate<br>- Incomplete information on experimental conditions<br>- Raman spectroscopy/hyperspectral imaging

Supplementary Table 3. Summary dataset of the included studies

Rank	Author last name, first name. Year. "Article Title." Journal Name Volume, Issue number (Publication date): Page range. DOI or URL	Technology-specific																	Clinical							
		Intervention (Application)	Manufacturer	Model	Sensor	Acquisition Method	Wavelength Range	Field of View (FOV)	Dispersive Element	Number of Spectral Bands	Spectral Resolution	Spatial Resolution	Temporal Resolution	Data Cube Acquisition Time	Light Source	Data Analysis Method	Performance Metrics	Setting (intra/extra-operative)	Sample Type/ District	In-vivo/Ex-vivo	Number of Patients	Preparation Modality (fixed, fresh, etc.)	Type of Acquired Signal	Population	Intervention (Application)
1	Aboughaleb, I. H., et al. (2020). "Hyperspectral imaging for diagnosis and detection of ex-vivo breast cancer." Photodiagnosis Photodyn Ther **31**: 101922.	Tumor segmentation	Surface Optics	SOC710	Silicon-based CCD	Line-scanning (pushbroom)	400 - 1000 nm (optimized: 420 - 620 nm)	10 degrees	Na	128 (optimized: 6)	4.69 nm	(max / nominal) 1040 / 520 Px	(max / nominal) 100 / 30 fps per binning option)	(max / nominal) 6.96 / 23.2 seconds/cube (696 x 520 x 128 cube dimensions)	Broadband (348 - 800 nm)	K-means clustering	Sensitivity: 95 %; Specificity: 96 %	Extra-operative	Breast	Ex-vivo	10	Fresh	Reflectance	Adult	Tumor segmentation
2	Aref, M. H., et al. (2023). "Emerging technology for intraoperative margin assessment and post-operative tissue diagnosis for breast-conserving surgery." Photodiagnosis Photodyn Ther **42**: 103507.	Tumor segmentation	Surface Optics	SOC710	Silicon-based CCD	Line-scanning (pushbroom)	400 - 1000 nm	Na	Na	128 (optimized: 4)	4.69 nm	(max / nominal) 1040 / 520 Px	(max / nominal) 100 / 30 fps per binning option)	(max / nominal) 6.96 / 23.2 seconds/cube (696 x 520 x 128 cube dimensions)	Broadband (390 - 980 nm)	K-means clustering + Contour delineation	Sensitivity: 98.95 %; Specificity: 98.44 %	Extra-operative	Breast	Ex-vivo	30	Formalin-fixed, Paraffin-embedded: Two groups: Stained (H&E), Unstained	Reflectance	Adult	Tumor segmentation
3	Baltussen, E. J. M., et al. (2019). "Hyperspectral imaging for tissue classification, a way toward smart laparoscopic colorectal surgery." J Biomed Opt **24**(1): 1-9.	Tumor segmentation; Tissue identification	SPECIM	**VIS:** PFD-CL-65-V10E; **NIR:** VLNIR CL-350-N17E	**VIS:** CMOS; **NIR:** InGaAs	Line-scanning (pushbroom)	**VIS:** 400 - 1000 nm; **NIR:** 900 - 1700 nm **(optimized VIS:** 450 - 950 nm; **optimized NIR:** 970 - 1600 nm)	Na	Na	(optimized: 13)	**VIS:** 3 nm; **NIR:** 5 nm	**VIS:** 1 x 1312 Px; **NIR:** 1 x 320 Px	Na	**VIS:** 30 s; **NIR:** 20 s	Broadband halogen (unspecified)	K-means clustering + Quadratic classifier + Linear support vector machine classifier	**Combined VIS and NIR:** Sensitivity: 93 %; Specificity: 90 %	Extra-operative	Colorectal	Ex-vivo	54	Fresh	Reflectance	Adult	Tumor segmentation; Tissue identification
4	Black, D., et al. (2021). "Characterization of autofluorescence and quantitative protoporphyrin IX biomarkers for optical spectroscopy-guided glioma surgery." Sci Rep **11**(1): 20009.	Fluorescence quantification; Autofluorescence quantification	Na	Na	CMOS	Na	420 - 730 nm	Na	Liquid crystal tunable filter	Na	Na	Na	Na	Na	Broadband LED (unspecified)	Spectral unmixing	Na	Extra-operative	Brain	Ex-vivo	128	Fresh	Endogenous Fluorescence; Exogenous Fluorescence (5-ALA)	Adult	Fluorescence quantification; Autofluorescence quantification
5	Bravo, J. J., et al. (2017). "Hyperspectral data processing improves PpIX contrast during fluorescence guided surgery of human brain tumors." Sci Rep **7**(1): 9455.	Fluorescence quantification	Experimental	Experimental	CMOS	Na	**Reflectance:** 440 - 720 nm; **Fluorescence:** 600 - 720 nm	Na	Liquid crystal tunable filter	Na	**Reflectance:** 4 nm; **Fluorescence:** 3 nm	Na	Na	**Reflectance:** 7 s; **Fluorescence:** 12 s	Xenon (white light); Blue light fluorescence module/filter [Zeiss BLUE 400]	Spectral fitting	Na	Intra-operative	Brain; Phantom	In-vivo; Phantom	Na	Fresh; Phantom	Exogenous Fluorescence (5-ALA)	Adult	Fluorescence quantification
6	Brouwer de Koning, S. G., et al. (2020). "Toward assessment of resection margins using hyperspectral diffuse reflection imaging (400-1,700nm) during tongue cancer surgery." Lasers Surg Med **52**(6): 496-502.	Tumor segmentation	SPECIM	**VIS:** PFD-CL-65-V10E; **NIR:** VLNIR CL-350-N17E	**VIS:** CMOS; **NIR:** InGaAs	Line-scanning (pushbroom)	**VIS:** 400 - 950 nm; **NIR:** 950 - 1700 nm	Na	Na	Na	**VIS:** 3 nm; **NIR:** 5 nm	**VIS:** 0.16 mm/Px; **NIR:** 0.5 mm/Px	Na	Na	Broadband halogen (unspecified)	Feedforward neural network	**VIS:** Sensitivity: 84 %; Specificity: 77 %; **NIR:** Sensitivity: 80 %; Specificity: 77 %; **combined:** Sensitivity: 83 %; Specificity: 76 %;	Extra-operative	Tongue	Ex-vivo	14	Fresh	Reflectance	Adult	Tumor segmentation
7	Collins, T., et al. (2022). "Automatic optical biopsy for colorectal cancer using hyperspectral imaging and artificial neural networks." Surg Endosc **36**(11): 8549-8559.	Tumor segmentation	Diaspective Vision GmbH	TIVITA	CMOS	Line-scanning (pushbroom)	500 - 1000 nm	Na	Na	100	5 nm	640 x 476 Px	100 fps	6.4 s	Broadband halogen (incoherent white light ~ 400 - 700)	Convolutional neural network	Sensitivity: 87 %; Specificity: 90 %	Intra-operative	Colorectal	Ex-vivo	34	Fresh	Reflectance	Adult	Tumor segmentation
8	Collins, T., et al. (2021). "Automatic Recognition of Colon and Esophagogastric Cancer with Machine Learning and Hyperspectral Imaging." Diagnostics (Basel) **11**(10).	Tumor segmentation	Diaspective Vision GmbH	TIVITA	CMOS	Line-scanning (pushbroom)	500 - 1000 nm	Na	Na	100	5 nm	640 x 476 Px	100 fps	~ 10 s	Broadband (unspecified)	Random forest classifier; Logistic regression; Support vector machine (linear and radial basis function kernel); Multi-layer perceptron; Three-dimensional convolutional neural network	Mean ROC-AUC [3D-CNN]: 93 %	Intra-operative	Gastro-esophageal junction; Colon; Colorectal	Ex-vivo	22	Fresh	Reflectance	Adult	Tumor segmentation
9	De Landro, M., et al. (2023). "In Vitro Antibody Quantification with Hyperspectral Imaging in a Large Field of View for Clinical Applications." Bioengineering (Basel) **10**(3).	Fluorescence quantification	Diaspective Vision GmbH	TIVITA	CMOS	Line-scanning (pushbroom)	500 - 995 nm	Na	Na	100	5 nm	640 x 476 Px	100 fps	6 s	Broadband halogen (unspecified)	Na	Na	Extra-operative	Na	In-vitro	0	Fluoresecently labeled antibodies dissolved in PBS	Exogenous Fluorescence (Alexa Fluor 647 Dye)	Na	Fluorescence quantification
10	Edwards, K., et al. (2021). "Multiparametric Radiomics for Predicting the Aggressiveness of Papillary Thyroid Carcinoma Using Hyperspectral Images." Proc SPIE Int Soc Opt Eng **11597**.	Tumor grade prediction	Cambridge Research & Instrumentation	CRI Maestro	Na	Na	450 - 900 nm	Na	Liquid crystal tunable filter	91	5 nm	25 µm (1040 × 1392 Px)	Na	~ 1 min	Xenon (unspecified)	Quadratic discriminant analysis	Accuracy: 83 %; AUC: 85 %	Extra-operative	Thyroid	Ex-vivo	44	Fresh	Reflectance	Adult	Tumor grade prediction
11	Fabelo, H., et al. (2019). "Deep Learning-Based Framework for In Vivo Identification of Glioblastoma Tumor using Hyperspectral Images of Human Brain." Sensors (Basel) **19**(4).	Tumor segmentation	Headwall Photonics	Hyperspec® VNIR A-Series	Silicon-based CCD	Line-scanning (pushbroom)	400 - 1000 nm	Max: 129 × 230 mm	Na	826	2 - 3 nm	1004 × 826 Px (with a linear displacement transducer: 1004 x 1787 Px)	90 fps	Na	Broadband halogen [quartz tungsten] (400 - 2200 nm)	Main: Two-dimensional convolutional neural network; Deep neural network + Hierarchical K-means clustering + Majority voting; Adjunct: Principal component analysis; **Support vector machine classifier**; K-nearest neighbors	Accuracy: 80 %	Intra-operative	Brain	In-vivo	16 (H)	Fresh	Reflectance	Adult	Tumor segmentation
12	Fabelo, H., et al. (2019). "Surgical Aid Visualization System for Glioblastoma Tumor Identification based on Deep Learning and In-Vivo Hyperspectral Images of Human Patients." Proc SPIE Int Soc Opt Eng **10951**.	Tumor segmentation	Headwall Photonics	Hyperspec® VNIR A-Series	Silicon-based CCD	Line-scanning (pushbroom)	400 - 1000 nm (optimized: 450 - 900 nm)	Na	Na	826 (optimized: 128)	2 - 3 nm	1004 Px	90 fps	Na	Broadband halogen [quartz tungsten] (400 - 2200 nm)	Main: Two-dimensional convolutional neural network; One-dimensional deep neural network + Hierarchical K-means clustering + Majority voting; Adjunct: Principal component analysis + **Support vector machine classifier** + K-nearest neighbors	Accuracy (binary): 95 %; Accuracy (multiclass): 85 %	Intra-operative	Brain	In-vivo	H (16 to be neglected)	Fresh	Reflectance	Adult	Tumor segmentation
13	Fabelo, H., et al. (2018). "An Intraoperative Visualization System Using Hyperspectral Imaging to Aid in Brain Tumor Delineation." Sensors (Basel) **18**(2).	Tumor segmentation	Headwall Photonics	**VNIR:** Hyperspec® VNIR A-Series; **NIR:** Hyperspec® NIR 100/U	**VNIR:** Silicon-based CCD; **NIR:** InGaAs	Line-scanning (pushbroom)	**VNIR:** 400 - 1000 nm; **NIR:** 900 - 1700 nm	Na	Na	**VNIR:** 826; **NIR:** 172	**VNIR:** 2 - 3 nm; **NIR:** 5 nm	**VNIR:** 1004 Px; **NIR:** 320 Px	**VNIR:** 90 fps; **NIR:** 100 fps	**VNIR:** ~ 80 s; **NIR:** ~ 40 s	Broadband halogen [quartz tungsten] (400 - 2200 nm)	Support vector machine classifier; K-nearest neighbors; Hierarchical K-means clustering; Majority voting	Na	Intra-operative	Brain	In-vivo	22 (H)	Fresh	Reflectance	Adult	Tumor segmentation
14	Fabelo, H., et al. (2018). "Spatio-spectral classification of hyperspectral images for brain cancer detection during surgical operations." PLoS One **13**(3): e0193721.	Tumor segmentation	Headwall Photonics	Hyperspec® VNIR A-Series	Silicon-based CCD	Line-scanning (pushbroom)	400 - 1000 nm	Na	Na	826 (optimized: 129)	2 - 3 nm	1004 Px	90 fps	Na	Broadband halogen [quartz tungsten] (400 - 2200 nm)	Support vector machine classifier; K-nearest neighbors; Hierarchical K-means clustering; Majority voting	Na	Intra-operative	Brain	In-vivo	5 (H)	Fresh	Reflectance	Adult	Tumor segmentation
15	Fabelo, H., et al. (2019). "<i>In</i>-<i>Vivo</i> Hyperspectral Human Brain Image Database for Brain Cancer Detection." Ieee Access **7**: 39098-39116.	Tumor segmentation	Headwall Photonics	Hyperspec® VNIR A-Series	Silicon-based CCD	Line-scanning (pushbroom)	400 - 1000 nm	Na	Na	826	2 - 3 nm	1004 Px	90 fps	Na	Broadband halogen [quartz tungsten] (400 - 2200 nm)	Na	Na	Intra-operative	Brain	In-vivo	H (22 to be neglected)	Fresh	Reflectance	Adult	Tumor segmentation
16	Fei, B., et al. (2017). "Label-free hyperspectral imaging and quantification methods for surgical margin assessment of tissue specimens of cancer patients." Annu Int Conf IEEE Eng Med Biol Soc **2017**: 4041-4045.	Tumor segmentation	Perkin Elmer	CRI Maestro	Silicon-based CCD	Na	450 - 900 nm	Na	Liquid crystal tunable filter	Na	5 nm	Na	Na	Na	Xenon (unspecified)	Linear discriminant analysis; Support vector machine	Accuracy: 90 %; Sensitivity: 89 %; Specificity: 91 %	Extra-operative	Head; Neck	Ex-vivo	16	Fresh	Reflectance	Adult	Tumor segmentation
17	Halicek, M., et al. (2020). "Tumor detection of the thyroid and salivary glands using hyperspectral imaging and deep learning." Biomed Opt Express **11**(3): 1383-1400.	Tumor segmentation	Perkin Elmer	CRI Maestro	Silicon-based CCD	Na	450 - 900 nm	Na	Liquid crystal tunable filter	91	5 nm	25 µm (1040 × 1392 Px)	Na	~ 1 min	Xenon (unspecified)	**Thyroid tumor detection:** Inception convolutional neural network; **Salivary gland tumor detection:** Multi-Layer Perceptron	**Thyroid tumor detection:** AUC: 90 % ; Sensitivity: 80 %; Specificity: 79 %; **Salivary gland tumor detection:** AUC: 92 %; Sensitivity: 90 %; Specificity: 79 %	Extra-operative	Thyroid; Salivary gland	Ex-vivo	82	Fresh	Reflectance	Adult	Tumor segmentation
18	Halicek, M., et al. (2019). "Hyperspectral Imaging of Head and Neck Squamous Cell Carcinoma for Cancer Margin Detection in Surgical Specimens from 102 Patients Using Deep Learning." Cancers (Basel) **11**(9).	Tumor segmentation	Perkin Elmer	CRI Maestro	Silicon-based CCD	Na	450 - 900 nm	Na	Liquid crystal tunable filter	91	5 nm	25 µm (1040 × 1392 Px)	Na	~ 1 min	Na	Inception convolutional neural network	AUC: 92 %	Extra-operative	Oral cavity; Tongue; Oropharynx; Hypopharynx; Larynx; Nasal cavity; Maxillary sinus	Ex-vivo	102	Fresh	Reflectance	Adult	Tumor segmentation
19	Halicek, M., et al. (2019). "Hyperspectral imaging for head and neck cancer detection: specular glare and variance of the tumor margin in surgical specimens." J Med Imaging (Bellingham) **6**(3): 035004.	Tumor segmentation	Perkin Elmer	CRI Maestro	Silicon-based CCD	Na	450 - 900 nm (optimized: 490 - 790 nm)	Na	Na	91 (optimized: 61)	5 nm	25 µm (1040 × 1392 Px)	Na	Na	Na	Three-dimensional convolutional neural network; Support vector machine classifier; K-nearest neighbors; Hierarchical K-means clustering	AUC [CNN + HELICoiD (preprocessed HSI)]: 81 %	Extra-operative	Larynx; Pharynx; Tongue; Floor of mouth + Alveolar ridge + Buccal mucosa; Maxillary sinus	Ex-vivo	12	Fresh	Reflectance	Adult	Tumor segmentation
20	Halicek, M., et al. (2019). "Optical biopsy of head and neck cancer using hyperspectral imaging and convolutional neural networks." J Biomed Opt **24**(3): 1-9.	Tumor segmentation; Tissue identification; Tumor type prediction	Perkin Elmer	CRI Maestro	Silicon-based CCD	Na	450 - 900 nm	Na	Liquid crystal tunable filter	91	5 nm	25 µm (1040 × 1392 Px)	Na	Na	Xenon (unspecified)	Two-dimensional inception convolutional neural network; Three-dimensional convolutional neural network	**Normal/SCC:** AUC: 92 %; **Tissue identification:** AUC: 94 %; **Normal/ thyroid carcinoma:** AUC: 95 %; **MTC/MNG:** AUC: 93%; **PTC/MNG:** AUC: 91 %	Extra-operative	Tongue; Larynx; Pharynx; Thyroid; Mandible	Ex-vivo	21	Fresh	Reflectance	Adult	Tumor segmentation; Tissue identification; Tumor type prediction
21	Halicek, M., et al. (2018). "Tumor Margin Classification of Head and Neck Cancer Using Hyperspectral Imaging and Convolutional Neural Networks." Proc SPIE Int Soc Opt Eng **10576**.	Tumor segmentation	Perkin Elmer	CRI Maestro	Silicon-based CCD	Na	450 - 900 nm	Na	Liquid crystal tunable filter	91	5 nm	25 µm (1040 × 1392 Px)	Na	Na	Na	Two-dimensional and three dimensional convolutional neural networks; Two-dimensional and three dimensional inception convolutional neural networks Support vector machine classifier; Linear discriminant analysis; Random forest classifier	**Normal/SCC:** AUC: 86 %; **Normal/thyroid carcinoma:** AUC: 94 %	Extra-operative	Thyroid; Maxillary sinus; Mandibular mucosa + Hard palate + Buccal mucosa; Oropharynx	Ex-vivo	20	Fresh	Reflectance	Adult	Tumor segmentation
22	Halicek, M., et al. (2017). "Deep convolutional neural networks for classifying head and neck cancer using hyperspectral imaging." J Biomed Opt **22**(6): 60503.	Tumor segmentation	Perkin Elmer	CRI Maestro	Silicon-based CCD	Na	450 - 900 nm	Na	Liquid crystal tunable filter	91	5 nm	25 µm (1040 × 1392 Px)	Na	Na	Xenon (unspecified)	Convolutional neural network; Support vector machine classifier; K-nearest neighbors; Logistic regression; Decision tree classifier; Linear discriminant analysis	Accuracy: 80 %; Sensitivity: 81 %; Specificity: 78 %	Extra-operative	Tongue; Larynx; Pharynx; Mandible; Thyroid	Ex-vivo	50	Fresh	Reflectance	Adult	Tumor segmentation
23	Hao, Q. B., et al. (2021). "Fusing Multiple Deep Models for <i>In Vivo</i> Human Brain Hyperspectral Image Classification to Identify Glioblastoma Tumor." Ieee Transactions on Instrumentation and Measurement **70**: 14.	Tumor segmentation	Headwall Photonics	Hyperspec® VNIR A-Series	Silicon-based CCD	Line-scanning (pushbroom)	400 - 1000 nm	Na	Na	826	2 - 3 nm	1004 Px per line	Na	Na	Na	(Spectral phasor analysis and data oversampling) + One-dimensional deep neural network-based spectral HSI classification + Two-dimensional convolutional neural network-based spectral–spatial HSI classification + (Edge-preserving filtering-based classification results fusion and optimization) + Two-dimensional convolutional neural network (spectral-spatial) + Fully convolutional network-based semantic segmentation	**Overall:** Accuracy: 96.69 %; Precision: 92.68 %; Sensitivity: 90.82 %; **Tumor:** Accuracy: 96.34 %; Precision: 87.76 %; Sensitivity: 90.53 %	Intra-operative	Brain	In-vivo	H (22 to be neglected)	Fresh	Reflectance	Adult	Tumor segmentation
24	Huang, J., et al. (2020). "Augmented reality visualization of hyperspectral imaging classifications for image-guided brain tumor phantom resection." Proc SPIE Int Soc Opt Eng **11315**.	Tumor segmentation; Augmented reality guidance	Na	Na	Na	Na	485 - 900 nm	Na	Na	150	2.5 nm	1088 x 2048 Px	Na	~ 40 s	Na	Simple neural network (1 hidden layer)	Na	Extra-operative	Phantom (brain)	Phantom	0	Phantom constituents (agar, bovine gelatin, porcine whole blood, etc.)	Reflectance	Na	Tumor segmentation; Augmented reality guidance
25	Jansen-Winkeln, B., et al. (2021). "Feedforward Artificial Neural Network-Based Colorectal Cancer Detection Using Hyperspectral Imaging: A Step towards Automatic Optical Biopsy." Cancers (Basel) **13**(5).	Tumor segmentation	Diaspective Vision GmbH	TIVITA	CMOS	Line-scanning (pushbroom)	500 - 1000 nm	Na	Na	100	5 nm	0.39 mm/Px (640 x 480 Px)	100 fps	~ 6 s	Broadband (visible and near-infrared)	Four-layer perceptron neural network; Random forest classifier; Support vector machine classifier	Sensitivity: 86 %; Specificity: 95 %	Intra-operative	Colorectal	Ex-vivo	54	Fresh	Reflectance	Adult	Tumor segmentation
26	Jong, L. S., et al. (2022). "Discriminating healthy from tumor tissue in breast lumpectomy specimens using deep learning-based hyperspectral imaging." Biomed Opt Express **13**(5): 2581-2604.	Tumor segmentation	SPECIM	**VIS:** PFD-CL-65-V10E; **NIR:** VLNIR CL-350-N17E	**VIS:** CMOS; **NIR:** InGaAs	Line-scanning (pushbroom)	**VIS:** 400 - 1000 nm (optimized: 450 - 951 nm); **NIR:** 900 - 1700 nm (optimized: 954 - 1650 nm)	Na	Na	**VIS:** 384 (optimized: 318); **NIR:** 256 (optimized: 210)	**VIS:** 3 nm; **NIR:** 5 nm	**VIS:** 0.16 mm/Px (1312 x 384 Px); **NIR:** 0.5 mm/Px (320 x 256 Px); **Combined VIS and NIR:** 0.5 mm/Px (320 x 256 Px)	Na	~ 1 min	Broadband halogen (unspecified)	One-dimensional, three-dimensional and dual-channel convolutional neural networks	Sensitivity [DC-CNN]: 91 %; Specificity [DC-CNN]: 100 %; MCC [DC-CNN]: 92 %; Accuracy [DC-CNN]: 96 %	Extra-operative	Breast	Ex-vivo	163	**Lumpectomy:** Fresh; **Slices:** H&E stained	Reflectance	Adult	Tumor segmentation
27	Jong, L. S., et al. (2023). "Tissue Classification of Breast Cancer by Hyperspectral Unmixing." Cancers (Basel) **15**(10).	Tumor segmentation	SPECIM	**VIS:** PFD-CL-65-V10E; **NIR:** VLNIR CL-350-N17E	**VIS:** CMOS; **NIR:** InGaAs	Line-scanning (pushbroom)	**VIS:** 400 - 1000 nm (optimized: 450 - 951 nm); **NIR:** 900 - 1700 nm (optimized: 954 - 1650 nm)	Na	Na	**VIS:** 384 (optimized: 318); **NIR:** 256 (optimized: 210)	**VIS:** 3 nm; **NIR:** 5 nm	**VIS:** 0.16 mm/Px (1312 x 384 Px); **NIR:** 0.5 mm/Px (320 x 256 Px); **Combined VIS and NIR:** 0.5 mm/Px (320 x 256 Px)	Na	Na	Broadband halogen (unspecified)	Spectral unmixing; K-nearest neighbors	Accuracy: 87 %; Sensitivity: 94 %; Specificity: 85 %	Extra-operative	Breast	Ex-vivo	189	Fresh	Reflectance	Adult	Tumor segmentation
28	Kaneko, S., et al. (2022). "Fluorescence real-time kinetics of protoporphyrin IX after 5-ALA administration in low-grade glioma." J Neurosurg **136**(1): 9-15.	Fluorescence quantification	Na	Na	CMOS	Na	420 - 730 nm	Na	Liquid crystal tunable filter	Na	**Fluorescence spectra:** 3 nm; **White light spectra:** 5 nm; **Dark light spectra:** 3 nm	Na	Na	Na	LED; Blue light fluorescence module/filter [Zeiss BLUE 400]	Na	Na	Na	Brain	Ex-vivo	25	Fresh	Exogenous Fluorescence (5-ALA)	Adult	Fluorescence quantification
29	Kho, E., et al. (2019). "Broadband hyperspectral imaging for breast tumor detection using spectral and spatial information." Biomed Opt Express **10**(9): 4496-4515.	Tumor segmentation	SPECIM	**VIS:** PFD-CL-65-V10E; **NIR:** VLNIR CL-350-N17E	**VIS:** CMOS; **NIR:** InGaAs	Line-scanning (pushbroom)	**VIS:** 400 - 1000 nm (optimized: 450 - 951 nm); **NIR:** 900 - 1700 nm (optimized: 954 - 1650 nm)	Na	Na	**VIS:** 384 (optimized: 318); **NIR:** 256 (optimized: 210)	**VIS:** 3 nm; **NIR:** 5 nm	**VIS:** 0.16 mm/Px (1312 x 384 Px); **NIR:** 0.5 mm/Px (320 x 256 Px)	Na	**VIS:** 40 s; **NIR:** 20 s	Broadband halogen (unspecified)	Linear discriminant analysis; Convolutional neural network	Sensitivity [CNN]: 98 %; Specificity [CNN]: 99 %	Extra-operative	Breast	Ex-vivo	42	Fresh	Reflectance	Adult	Tumor segmentation
30	Kho, E., et al. (2021). "Feasibility of Ex Vivo Margin Assessment with Hyperspectral Imaging during Breast-Conserving Surgery: From Imaging Tissue Slices to Imaging Lumpectomy Specimen." Applied Sciences-Basel **11**(19): 19.	Tumor segmentation	SPECIM	**VIS:** PFD-CL-65-V10E; **NIR:** VLNIR CL-350-N17E	**VIS:** CMOS; **NIR:** InGaAs	Line-scanning (pushbroom)	**VIS:** 400 - 1000 nm (optimized: 450 - 951 nm); **NIR:** 900 - 1700 nm (optimized: 954 - 1650 nm)	Na	Na	**VIS:** 384 (optimized: 318); **NIR:** 256 (optimized: 210)	**VIS:** 3 nm; **NIR:** 5 nm	**VIS:** 0.16 mm/Px (1312 x 384 Px); **NIR:** 0.5 mm/Px (320 x 256 Px)	Na	**VIS:** 40 s; **NIR:** 20 s	Broadband halogen (unspecified)	Linear discriminant analysis	[Combined VIS and NIR] Performance of tumor detection - on slices: 79 % on lumpectomy specimens: 25 %	Extra-operative	Breast	Ex-vivo	94	Fresh (lumpectomy); Inked (slices) - I am not sure what this ink refers to	Reflectance	Adult	Tumor segmentation
31	Kho, E., et al. (2019). "Imaging depth variations in hyperspectral imaging: Development of a method to detect tumor up to the required tumor-free margin width." J Biophotonics **12**(11): e201900086.	Tumor segmentation	SPECIM	**VIS:** PFD-CL-65-V10E; **NIR:** VLNIR CL-350-N17E	**VIS:** CMOS; **NIR:** InGaAs	Line-scanning (pushbroom)	**VIS:** 400 - 1000 nm (optimized: 450 - 951 nm); **NIR:** 900 - 1700 nm (optimized: 954 - 1650 nm)	Na	Na	**VIS:** 384 (optimized: 318); **NIR:** 256 (optimized: 210)	**VIS:** 3 nm; **NIR:** 5 nm	**VIS:** 0.16 mm/Px (1312 x 384 Px); **NIR:** 0.5 mm/Px (320 x 256 Px)	Na	**VIS:** 40 s; **NIR:** 20 s	Broadband halogen (unspecified)	Spectral slope method	Na	Extra-operative	Breast; Phantom	Ex-vivo; Phantom	19	Inked (slices); Phantom	Reflectance	Adult	Tumor segmentation
32	Kho, E., et al. (2019). "Hyperspectral Imaging for Resection Margin Assessment during Cancer Surgery." Clin Cancer Res **25**(12): 3572-3580.	Tumor segmentation	SPECIM	VLNIR CL-350-N17E	InGaAs	Line-scanning (pushbroom)	900 - 1700 nm	Na	Na	256	5 nm	0.5 mm/Px (320 x 256 Px)	Na	~ 20 s	Broadband halogen (unspecified)	Linear support vector machine classifier	**Normal/IC:** Accuracy: 93 %; **Normal/DCIS:** Accuracy: 84 %	Extra-operative	Breast	Ex-vivo	18	Fresh (lumpectomy); Inked (slices)	Reflectance	Adult	Tumor segmentation
33	La Salvia, M., et al. (2022). AI-based segmentation of intraoperative glioblastoma hyperspectral images. Conference on Hyperspectral Imaging and Applications II, Birmingham, ENGLAND, Spie-Int Soc Optical Engineering.	Tumor segmentation	Headwall Photonics	Hyperspec® VNIR A-Series	Silicon-based CCD	Line-scanning (pushbroom)	400 - 1000 nm	Max: 129 × 230 mm	Na	826 (optimized: 128)	2 - 3 nm	1004 × 826 Px (with a linear displacement transducer: 1004 x 1787 Px)	Na	Na	Broadband halogen [quartz tungsten] (400 - 2200 nm)	Convolutional neural networks (U-Net++; DeepLabV3+ with backbones ResNet-50 and ResNet-50 3D)	Accuracy [U-Net++]: 61 %; Sensitivity [U-Net++]: 61 %; Specificity [U-Net++]: 72 %; Accuracy [DLV3 RN50]: 71 %; Sensitivity [DLV3 RN50]: 71 %; Specificity [DLV3 RN50]: 85 %; Accuracy [DLV3 RN3D]: 76 %; Sensitivity [DLV3 RN3D]: 76 %; Specificity [DLV3 RN3D]: 83 %	Intra-operative	Brain	In-vivo	H (16 to be neglected)	Fresh	Reflectance	Adult	Tumor segmentation
34	Lehtonen, S. J. R., et al. (2022). "Detection improvement of gliomas in hyperspectral imaging of protoporphyrin IX fluorescence - in vitro comparison of visual identification and machine thresholds." Cancer Treat Res Commun **32**: 100615.	Fluorescence quantification	Senop	HSC-2	CMOS	Snapshot	510 - 903 nm	36.8 degrees	Na	1000 (optimized: 4)	0.1 nm	1024 x 1024 Px	15 fps	0.463 s (per hypercube)	Xenon (white light); Blue light fluorescence module/filter [Leica FL400]	Na	Recognition rate: 96 %; Sensitivity: 100 %; Specificity: 86 %; Positive predictive value: 94 %; Negative predictive value: 100 %	Extra-operative	Phantom	Phantom	0	Phantom (samples with protoporphyrin IX)	Exogenous Fluorescence (5-ALA)	Na	Fluorescence quantification; Tumor segmentation
35	Leitch, K., et al. (2022). "Detecting Aggressive Papillary Thyroid Carcinoma Using Hyperspectral Imaging and Radiomic Features." Proc SPIE Int Soc Opt Eng **12033**.	Tumor segmentation; Tumor grade prediction	Perkin Elmer	CRI Maestro	Silicon-based CCD	Na	450 - 900 nm	Na	Liquid crystal tunable filter	91	5 nm	25 µm (1040 × 1392 Px)	Na	~ 1 min	Na	Support vector machine classifier; Logistic regression; Random forest classifier; K-nearest neighbors; Gaussian naïve bayes; Decision tree classifier; Extra trees; AdaBoost classifier; Gradient boosting classifier; Stochastic gradient descent classifier; Gaussian process classification; Perceptron; Bernoulli naïve bayes; Linear discriminant analysis; Quadratic discriminant analysis; eXtreme gradient boosting	**Non-dilated:** Accuracy: 83.3 %; **Dilated:** Accuracy: 100 %	Extra-operative	Thyroid	Ex-vivo	44	Fresh	Reflectance	Na	Tumor segmentation; Tumor grade prediction
36	Leon, R., et al. (2021). "VNIR-NIR hyperspectral imaging fusion targeting intraoperative brain cancer detection." Sci Rep **11**(1): 19696.	Tumor segmentation	Headwall Photonics	**VNIR:** Hyperspec® VNIR A-Series; **NIR:** Hyperspec® NIR 100/U	**VNIR:** Silicon-based CCD; **NIR:** InGaAs	Line-scanning (pushbroom)	**VIS:** 400 - 1000 nm; **NIR:** 900 - 1700 nm (**optimized VIS:** 435 - 901 nm; **optimized NIR:** 956 - 1638 nm)	**NIR:** IFOV: 0.402 nm; **VNIR:** IFOV: 0.137 nm	Na	**VNIR:** 641; **NIR:** 144	Na	Na	Na	~ 1 min	Broadband halogen [quartz tungsten] (400 - 2200 nm)	K-means, K-medoids, and Hierarchical K-means clustering; Support vector machine classifier; K-nearest neighbors classifier; Random forest classifier	Na (fusing VNIR - NIR data improves the classification results up to 21% of accuracy with respect to the use of each data modality independently)	Intra-operative	Brain	In-vivo	Na	Fresh; Polymer	Reflectance	Adult	Tumor segmentation
37	Leon, R., et al. (2022). Hyperspectral Imaging for In-Vivo/Ex-Vivo Tissue Analysis of Human Brain Cancer. Conference on Medical Imaging - Image-Guided Procedures, Robotic Interventions, and Modeling, Electr Network, Spie-Int Soc Optical Engineering.	Tumor segmentation	Headwall Photonics	Hyperspec® VNIR A-Series	Silicon-based CCD	Line-scanning (pushbroom)	400 - 1000 nm (optimized: 440 - 909 nm)	Na	Na	826 (optimized: 645)	2 - 3 nm	1004 Px	Na	Na	Broadband halogen [quartz tungsten] (400 - 2200 nm)	K-means clustering; R545/R560 spectral ratio method	Na	Intra-operative	Brain	In-vivo; Ex-vivo	7	Fresh	Reflectance	Adult	Tumor segmentation
38	Liu, S., et al. (2020). "Using hyperspectral imaging automatic classification of gastric cancer grading with a shallow residual network." Anal Methods **12**(30): 3844-3853.	Tumor segmentation; Tumor grade prediction	Experimental	Experimental	Na	Line-scanning (pushbroom)	374 - 990 nm (optimized: 429 - 910 nm)	Na	Na	256 (optimized: 200)	2.41 nm	0.5 µm (696 x 846 Px)	Na	< 30 s	Broadband halogen [quartz tungsten] (400 - 2500 nm, 50 W)	Shallow residual network; Convolutional neural network; Random forest classifier; Support vector machine classifier; Quadratic discriminant classifier	Accuracy [SR-Net]: 91.44 %	Extra-operative	Stomach	Ex-vivo	30	H&E stained	Reflectance	Adult	Tumor segmentation; Tumor grade prediction
39	Lu, G., et al. (2014). "Hyperspectral Imaging for Cancer Surgical Margin Delineation: Registration of Hyperspectral and Histological Images." Proc SPIE Int Soc Opt Eng **9036**: 90360s.	Tumor segmentation	Perkin Elmer	CRI Maestro	Silicon-based CCD	Na	450 - 950 nm	from 3.4 x 2.5 cm to 10.2 x 7.6 cm	Liquid crystal tunable filter	251	2 nm	1,392 x 1,040 Px	Na	Na	Xenon (500 - 950 nm)	Na	Na	Extra-operative	Head; Neck	In-vivo (animal); Ex-vivo (animal)	0	Fresh	Reflectance	Animal (mouse)	Tumor segmentation
40	Lu, G., et al. (2017). "Detection of Head and Neck Cancer in Surgical Specimens Using Quantitative Hyperspectral Imaging." Clin Cancer Res **23**(18): 5426-5436.	Tumor segmentation	Perkin Elmer	CRI Maestro	Silicon-based CCD	Na	450 - 900 nm	Na	Liquid crystal tunable filter	91	5 nm	Na	Na	Na	Xenon (500 - 950 nm)	Linear discriminant analysis; Quadratic discriminant analysis; Ensemble linear discriminant analysis; Linear support vector machine; Kernel support vector machine with radial basis function function; Random forest [classifiers]	Sensitivity [ELDA]: 91 %; Specificity [ELDA]: 91 %	Extra-operative	Oral cavity; Larynx; Pharynx; Maxillary sinus; Nasal cavity; Thyroid; Parotid	Ex-vivo	36	Fresh	Reflectance	Adult	Tumor segmentation
41	Lu, G., et al. (2015). "Quantitative Wavelength Analysis and Image Classification for Intraoperative Cancer Diagnosis with Hyperspectral Imaging." Proc SPIE Int Soc Opt Eng **9415**.	Tumor segmentation	Perkin Elmer	CRI Maestro	Silicon-based CCD	Na	450 - 900 nm	Na	Liquid crystal tunable filter	226	2 nm	Na	Na	Na	Xenon (500 - 950 nm)	Support vector machine classifier + Active contour	Sensitivity: 94.4 %; Specificity: 98.3 %	Extra-operative	Head; Neck	In-vivo (animal)	0	Fresh	Reflectance	Animal (mouse)	Tumor segmentation
42	Lu, G., et al. (2015). "Framework for hyperspectral image processing and quantification for cancer detection during animal tumor surgery." J Biomed Opt **20**(12): 126012.	Tumor segmentation	Perkin Elmer	CRI Maestro	Silicon-based CCD	Spectral-scanning	450 - 900 nm	Na	Liquid crystal tunable filter	226	2 nm	25 µm/Px	Na	4 - 7 s (one hypercube)	Xenon (500 - 950 nm)	K-nearest neighbors	Accuracy: 67.2 %; Sensitivity: 77.5 %; Specificity 54.0 %	Extra-operative	Head; Neck	In-vivo (animal)	0	Fresh	Reflectance	Animal (mouse)	Tumor segmentation
43	Ma, L., et al. (2019). "Adaptive deep learning for head and neck cancer detection using hyperspectral imaging." Vis Comput Ind Biomed Art **2**(1): 18.	Tumor segmentation	Perkin Elmer	CRI Maestro	Silicon-based CCD	Na	450 - 950 nm	from 3.4 x 2.5 cm to 10.2 x 7.6 cm	Liquid crystal tunable filter	251	2 nm	1,040 x 1,392 Px (ROI: 435 x 390 Px)	Na	Na	Xenon (500 - 950 nm)	Adaptive deep learning algorithm (based on auto-encoder); Convolutional neural network	Sensitivity: 92.32 %; Specificity: 91.31 %	Extra-operative	Head; Neck	In-vivo (animal)	0	Fresh	Reflectance	Animal (mouse)	Tumor segmentation
44	Ma, L., et al. (2017). "Deep Learning based Classification for Head and Neck Cancer Detection with Hyperspectral Imaging in an Animal Model." Proc SPIE Int Soc Opt Eng **10137**.	Tumor segmentation	Perkin Elmer	CRI Maestro	Silicon-based CCD	Na	450 - 950 nm	from 3.4 x 2.5 cm to 10.2 x 7.6 cm	Liquid crystal tunable filter	251	2 nm	1,040 x 1,392 Px (ROI: 435 x 390 Px)	Na	Na	Xenon (500 - 950 nm)	Convolutional neural network	Accuracy: 91.36 %; Sensitivity: 86.05 %; Specificity 93.36 %	Extra-operative	Head; Neck	In-vivo (animal)	0	Fresh	Reflectance	Animal (mouse)	Tumor segmentation
45	Ma, L., et al. (2021). "Pixel-level Tumor Margin Assessment of Surgical Specimen with Hyperspectral Imaging and Deep Learning Classification." Proc SPIE Int Soc Opt Eng **11598**.	Tumor segmentation	Perkin Elmer	CRI Maestro	Silicon-based CCD	Na	450 - 900 nm	from 6.4 mm × 6.4 mm to 19.2 mm × 16 mm (median size: 12.8 mm × 9.6 mm)	Liquid crystal tunable filter	91	5 nm	25 µm/Px	Na	Na	Na	Two-dimensional fully convolutional network (based on the U-Net architecture)	AUC: 88 %; Accuracy: 83 %; Sensitivity: 84 %; Specificity: 70 %	Na	Larynx; Hypopharynx	Ex-vivo	20	Fresh; H&E stained	Reflectance	Adult	Tumor segmentation
46	MacCormac, O., et al. (2023). "Lightfield hyperspectral imaging in neuro-oncology surgery: an IDEAL 0 and 1 study." Front Neurosci **17**: 1239764.	Tumor segmentation	Cubert GmbH	Ultris ×50	Na	Snapshot (lightfield)	350 - 1000 nm	Na	Na	155	Na	Na	1 Hz	Na	Xenon (350 - 1000 nm)	Na	Na	Intra-operative	Brain	In-vivo	1	Fresh	Reflectance	Adult	Tumor segmentation
47	Maktabi, M., et al. (2019). "Tissue classification of oncologic esophageal resectates based on hyperspectral data." Int J Comput Assist Radiol Surg **14**(10): 1651-1661.	Tumor segmentation	Diaspective Vision GmbH	TIVITA	CMOS	Line-scanning (pushbroom)	500 - 1000 nm (optimized: 525 - 610 nm)	Na	Na	Na	5 nm	0.1 mm/Px (640 x 480 Px)	Na	Na	Broadband halogen (unspecified)	Support vector machine classifier; Random forest classifier; K-nearest neighbors; Multi-layer perceptron	Sensitivity [SVM]: 63 %; Specificity [SVM]: 69 %	Intra-operative	Stomach; Esophagus	Ex-vivo	11	Fresh	Reflectance	Adult	Tumor segmentation
48	Maktabi, M., et al. (2021). "Using physiological parameters measured by hyperspectral imaging to detect colorectal cancer." Annu Int Conf IEEE Eng Med Biol Soc **2021**: 3865-3868.	Tumor segmentation	Diaspective Vision GmbH	TIVITA	CMOS	Line-scanning (pushbroom)	500 - 1000 nm	Na	Na	Na	Na	Na	Na	6 s	Na	Logistic regression; Support vector machine classifier with linear and radial basis kernel; Random forest classifier; Convolutional neural network; K-nearest neighbor	AUC [CNN]: 96 %; Sensitivity [CNN]: 91 %; Specificity [CNN]: 100 %	Intra-operative	Colorectal	Ex-vivo	52	Fresh	Reflectance	Adult	Tumor segmentation
49	Manni, F., et al. (2021). Hyperspectral imaging for tissue classification in glioblastoma tumor patients: a deep spectral-spatial approach. Conference on Medical Imaging - Image-Guided Procedures, Robotic Interventions, and Modeling, Electr Network, Spie-Int Soc Optical Engineering.	Tumor segmentation	Headwall Photonics	Hyperspec® VNIR A-Series	Silicon-based CCD	Line-scanning (pushbroom)	400 - 1000 nm	Na	Na	826	2 - 3 nm	0.1287 mm/Px	Na	Na	Na	Three-dimensional and two-dimensional hybrid convolutional neural network-based approach (HybridSN); Support vector machine classifier; Two-dimensional convolutional neural network	Accuracy [3D–2D hybrid CNN]: 77 %; Sensitivity [3D–2D hybrid CNN]: 33 %; Specificity [3D–2D hybrid CNN]: 95 %	Intra-operative	Brain	In-vivo	9	Fresh	Reflectance	Adult	Tumor segmentation
50	Manni, F., et al. (2020). "Hyperspectral imaging for colon cancer classification in surgical specimens: towards optical biopsy during image-guided surgery." Annu Int Conf IEEE Eng Med Biol Soc **2020**: 1169-1173.	Tumor segmentation	IMEC	Na	Silicon-based CCD	Line-scanning (pushbroom)	430 - 920 nm	Na	Na	145	Na	Na	Na	Na	Broadband halogen (unspecified)	HybridSpectraNet convolutional neural network; ResNet50 convolutional neural network; 3D convolutional neural network	AUC [HybridSN]: 82 %; Sensitivity [HybridSN]: 88 %; Specificity [HybridSN]: 78 %	Extra-operative	Colorectal	Ex-vivo	6	Fresh	Reflectance	Adult	Tumor segmentation
51	Manni, F., et al. (2020). "Hyperspectral Imaging for Glioblastoma Surgery: Improving Tumor Identification Using a Deep Spectral-Spatial Approach." Sensors (Basel) **20**(23).	Tumor segmentation	Headwall Photonics	Hyperspec® VNIR A-Series	Silicon-based CCD	Line-scanning (pushbroom)	400 - 1000 nm	Na	Na	826	2 - 3 nm	1004 x 826 Px	Na	Na	Broadband halogen [quartz tungsten] (400 - 2200 nm)	Three-dimensional and two-dimensional hybrid convolutional neural network-based approach; Two-dimensional convolutional neural network; One-dimensional deep neural network; Support vector machine classifier; Support vector machine classifier combined with the 3D–2D hybrid CNN	Accuracy [3D–2D hybrid CNN]: 80 %; Sensitivity [3D–2D hybrid CNN]: 68 %; Specificity [3D–2D hybrid CNN]: 98 %; AUC [3D–2D hybrid CNN]: 70 %	Intra-operative	Brain	In-vivo	H (16 to be neglected)	Fresh	Reflectance	Adult	Tumor segmentation
52	Manni, F., et al. (2019). Automated tumor assessment of squamous cell carcinoma on tongue cancer patients with hyperspectral imaging. Conference on Medical Imaging - Image-Guided Procedures, Robotic Interventions, and Modeling, San Diego, CA, Spie-Int Soc Optical Engineering.	Tumor segmentation	Philips Research + IMEC	Prototype model mounting CMOSIS CMV2000 image sensor	CMOS	Line-scanning (pushbroom)	430 - 920 nm	Na	Na	192	Na	2048 Px per line	Na	Na	Na	Support vector machine classifier	AUC: 92 %; Sensitivity: 94 %; Specificity 68 %	Na	Tongue	Ex-vivo	7	Fresh	Reflectance	Adult	Tumor segmentation
53	Martinez, B., et al. (2019). "Most Relevant Spectral Bands Identification for Brain Cancer Detection Using Hyperspectral Imaging." Sensors (Basel) **19**(24).	Tumor segmentation	Headwall Photonics	Hyperspec® VNIR A-Series	Silicon-based CCD	Line-scanning (pushbroom)	400 - 1000 nm	Max: 129 × 230 mm	Na	826 (optimized: 48)	2 - 3 nm	128.7 µm/Px (1004 × 826 Px) (with a linear displacement transducer: 1004 x 1787 Px)	Na	Na	Broadband halogen [quartz tungsten] (400 - 2200 nm)	Support vector machine classifier	Accuracy: 77.9 %; Sensitivity: 52.7 %; Specificity 94.6 %	Intra-operative	Brain	In-vivo	H (16 to be neglected)	Fresh	Reflectance	Adult	Tumor segmentation
54	Martínez-González, A., et al. (2020). Can Hyperspectral Images be used to detect Brain tumor pixels and their malignant phenotypes? 35th Conference on Design of Circuits and Integrated Systems (DCIS), Electr Network, Ieee.	Tumor segmentation	Headwall Photonics	Hyperspec® VNIR A-Series	Silicon-based CCD	Line-scanning (pushbroom)	400 - 1000 nm	Na	Na	**In-vivo:** 128; **In-vitro/Ex-vivo:** 159	Na	**In-vivo:** 124,033 Px; **In-vitro/Ex-vivo:** 602,400 Px	Na	Na	Na	Patient specific pixel-based method (proposed)	**In-vivo:** Sensitivity: 89 %, Specificity: 96 % ; **In-vitro/Ex-vivo:** Sensitivity: 86 %, Specificity: 92 %	Intra-operative; Extra-operative	Brain	In-vivo; Ex-vivo	13	Fresh; H&E stained	Reflectance	Adult	Tumor segmentation
55	Mendoza-Chavarría, J. N., et al. (2022). Glioblastoma Classification in Hyperspectral Images by Nonlinear Unmixing. 25th Euromicro Conference on Digital System Design (DSD), Maspalomas, SPAIN, Ieee.	Tumor segmentation	Headwall Photonics	Hyperspec® VNIR A-Series	Silicon-based CCD	Line-scanning (pushbroom)	400 - 1000 nm	Na	Na	826	2 - 3 nm	1004 Px per line	**Min:** 90 fps	Na	Broadband halogen [quartz tungsten] (400 - 2200 nm)	Non-linear extended blind end-member and abundance extraction algorithm (spectral unmixing) + Abundance map	Accuracy: 90 %; Sensitivity: 55 %; Specificity 97 %; F1-score: 88 %	Intra-operative	Brain	In-vivo	H (_ to be neglected)	Fresh	Reflectance	Adult	Tumor segmentation
56	Meng, B., et al. (2020). "Topical dual-probe staining using quantum dot-labeled antibodies for identifying tumor biomarkers in fresh specimens." PLoS One **15**(3): e0230267.	Fluorescence quantification	Experimental	Experimental	CMOS	Na	500 - 700 nm	Na	Liquid crystal tunable filter	40	5 nm	55 µm/Px	Na	~ 1 min	LED (405 nm)	Na	Na	Extra-operative	Breast	Ex-vivo (animal); Phantom	0	Fresh; Phantom	Exogenous Fluorescence (QD)	Animal (mouse)	Fluorescence quantification
57	Mühle, R., et al. (2020). "Workflow and hardware for intraoperative hyperspectral data acquisition in neurosurgery." Biomed Tech (Berl).	Tumor segmentation	Diaspective Vision GmbH	TIVITA	CMOS	Line-scanning (pushbroom)	500 - 1000 nm	Na	Na	Na	5 nm	1,280 x 960 Px	Na	1 min	Broadband halogen (unspecified)	Random forest classifier	Accuracy: 99.1 %; Sensitivity: > 98 %; Specificity > 98 %	Intra-operative	Brain	In-vivo	1	Fresh	Reflectance	Adult	Tumor segmentation
58	Pardo, A., et al. (2017). "Directional Kernel Density Estimation for Classification of Breast Tissue Spectra." IEEE Trans Med Imaging **36**(1): 64-73.	Tumor segmentation	Experimental	Experimental	Silicon-based CCD	Na	510 - 785 nm	Na	Na	Na	1 nm	Na	Na	Na	Broadband halogen [quartz tungsten]	Directional kernel density estimation + Maximum likelihood classification	Sensitivity: 98 %; Specificity 97 %	Extra-operative	Breast	Ex-vivo	Na	Fresh	Reflectance	Adult	Tumor segmentation
59	Peller, J., et al. (2017). Hyperspectral imaging based on compressive sensing to determine cancer margins in human pancreatic tissue ex vivo. Conference on Optical Biopsy XV - Toward Real-Time Spectroscopic Imaging and Diagnosis, San Francisco, CA, Spie-Int Soc Optical Engineering.	Tumor segmentation	**Spectromer:** Ocean Insight; **Digital micromirror device:** Texas Instruments	**Spectromer:** QE Pro Series; **Digital micromirror device:** DLP5500 0.55 XGA	Na	Spectrometer + Digital micromirror device	350 - 750 nm	1.9 cm × 1.9 cm	Na	Na	Na	0.44 mm/Px (1024 × 768 Px)	Na	Na	Halogen (unspecified)	Spectral angle mapper	Sensitivity: 74.50 %; Specificity: 75.53 %	Extra-operative	Pancreas	Ex-vivo	6	Fresh	Reflectance	Adult	Tumor segmentation
60	Pertzborn, D., et al. (2022). "Intraoperative Assessment of Tumor Margins in Tissue Sections with Hyperspectral Imaging and Machine Learning." Cancers (Basel) **15**(1).	Tumor segmentation	Diaspective Vision GmbH	TIVITA	CMOS	Line-scanning (pushbroom)	500 - 1000 nm	Na	Na	Na	10 nm	720 x 540 Px	Na	4 s (per image); 5 - 15 min (per sample)	Broadband halogen (unspecified)	Linear support vector machine classifier; Three-dimensional convolutional neural network (based on a deep residual network)	Accuracy [3D-CNN]: 76 %; Specificity [3D–2D hybrid CNN]: 89 %; Sensitivity [3D–2D hybrid CNN]: 48 %	Extra-operative	Oropharynx	Ex-vivo	7	Fresh	Reflectance	Adult	Tumor segmentation
61	Puustinen, S., et al. (2023). "Hyperspectral Imaging in Brain Tumor Surgery-Evidence of Machine Learning-Based Performance." World Neurosurg **175**: e614-e635.	Tumor segmentation; Tissue identification	Senop	HSC-2	CMOS	Snapshot	500 - 900 nm	Na	Na	Na	Na	1024 x 1024 Px	Max: 74 fps (12 bit); 149 fps (10 bit	Na	Xenon (unspecified)	light gradient boosting machine decision tree; Convolutional neural network	**Tumor margin assessment:** Accuracy [LightGBM]: 97.7 %; **Tissue identification:** Accuracy [CNN]: 98 %;	Intra-operative	Brain	In-vivo	1	Fresh	Reflectance	Adult	Tumor segmentation; Tissue identification
62	Ravi, D., et al. (2017). "Manifold Embedding and Semantic Segmentation for Intraoperative Guidance With Hyperspectral Brain Imaging." IEEE Trans Med Imaging **36**(9): 1845-1857.	Tumor segmentation	Headwall Photonics	**VNIR:** Hyperspec® VNIR A-Series; **NIR:** Hyperspec® NIR 100/U	**VNIR:** Silicon-based CCD; **NIR:** InGaAs	Line-scanning (pushbroom)	**VNIR:** 400 - 1000 nm (optimized: 450 - 950 nm); **NIR:** 900 - 1700 nm (optimized: 950 - 1650 nm)	Na	Na	**VNIR:** 826; **NIR:** 172	**VNIR:** 2 - 3 nm; **NIR:** Na	**VNIR:** 128.7 µm x 128.7 µm (per pixel); **NIR:** 0.48 µm × 0.48 µm (per pixel)	Na	Na	Broadband (unspecified)	Semantic Texton Forest; Discrete Cosine Transform - Semantic Texton Forest	**VNIR:** Accuracy [DCT-STF]: ~ 81 %; Sensitivity [DCT-STF]: ~ 84 %; Specificity [DCT-STF]: ~ 86 %; **NIR:** Accuracy [DCT-STF]: ~ 62 %; Sensitivity [DCT-STF]: ~ 46 %; Specificity [DCT-STF]: ~ 91 %	Intra-operative	Brain	In-vivo	18	Fresh	Reflectance	Adult	Tumor segmentation
63	Sancho, J., et al. (2023). "SLIMBRAIN: Augmented reality real-time acquisition and processing system for hyperspectral classification mapping with depth information for in-vivo surgical procedures." Journal of Systems Architecture **140**: 14.	Tumor segmentation	Ximea	MQ022HG-IM-SM5X5-NIR2	CMOS	Snapshot	665 - 960 nm	Na	Na	25	Na	5.5 µm/Px (409 x 217 Px)	14 fps	Na	Broadband halogen (unspecified)	Support vector machine classifier + K-means clustering + Majority voting	AUC: 95.17 %	Intra-operative	Brain	In-vivo	Na	Fresh	Reflectance	Adult	Tumor segmentation; Augmented reality guidance
64	Sato, D., et al. (2021). "Author Correction: Distinction of surgically resected gastrointestinal stromal tumor by near-infrared hyperspectral imaging." Sci Rep **11**(1): 19030.	Tumor segmentation	Sumitomo Electric Industries	CompoVision CV-N800HS	Na	Na	1000 - 2350 nm	Na	Na	256	6.3 nm	Na	320 fps (per wavelength band)	2.5 ms (per wavelength band)	Broadband halogen (unspecified)	Support vector machine classifier	Accuracy: 79.7 %; Sensitivity: 77.2 %; Specificity 74.8 %	Extra-operative	Stomach	Ex-vivo	12	Fresh	Reflectance	Adult	Tumor segmentation
65	Schwake, M., et al. (2019). "Spectroscopic measurement of 5-ALA-induced intracellular protoporphyrin IX in pediatric brain tumors." Acta Neurochir (Wien) **161**(10): 2099-2105.	Fluorescence quantification	Na	Na	Na	Na	420 - 730 nm	Na	Na	Na	**Fluorescence spectra:** 3 nm; **White light spectra:** 5 nm	Na	Na	Na	LED; Blue light fluorescence module/filter [Zeiss BLUE 400]	Na	Na	Intra-operative	Brain	Ex-vivo	11	Fresh	Exogenous Fluorescence (5-ALA)	Pediatric	Fluorescence quantification
66	Stewart, S., et al. (2020). "Visible near infrared reflectance molecular chemical imaging of human ex vivo carcinomas and murine in vivo carcinomas." J Biomed Opt **25**(2): 1-18.	Tumor segmentation	Teledyne Princeton Instruments	Na	Silicon-based CCD	Na	400 - 1100 nm (optimized: 520 - 1050 nm)	**In vivo (animal):** 100 mm × 100 mm; **Ex-vivo:** 163 mm x 124 mm	Liquid crystal tunable filter	141	5 nm	**In vivo (animal):** 0.40 mm; **Ex-vivo:** 0.80 mm	Na	Na	Broadband halogen (400 - 1100 nm)	Partial least squares discriminant analysis; Ratiometric score	**In-vivo (animal):** AUC: 96 %; **Ex-vivo:** Accuracy: 89 %; Sensitivity: 100 %; Specificity: 83 %	Extra-operative	**In vivo (animal):** Breast; Lung; **Ex-vivo:** Kidney	In-vivo (animal); Ex-vivo (animal and human)	22	Fresh	Reflectance	Adult (human); Animal	Tumor segmentation
67	Suero Molina, E., et al. (2021). "5-Aminolevulinic Acid-Induced Porphyrin Contents in Various Brain Tumors: Implications Regarding Imaging Device Design and Their Validation." Neurosurgery **89**(6): 1132-1140.	Fluorescence quantification	Na	Na	CMOS	Na	420 - 730 nm	Na	Liquid crystal tunable filter	Na	Fluorescence spectra: 3 nm; White light spectra: 5 nm; Dark light spectra: 3 nm	Na	Na	Na	LED; Blue light fluorescence module/filter [Zeiss BLUE 400]	Spectral unmixing	Na	Na	Brain	Ex-vivo	162	Fresh	Exogenous Fluorescence (5-ALA)	Adult; Pediatric	Fluorescence quantification
68	Trajanovski, S., et al. (2021). "Tongue Tumor Detection in Hyperspectral Images Using Deep Learning Semantic Segmentation." IEEE Trans Biomed Eng **68**(4): 1330-1340.	Tumor segmentation	SPECIM	**VIS:** PFD-CL-65-V10E; **NIR:** VLNIR CL-350-N17E	**VIS:** CMOS; **NIR:** InGaAs	Line-scanning (pushbroom)	**VIS:** 400 - 1000 nm; **NIR:** 900 - 1700 nm	Na	Na	**VIS:** 384; **NIR:** 256	Na	Na	Na	Na	Broadband halogen (unspecified)	Several deep neural network designs (Structural, Spectral, Dual stream, U-Net, Dual U-Net)	Accuracy [U-Net Stacked 128ch]: 95.8 %; Sensitivity [U-Net Stacked 128ch]: 87.3 %; Specificity [U-Net Stacked 128ch]: 97.5 %; AUC [U-Net Stacked 128ch]: 92.4 %	Extra-operative	Tongue	Ex-vivo	14	Fresh	Reflectance	Adult	Tumor segmentation
69	Urbanos, G., et al. (2021). "Supervised Machine Learning Methods and Hyperspectral Imaging Techniques Jointly Applied for Brain Cancer Classification." Sensors (Basel) **21**(11).	Tumor segmentation; Tissue identification	Ximea	MQ022HG-IM-SM5X5-NIR2	CMOS	Snapshot	665 - 975 nm	Na	Na	25	Na	5.5 µm/Px (409 x 217 Px)	**Max:** 170 hypercubes/ second	Na	Broadband halogen (unspecified)	Support vector machine classifier; Random forest classifier; Three-dimensional convolutional neural network	Accuracy [SVM]: 60.1 %; Accuracy [RF]: 52.9 %; Accuracy [3D-CNN]: 49.1 %	Intra-operative	Brain	In-vivo	12	Fresh	Reflectance	Adult	Tumor segmentation; Tissue identification
70	van Vliet-Pérez, S. M., et al. (2022). "Hyperspectral Imaging for Tissue Classification after Advanced Stage Ovarian Cancer Surgery-A Pilot Study." Cancers (Basel) **14**(6).	Tumor segmentation	IMEC	Na	CMOS	Snapshot	665 - 975 nm	Na	Na	25	< 15 nm	2050 x 1080 Px	10 hypercubes/ second	Na	Broadband halogen (unspecified)	Linear support vector machine classifier	Sensitivity: 81 %; Specificity: 70 %	Extra-operative	Ovaries; Fallopian tubes; Uterus; Omentum; Intestines	Ex-vivo	11	Fresh	Reflectance	Adult	Tumor segmentation
71	Walke, A., et al. (2023). "Challenges in, and recommendations for, hyperspectral imaging in ex vivo malignant glioma biopsy measurements." Sci Rep **13**(1): 3829.	Fluorescence quantification	Experimental	Experimental	CMOS	Na	420 - 730 nm	Na	Liquid crystal tunable filter	Na	**Reflectance:** 5 nm; **Fluorescence:** 3 nm; **Background:** 3 nm	210 × 210 µm (per pixel)	Na	3 min (per sample)	LED (405 nm + Broadband white); Blue light fluorescence module/filter [Zeiss BLUE 400]	Na	Na	Extra-operative	**Human:** Brain; **Animal (pig):** Brain (homogenate); Phantom (brain)	Ex-vivo (animal and human); Phantom	26	**Human:** Fresh; **Animal (pig):** Homogenized, 0.5 M trishydroxymethylaminomethane buffer (prepared with hydrochloric acid), pH 5 - 9; (liquid) Phantom	Exogenous Fluorescence (5-ALA)	Adult; Animal (pig)	Fluorescence quantification
72	Wang, R., et al. (2020). "Classification and Segmentation of Hyperspectral Data of Hepatocellular Carcinoma Samples Using 1-D Convolutional Neural Network." Cytometry A **97**(1): 31-38.	Tumor segmentation	Finger Lakes Instrumentation	MLX285	Silicon-based CCD	Na	400 - 720 nm	Na	Liquid crystal tunable filter	107	3 nm	6.45 µm/Px (1360 x 1024 Px)	Na	Na	Na	One-dimensional convolutional neural network; Support vector machine classifier; Random forest classifier	Sensitivity [1D-CNN]: 87.1 %; Specificity [1D-CNN]: 88.8 % ; AUC [1D-CNN]: 95 %; Sensitivity [SVM]: 80.6 %; Specificity [SVM]: 89.5 %; AUC [SVM]: 93 %; Sensitivity [RF]: 77 %; Specificity [RF]: 91 %; AUC [RF]: 93.9 %	Extra-operative	Liver	Ex-vivo	14	Na	Reflectance	Adult	Tumor segmentation
73	Zhang, Y., et al. (2021). "Explainable liver tumor delineation in surgical specimens using hyperspectral imaging and deep learning." Biomed Opt Express **12**(7): 4510-4529.	Tumor segmentation	SPECIM	FX10	CMOS	Line-scanning (pushbroom)	400 - 1000 nm	12, 24, 38 (default), 47, 51, 83 degrees	Na	224	**Mean FWHM:** 5.5 nm	1024 x 1 Px	**Max:** 327 fps	Na	Broadband halogen (unspecified)	Support vector machine classifier; Convolutional neural network (U-Net)	Accuracy [U-Net]: 93.16 %; Sensitivity [U-Net]: 94.48 %; Specificity [U-Net]: 87.22 %; DSC [U-Net]: 92.91 %	Na	Liver	Ex-vivo	19	Fresh	Reflectance	Adult	Tumor segmentation
74	Aref, M., Aboughaleb, I. H. ., Youssef, A.-B. . and El-Sharkawy, Y. . (2020) "Novel Approach Exploiting the Hyperspectral Imaging System for Breast Cancer Therapy and Diagnosis", Archives of Breast Cancer, pp. 189–201.	Tumor segmentation	Surface Optics	SOC710	Silicon-based CCD	Line-scanning (pushbroom)	379 - 1050 nm	Na	Na	128 (optimized: 7)	3.1 nm (optimized: 100 nm)	600 x 502 Px	Na	3.65 s	Broadband (380 - 1050 nm)	Na	Na	Extra-operative	Breast	Ex-vivo	10	Fresh	Reflectance	Adult	Tumor segmentation
75	Lu, G., Halig, L., Wang, D., Qin, X., Chen, Z. G., & Fei, B. (2014). Spectral-spatial classification for noninvasive cancer detection using hyperspectral imaging. Journal of biomedical optics, 19(10), 106004.	Tumor segmentation	Perkin Elmer	CRI Maestro	Silicon-based CCD	Na	450 - 900 nm	from 3.4 x 2.5 cm to 10.2 x 7.6 cm	Liquid crystal tunable filter	226 (optimized: 224)	2 nm	1040 × 1392 Px	Na	Na	Broadband (450 - 900 nm)	Support vector machine classifier + Active contour; K-nearest neighbors	Sensitivity: 93.7 %; Specificity: 91.3 %	Extra-operative	Head; Neck	In-vivo (animal)	0	Fresh	Reflectance	Animal (mouse)	Tumor segmentation
76	Kaneko, S., Suero Molina, E., Ewelt, C., Warneke, N., & Stummer, W. (2019). Fluorescence-Based Measurement of Real-Time Kinetics of Protoporphyrin IX After 5-Aminolevulinic Acid Administration in Human In Situ Malignant Gliomas. Neurosurgery, 85(4), E739–E746.	Fluorescence quantification	Na	Na	CMOS	Na	420 - 730 nm	Na	Liquid crystal tunable filter	Na	**Fluorescence spectra:** 3 nm; **White light spectra:** 5 nm; **Dark light spectra:** 3 nm	Na	Na	Na	LED; Blue light fluorescence module/filter [Zeiss BLUE 400]	Spectral unmixing	Na	Intra-operative	Brain	Ex-vivo	68	Fresh	Exogenous Fluorescence (5-ALA)	Adult	Fluorescence quantification
77	Peller, J. , McGinnis, C. , Thompson, K. , Siddiqui, I. , Martinie, J. , Iannitti, D. and Trammell, S. (2021) Hyperspectral Imaging Based on Compressive Sensing: Determining Cancer Margins in Human Pancreatic Tissue *ex Vivo*, a Pilot Study. *Open Journal of Medical Imaging*, **11**, 115-131.	Tumor segmentation	**Spectromer:** Ocean Insight; **Digital micromirror device:** Texas Instruments	**Spectromer:** QE Pro Series; **Digital micromirror device:** DLP5500 0.55 XGA	Na	Spectrometer + Digital micromirror device	350 - 750 nm	1.9 cm × 1.9 cm	Na	Na	Na	0.44 mm/Px (1024 × 768 Px)	Na	7 min (per image)	Halogen (unspecified); Xenon (unspecified)	Support vector machine classifier; Spectral angle mapper	Na	Extra-operative	Pancreas	Ex-vivo	19	Fresh	Reflectance	Adult	Tumor segmentation
78	Jong, L. S., Appelman, J. G. C., Sterenborg, H., Ruers, T. J. M., & Dashtbozorg, B. (2024). Spatial and Spectral Reconstruction of Breast Lumpectomy Hyperspectral Images. Sensors (Basel), 24(5). https://doi.org/10.3390/s24051567	Tumor segmentation	SPECIM	PFD-CL-65-V10E	CMOS	Line-scanning (pushbroom)	400 - 1000 nm	Na	Na	384	3 nm	0.16 mm/Px (1312 x 384 Px)	Na	40 s	Broadband halogen (unspecified)	Na	Na	Extra-operative	Breast	Ex-vivo	Na	Fresh	Reflectance	Adult	Tumor segmentation
79	Kifle, N., Teti, S., Ning, B., Donoho, D. A., Katz, I., Keating, R., & Cha, R. J. (2023). Pediatric Brain Tissue Segmentation Using a Snapshot Hyperspectral Imaging (sHSI) Camera and Machine Learning Classifier. Bioengineering (Basel), 10(10). https://doi.org/10.3390/bioengineering10101190	Tumor segmentation	BaySpec	OCI TM-D2000	Na	Snapshot	475 - 875 nm	13 degrees	Na	35 - 40	FWHM: 12 - 15 nm	500 x 270 Px	Max: 50 fps	Na	Na	Random forest classifier	Sensitivity: 9 %; Specificity: 99.6 %	Intra-operative	Brain	In-vivo	4	Fresh	Reflectance	Pediatric	Tumor segmentation
80	Leon, R., Fabelo, H., Ortega, S., Cruz-Guerrero, I. A., Campos-Delgado, D. U., Szolna, A., Piñeiro, J. F., Espino, C., O'Shanahan, A. J., Hernandez, M., Carrera, D., Bisshopp, S., Sosa, C., Balea-Fernandez, F. J., Morera, J., Clavo, B., & Callico, G. M. (2023). Hyperspectral imaging benchmark based on machine learning for intraoperative brain tumour detection. NPJ Precis Oncol, 7(1), 119. https://doi.org/10.1038/s41698-023-00475-9	Tumor segmentation	Headwall Photonics	Hyperspec® VNIR A-Series	Silicon-based CCD	Line-scanning (pushbroom)	400 - 1000 nm	Na	Na	826 (optimized: 128)	2 - 3 nm (optimized/ sampling: 3.61 nm)	128.7 µm/Px (741 x 1004 Px)	Na	~ 1 min	Broadband halogen [quartz tungsten] (400 - 2200 nm)	Supervised: One-dimensional deep neural network; Random forest classifier; Support vector machine classifier; K-nearest neighbors; Spectral unmixing + Principal component analysis + Hierarchical K-means clustering + K-nearest neighbors filtering + Majority voting	F1-Score: 70.2 %	Intra-operative	Brain	In-vivo	34	Fresh	Reflectance	Adult	Tumor segmentation
81	Lotfy, M., Zhang, X. H., Hauger, C., Giannantonio, T., Alperovich, A., Holm, F., Navab, N., Boehm, F., Schwamborn, C., Hoffmann, T. K., & Schuler, P. J. (2023). Robust Tissue Differentiation in Head and Neck Cancer: Ex-vivo Study with Hyperspectral Imaging and Convolutional Neural Networks (C. A. M. P. B. D. G. G. Tech Univ Munich, R. E. S. D. O. G. Carl Zeiss Meditec Ag, C. R. Carl Zeiss, C. Z. S. D. O. G. Technol, D. O. H. Univ Hosp Ulm, F. D. U. G. Neck Surg, & University, Eds.; WOS:001150986900070; Vol. 12770). Spie-Int Soc Optical Engineering. https://doi.org/10.1117/12.3005006	Tumor segmentation	IMEC	Snapscan VNIR	Silicon-based CCD	Snapshot	450 - 900 nm	Na	Na	91	FWHM: 10 - 15 nm	Max: 3650 x 2048 Px	Na	Na	Na	Convolutional neural network	Accuracy: 96 %	Extra-operative	Head; Neck	Ex-vivo	20	Fresh	Reflectance	Adult	Tumor segmentation
82	Mahmoud, A., & El-Sharkawy, Y. H. (2024). Multi-wavelength interference phase imaging for automatic breast cancer detection and delineation using diffuse reflection imaging. Sci Rep, 14(1), 415. https://doi.org/10.1038/s41598-023-50475-9	Tumor segmentation	Surface Optics	SOC710	Silicon-based CCD	Line-scanning (pushbroom)	380 - 1050 nm	10 degrees	Grating	128 (optimized: 2)	5 nm	4.65 µm/Px	(max/nominal) 100/30 frames/ lines per sec	(max/nominal) 6.96/23.2 seconds/cube	Broadband halogen (380–980 nm)	Proposed method: Multi-wavelength interference phase imaging	Sensitivity: 90.9 %; Specificity: 94 %	Extra-operative	Breast	Ex-vivo	10	Stained; Unstained	Reflectance	Adult	Tumor segmentation
83	Mitsui, T., Mori, A., Takamatsu, T., Kadota, T., Sato, K., Fukushima, R., Okubo, K., Umezawa, M., Takemura, H., Yokota, H., Kuwata, T., Kinoshita, T., Ikematsu, H., Yano, T., Maeda, S., & Soga, K. (2023). Evaluating the identification of the extent of gastric cancer by over-1000 nm near-infrared hyperspectral imaging using surgical specimens. Journal of Biomedical Optics, 28(8), 12. https://doi.org/10.1117/1.Jbo.28.8.086001	Tumor segmentation	Sumitomo Electric Industries	CompoVision CV-N800HS	Na	Na	1000 - 2350 nm	Na	Na	256	6.3 nm	320 Px	100 - 320 fps	Na	Broadband halogen (0.96 W/cm2)	Support vector machine classifier	Accuracy: 79.7 %; Sensitivity: 77.2 %; Specificity: 74.8 %	Extra-operative	Stomach	Ex-vivo	10	Fresh	Reflectance	Adult	Tumor segmentation
84	Mun, N. E., Tran, T. K. C., Park, D. H., Im, J. H., Park, J. I., Le, T. D., Moon, Y. J., Kwon, S. Y., & Yoo, S. W. (2024). Endoscopic Hyperspectral Imaging System to Discriminate Tissue Characteristics in Tissue Phantom and Orthotopic Mouse Pancreatic Tumor Model. Bioengineering (Basel), 11(3). https://doi.org/10.3390/bioengineering11030208	Tumor segmentation	Teledyne QImaging	Retiga R1	Silicon-based CCD	Spectral-scanning	420 - 730 nm	11.1 mm	Na	Na	Na	18.0 line pairs per millimeter; 6.45 µm x 6.45 µm (per pixel) (1360 x 1024 Px)	Na	Na	LED	Support vector machine classifier; Shallow neural network; Light gradient boosting machine	**KPC tumor:** Precision [LGBM]: 89.6 %; Recall [LGBM]: 61.7 %; F-score [LGBM]: 73.1 %; **Pan02 tumor:** Precision [SVM]: 83.0 %; Recall [SVM]: 50.9 %; F-score [SVM]: 63.1 %	Extra-operative	Pancreas	In-vivo (animal); Phantom	0	Fresh; Phantom	Reflectance	Animal (mouse)	Tumor segmentation
85	Suero Molina, E., Black, D., Walke, A., Azemi, G., D'Alessandro, F., König, S., & Stummer, W. (2023). Unraveling the blue shift in porphyrin fluorescence in glioma: The 620nm peak and its potential significance in tumor biology. Front Neurosci, 17, 1261679. https://doi.org/10.3389/fnins.2023.1261679	Fluorescence quantification; Autofluorescence quantification	Experimental	Experimental	CMOS	Na	420 - 730 nm	Na	Liquid crystal tunable filter	Na	3 - 5 nm	2048 x 2048 PX	Na	Na	LED (405 nm + Broadband white); Blue light fluorescence module/filter [Zeiss BLUE 400]	Spectral unmixing	Na	Extra-operative	**Human:** Brain; **Animal (pig):** Brain (homogenate); Phantom	Ex-vivo (animal and human); Phantom	130 (Careful! Overestimation)	**Human:** Fresh; **Animal (pig):** Homogenized, 0.5 M trishydroxymethylaminomethane buffer (prepared with hydrochloric acid), pH 5 - 9; (liquid) Phantom	Exogenous Fluorescence (5-ALA)	Adult; Animal (pig)	Fluorescence quantification; Autofluorescence quantification
